# Design, synthesis, and biological evaluation of naphthalimide derivatives as dual monoamine oxidase-B and amyloid-β aggregation inhibitors for Alzheimer's disease

**DOI:** 10.1039/d6ra07131g

**Published:** 2026-10-06

**Authors:** Rukshar Praveen, Alessandro Gadaleta, Gabriella La Spada, Daniela Valeria Miniero, Aquib Reza, Tohasib Yusub Chaudhari, Abdur Rahman, Marco Catto, Nasimul Hoda

**Affiliations:** a Drug Design and Synthesis Lab., Department of Chemistry, Jamia Millia Islamia New Delhi-110025 India nhoda@jmi.ac.in +0091-11-26985507 +0091-9910200655; b Department of Pharmacy-Pharmaceutical Sciences, University of Bari Aldo Moro Via E. Orabona 4 70125 Bari Italy marco.catto@uniba.it; c Department of Medicine and Surgery, LUM University Giuseppe Degennaro Torre Rossi 70010 Casamassima Italy; d Department of Molecular Medicine, Jamia Hamdard New Delhi 110062 India

## Abstract

Alzheimer's disease (AD) is a chronic and progressive neurodegenerative disorder with multifactorial pathogenesis. Amyloid beta (Aβ) and monoamine oxidase (MAO) are among the key therapeutic targets for its treatment. In this investigation, a series of 18 new naphthalimide-based derivatives were synthesized and assessed for multitarget activity against hAChE, esBuChE, MAO-A, MAO-B, Aβ, and paired helical fragment 6 (PHF6) aggregation. The screened compounds predominantly exhibited dual inhibition of MAO-B and Aβ aggregation to counteract oxidative stress and plaque formation. Among the tested compounds, P12 and P17 were found to have remarkable inhibition against Aβ, with 83% and 91% inhibition, respectively, compared to the reference compound quercetin (75% inhibition). The compound P10 was the most effective MAO-B inhibitor, with an IC_50_ value of 3.09 ± 0.10 µM. Cytotoxicity studies in SH-SY5Y cells confirmed that compounds were non-toxic up to 25 µM. Additionally, the neuroprotection assays revealed that P10 was capable of reducing oxidative stress effectively against H_2_O_2_-induced toxicity, while P12 showed superior protection against Aβ-induced neurotoxicity in SH-SY5Y cells. Furthermore, *in silico* investigations provided structural insights into the predicted ligand-target interaction modes and dynamic behavior of the selected compounds with MAO-B and monomeric Aβ1-42. Consequently, all these findings underscore that naphthalimide derivatives are promising dual inhibitors of amyloid-β aggregation and MAO-B and have potential for further development as MTDLs for AD.

## Abstract 2

Herein, we report the synthesis and evaluation of 18 novel naphthalimide-based derivatives for their activity against hAChE, esBuChE, MAO-A, MAO-B, Aβ, and PHF6 aggregation.

## Graphical abstract

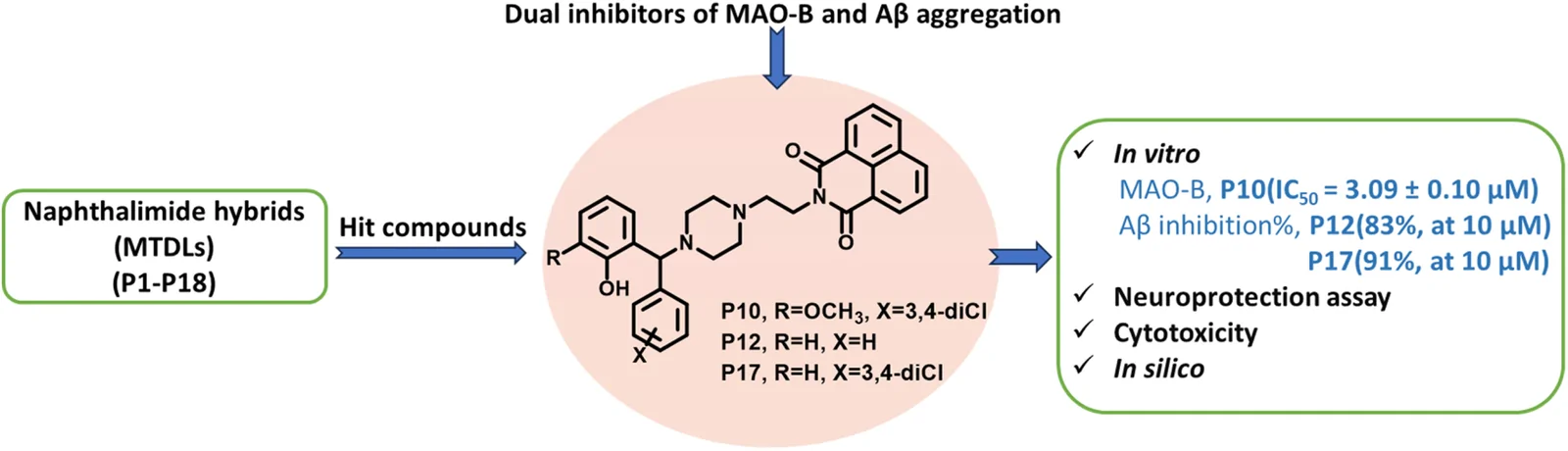

## 1. Introduction

Alzheimer's disease (AD) is a chronic, progressive neurodegenerative disorder first identified by Alois Alzheimer in 1906. It is clinically characterized by gradual memory impairment, cognitive deterioration, language deficits, behavioral disturbances, and personality changes. It was reported that over 57 million people worldwide are currently living with AD and other forms of dementia.^1^ This number is growing rapidly and is expected to triple to 153 million by 2050, thereby highlighting that AD is a devastating disease that mainly affects elderly individuals. In 2020 and 2021, when COVID-19 emerged as a top ten cause of death, AD ranked as the seventh most prevalent cause of death in the United States.^2^ Death rates for AD increased by over 142%, while deaths from stroke, HIV, and heart diseases decreased between 2000 and 2022. Consequently, the rising incidence of AD will increase both financial and psychosocial burdens worldwide. Therefore, there is an urgent need to address this global issue through the development of new therapeutic strategies. However, the primary hurdle is the unclear etiology of AD.^3^ Despite extensive research, the exact causes of AD remain unknown; however, multiple factors have been implicated in its development, including cholinergic dysfunction, neuroinflammation, oxidative stress, neurofibrillary tangles, amyloid plaques, mitochondrial dysfunction, and monoamine oxidase hyperactivation, which are believed to contribute significantly to disease progression.^4–6^

Among the multifaceted hypotheses of AD, amyloid beta (Aβ) plaques and neurofibrillary tangles (NFTs) are the two key pathological hallmarks. Aggregated Aβ peptides accumulate extracellularly to form Aβ plaques, while NFTs are intracellular aggregates of hyperphosphorylated tau protein.^7^ Tau is an axonal microtubule-associated protein, essential for neuronal structure and function. In AD, aberrant phosphorylation reduces tau binding affinity, allowing excess tau to misfold and aggregate into toxic NFTs, triggering the death of neuronal cells.^8^ Aβ accumulation and aggregation in the brain form senile plaques, leading to cognitive decline and neurodegeneration. Aβ plaques are extracellular deposits of aggregated Aβ peptides of various lengths, mostly Aβ1-42, generated *via* the amyloidogenic processing of amyloid precursor protein (APP), which is sequentially cleaved by β-secretase (β-APP-cleaving enzyme-1 (BACE1)) and γ-secretase.^9^ Aβ aggregation is a key early event that promotes downstream AD pathologies such as neurofibrillary tangles, senile plaques, dementia, and neuronal death. Aβ oligomers, forming as intermediates of the aggregation cascade, act as the real neurotoxic species responsible for neurodegeneration, therefore, suppression of Aβ aggregation represents an attractive therapeutic strategy for the treatment of AD. However, all the preclinical studies with anti-aggregating small molecules resulted in unsuccessful results, and the recent introduction of immunotherapy with anti-oligomer monoclonal antibodies (*i.e.*, aducanumab, lecanemab, donanemab) is still a matter of debate.^2^

In addition to amyloid pathology, monoamine oxidases (MAOs) are a significant factor contributing to oxidative stress associated with AD. MAOs are flavin adenine dinucleotide (FAD)-dependent enzymes that catalyze the oxidative deamination of endogenous and xenobiotic monoamines, including key neurotransmitters such as dopamine, serotonin, norepinephrine, and epinephrine in the brain. These metabolic processes contribute to oxidative stress by producing corresponding hydrogen peroxide, aldehydes, and other reactive oxygen species (ROS).^10^ Overproduction of ROS may induce neuronal cell death and is closely linked with the initiation and progression of AD. MAO-A and MAO-B are the two isoforms of MAOs, which are encoded by distinct genes and differ in tissue distribution, substrate specificity, and inhibitor selectivity. Selective MAO-A inhibitors are utilized as antidepressants, whereas MAO-B inhibitors such as rasagiline and selegiline have been widely employed in Parkinson's disease and are increasingly explored for AD. Notably, MAO-B levels are significantly increased in the aging brain as well as in patients with AD. The heightened activity of this enzyme contributes to the overproduction of ROS, impairs cholinergic neurotransmission, and accelerates amyloid-related pathological processes.^11^ Therefore, selective and preferably reversible MAO-B inhibition represents a promising approach to attenuate oxidative stress-driven neurodegeneration.

### 1.1. Design strategy

The naphthalimide framework has emerged as a versatile and pharmacologically valuable scaffold in AD drug discovery because of its synthetic accessibility, structural tunability, and planar π-conjugated architecture, which facilitates π–π interactions with amyloid assemblies and effective accommodation within enzyme active sites. Increasing evidence suggests that naphthalimide derivatives can modulate multiple AD-relevant targets, including MAO, cholinesterases, and the aggregation of tau and Aβ. According to the approach taken in designing our molecules, which is depicted in Fig. 1, recent research highlights its translational potential in AD. Recently, the study by Mondal *et al.*^12^ reported a naphthalimide-based scaffold A as shown in Fig. 1, capable of forming ultralong fibers targeting toxic Aβ oligomers. These fibers reduce oxidative stress and neuronal damage, cross the blood–brain barrier, and reduce neurotoxicity *in vivo*, offering a promising therapeutic strategy for AD. Likely, Sidhu *et al.*^13^ developed naphthalimide-based fluorogenic probe B (Fig. 1), including Naph-3, which selectively detects acetylcholinesterase (AChE) activity by mimicking acetylcholine and exhibits a significant fluorescence enhancement upon binding with AChE, providing a robust and selective tool for real-time AChE monitoring in neuronal systems. Similarly, Sun *et al.*^14^ reported a two-photon naphthalimide probe C (Fig. 1) for simultaneous detection of hydrogen sulfide (H_2_S) and ATP in AD brains, demonstrating H_2_S regulation of glutamate-induced ATP dysregulation linked with neurodegeneration. In addition, Pasieka *et al.* demonstrated that dual inhibitor hybrids D (Fig. 1) with diphenylmethyl groups effectively inhibit tau and Aβ aggregation, suggesting a promising approach for AD targeting.^15^ Moreover, Piemontese *et al.*^16^ employed a piperazine linker in donepezil-based hybrids E (Fig. 1) to maximize pharmacophore orientation, improving AChE inhibition, anti-amyloid activity, antioxidant capacity, and metal-chelating qualities. Therefore, these findings emphasize the significance of multifunctional hybrid architectures in the development of anti-AD drugs.

**Fig. 1:**
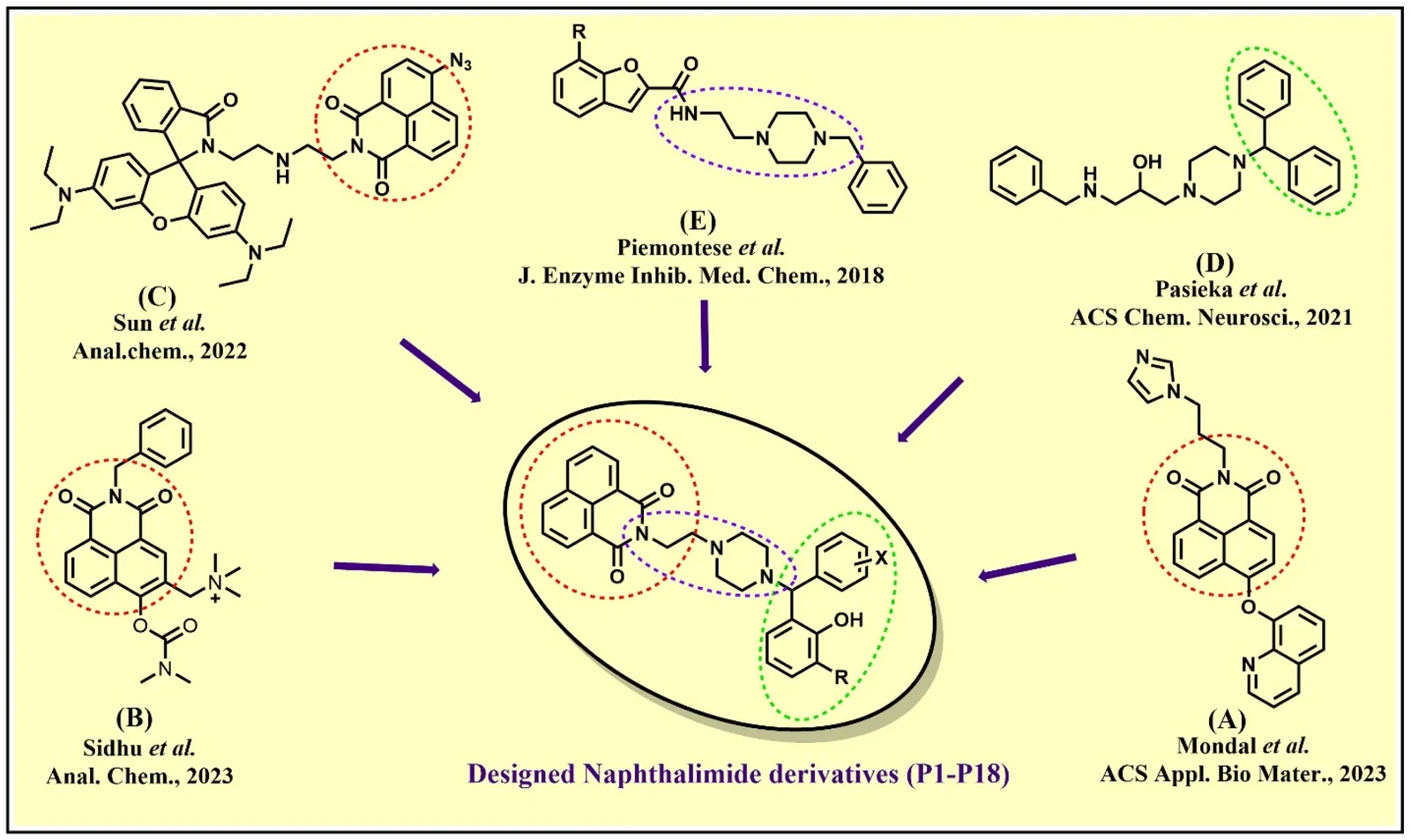
Rationale for the design strategy of naphthalimide-based derivatives.

Guided by insights from the literature and addressing the multifactorial origin of AD, we employed a multitarget-directed ligand (MTDL) technique to construct dual MAO-B and Aβ aggregation inhibitors inside a single chemical framework. A molecular hybridization approach was applied to link a physiologically active, planar naphthalimide core with a diphenyl pharmacophore through a flexible piperazine spacer, therefore improving conformational flexibility and target contact. To enhance biological efficacy, electron-donating and electron-withdrawing groups were systematically substituted for one phenyl ring at *ortho*- and *para*-positions.

This study involves the design, synthesis, and assessment of a series of 18 naphthalimide-based hybrids that serve as dual inhibitors of MAO and Aβ aggregation. Their biological activities were also tested against acetyl- and butyryl-cholinesterase (AChE and BuChE), as well as PHF6 aggregation, a mimic of tau protein aggregation. The neuroprotective effects of the most active compounds were examined for their ability to protect against Aβ and H_2_O_2_-induced toxicity in SH-SY5Y cells. Additionally, *in vitro* cytotoxicity tests were performed on these compounds using SH-SY5Y cells. To strengthen the findings, molecular docking studies were carried out to analyze ligand–protein interactions, followed by molecular dynamics simulations to assess the stability and behavior of the protein–ligand complexes. To evaluate pharmacokinetic properties, the physicochemical and ADME characteristics of the most active compounds were also analyzed.

## 2. Results and discussion

### 2.1. Chemistry

The suitable synthetic route for preparing the designed naphthalimide derivatives is shown in Schemes 1 and 2. Firstly, commercially available 1,8-naphthalic anhydride (1) was refluxed with *N*-(2-aminoethyl) piperazine (2) in toluene for 12 hours under inert conditions, using triethylamine as a base. This condensation reaction produced the key intermediate 2-(2-(piperazin-1-yl)ethyl)-1*H*-benzo[de]isoquinoline-1,3(2*H*)-dione (3) as shown in Scheme 1 in good yield (83%). In Scheme 2, intermediate 3 was reacted with various aromatic aldehydes (4) and substituted aromatic boronic acids (5) in anhydrous DMF through a multicomponent Petasis reaction, conducted by refluxing in a one-pot procedure, resulting in the desired final compounds (P1–P18) in good yield. Column chromatography was used to purify the synthesized compounds, and mass spectrometry, ^1^H NMR, and ^13^C NMR were used to confirm their structures.

**Scheme 1:**
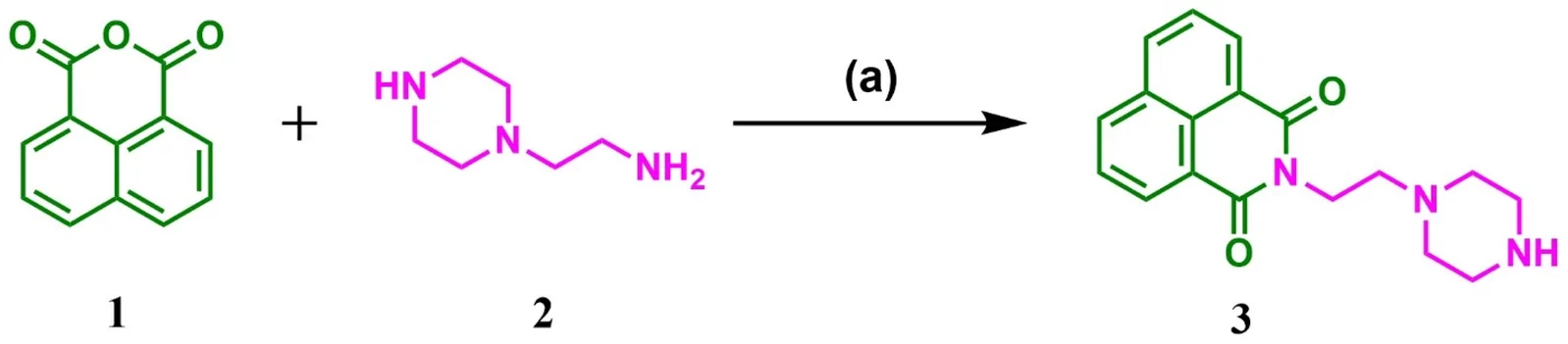
Reagents and conditions: (a) toluene, triethylamine, reflux, 100–110 °C, 12 h, 83%.

**Scheme 2:**
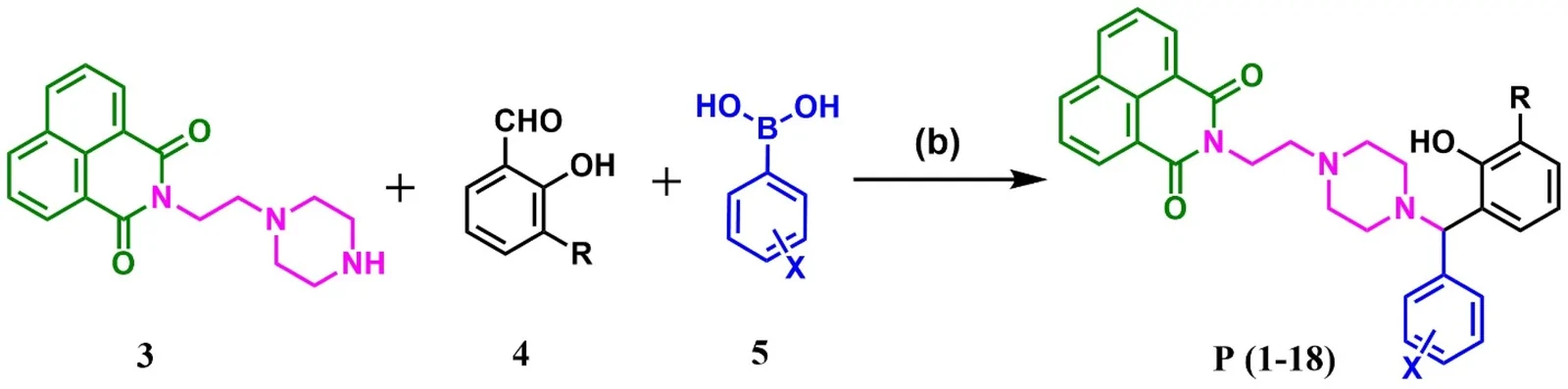
Reagents and conditions: (b) dimethylformamide (DMF), 90 °C, N_2_ atm, 10–12 h.

### 2.2. *In vitro* biological assays

#### 2.2.1. AChE, BuChE, MAO, Aβ, and PHF6 aggregation inhibitory experiments

Compounds P1–P18 were tested *in vitro* at a screening concentration of 10 µM to evaluate their inhibitory activity against key targets involved in AD progression, including hAChE, esBuChE, MAO-A, MAO-B, Aβ, and PHF6 aggregation. The oligopeptide PHF6 is the highly proaggregating sequence (306)VQIVYK(311) present in the full-length tau protein with high propensity to arrange to β-sheet form.^17^ For this feature, it is a widely accepted mimic of tau protein for routine *in vitro* assays.

Results are summarized in Table 1 (IC_50_ values were calculated only for compounds showing more than 60% inhibition at a single concentration of 10 µM) as percentage inhibition values, with donepezil (AChE/BuChE), clorgyline (MAO-A), safinamide (MAO-B), and quercetin (Aβ and PHF6 aggregation) used as reference standards. Fluorimetric measurements were not affected by intrinsic fluorescence of compounds, thus avoiding possible interference from pan-inhibitors (PAINS). The data from our compound library demonstrated moderate to potent dual inhibitory activity, with MAO-B inhibition reaching up to 64%, and inhibition of Aβ aggregation up to 83% and 91% for the most active derivatives. Notably, 11 out of 18 compounds showed more than 50% inhibition of Aβ aggregation, while only three compounds exceeded 50% inhibition of MAO-B. Additionally, six compounds inhibited PHF6 aggregation by over 50%, while only two compounds showed comparable activity against hAChE. Most derivatives displayed little to no inhibitory activity against esBuChE and MAO-A.

**Table 1:** *In vitro* AChE, BuChE, MAO-A, MAO-B, Aβ, and PHF6 aggregation inhibition data for synthesized compounds P1–P18

Code	R	X	% Inhibition at 10 µM^a^					
			hAChE	esBChE	MAO A	MAO B	Aβ40 aggrn.	PHF6 aggrn.
P1	OCH_3_	4-F	27 ± 6	ni	29 ± 4	13 ± 2	72 ± 4	ni
P2		H	19 ± 1	ni	24 ± 4	27 ± 4	ni	ni
P3		3-Cl, 4-F	22 ± 5	ni	29 ± 5	33 ± 1	23 ± 5	21 ± 6
P4		3,4-diF	27 ± 6	ni	34 ± 5	18 ± 1	ni	46 ± 7
P5		4-CH_3_	48 ± 4	ni	34 ± 3	46 ± 2	47 ± 3	14 ± 1
P6		4-Cl	27 ± 5	ni	25 ± 1	18 ± 1	52 ± 3	25 ± 6
P7		4-OCH_3_	52 ± 1	ni	37 ± 5	51 ± 6	40 ± 6	54 ± 7
P8		4-CF_3_	25 ± 5	ni	32 ± 6	58 ± 5	56 ± 2	ni
						IC_50_ 5.55 ± 0.65 µM		
P9		4-OCF_3_	7 ± 2	ni	30 ± 5	48 ± 5	25 ± 5	ni
P10		3,4-diCl	23 ± 1	ni	24 ± 2	64 ± 1	52 ± 6	52 ± 6
						IC_50_ 3.09 ± 0.10 µM		
P11	H	4-F	ni	ni	ni	ni	54 ± 6	55 ± 3
P12		H	27 ± 6	ni	18 ± 4	11 ± 5	83 ± 3	55 ± 3
P13		3-Cl, 4-F	12 ± 1	ni	ni	10 ± 4	71 ± 7	48 ± 8
P14		4-CH_3_	12 ± 2	ni	ni	ni	49 ± 6	25 ± 5
P15		4-Cl	11 ± 1	42 ± 5	ni	ni	59 ± 5	55 ± 8
P16		4-OCH_3_	18 ± 3	9 ± 5	ni	ni	67 ± 4	42 ± 6
P17		3,4-diCl	36 ± 4	27 ± 5	ni	30 ± 5	91 ± 6	60 ± 6
P18^b^	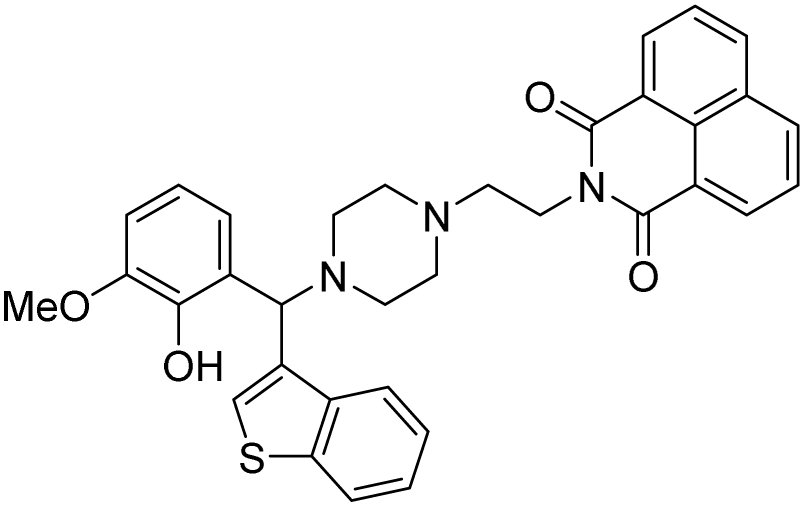		52 ± 1	ni	9 ± 5	15 ± 6	70 ± 6	33 ± 7
Donepezil			93 ± 1	81 ± 2				
Clorgyline					100			
Safinamide						100		
Quercetin			—	—			75 ± 5	83 ± 6

a Values are mean ± SEM of three experiments, ni: no inhibition.

b Tested at 9.5 µM.

Structure–activity relationship analysis revealed that substitution at the 3- and 4-positions of the phenyl rings within the diphenyl scaffold plays a crucial role in modulating activity. First, we noticed that the presence of a 3,4-dichloro substituent on one phenyl ring was more beneficial for modulating activity for both MAO-B and aggregation inhibition. Among the series, P17, bearing the 3,4-dichloro group, showed the highest PHF6 aggregation inhibition (60%). In contrast, replacement of 3,4-dichloro with other substituents such as 4-OCH_3_, 4-Cl, 4-F, or H and 4-H with 4-OCH_3_ in another phenyl ring resulted in a marked reduction in activity (≤50%). In contrast, most compounds showed strong inhibitory activity against MAO-B and Aβ aggregation, while showing only weak inhibition of MAO-A. Table 1 illustrates the strong influence of phenyl ring substitutions on both MAO-B and Aβ aggregation inhibition. Notably, P8 and P10, bearing 4-CF_3_ and 3,4-dichloro substituents, respectively, on one phenyl ring, displayed significant MAO-B inhibition with an IC_50_ value of 5.55 ± 0.65 µM and 3.09 ± 0.10 µM, respectively, suggesting a favorable contribution of electron-withdrawing groups on MAO-B activity. In fact, the replacement of the 4-CF_3_ substituent in P8 with the 4-CH_3_ in P5, 4-OCH_3_ in P7, and 4-OCF_3_ in P9 resulted in a slight reduction of MAO-B inhibitory activity. Further evaluation of the compounds at the 10 µM concentration revealed moderate to strong inhibition of amyloid-β aggregation, ranging from 23% to 91%. Importantly, 5 out of 18 compounds showed more than 70% inhibition. Among them, P12 with 4-H and P17 with 3,4-dichloro were found to be the most potent inhibitors of Aβ aggregation of this series, with 83% and 91% inhibition, respectively, and are stronger inhibitors than quercetin (75% inhibition), the reference compound, as mentioned in Table 1. Furthermore, the other substitution on the phenyl ring reduces Aβ activity by up to 50%.

In the reported series of compounds, P10 was identified as the most effective MAO-B inhibitor, whereas P12 and P17 were identified as the most potent inhibitors of Aβ aggregation. Collectively, these results highlight P10, P12, and P17 as promising leads with dual-target activity, meriting further optimization for the development of multifunctional MAO-B/Aβ aggregation inhibitors.

#### 2.2.2. Cytotoxicity studies

The cytotoxic profiles of the most active compounds (P10, P12, and P17) were examined in SH-SY5Y cells using the MTT assay. The compounds were evaluated across a concentration range of 0.1–100 µM, and cell viability was assessed after 24 h of exposure. As illustrated in Fig. 2, all tested compounds showed negligible cytotoxic effects at lower dosages and were well tolerated up to 50 µM. While only a minor reduction in cell viability was found at higher concentrations range up to 50–100 µM, P10 demonstrated a concentration-dependent decrease, with cell viability dropping to approximately 50% at 100 µM. Notably, at dosages up to 10 µM, all the tested compounds retained nearly 100% cell viability relative to untreated controls.

**Fig. 2:**
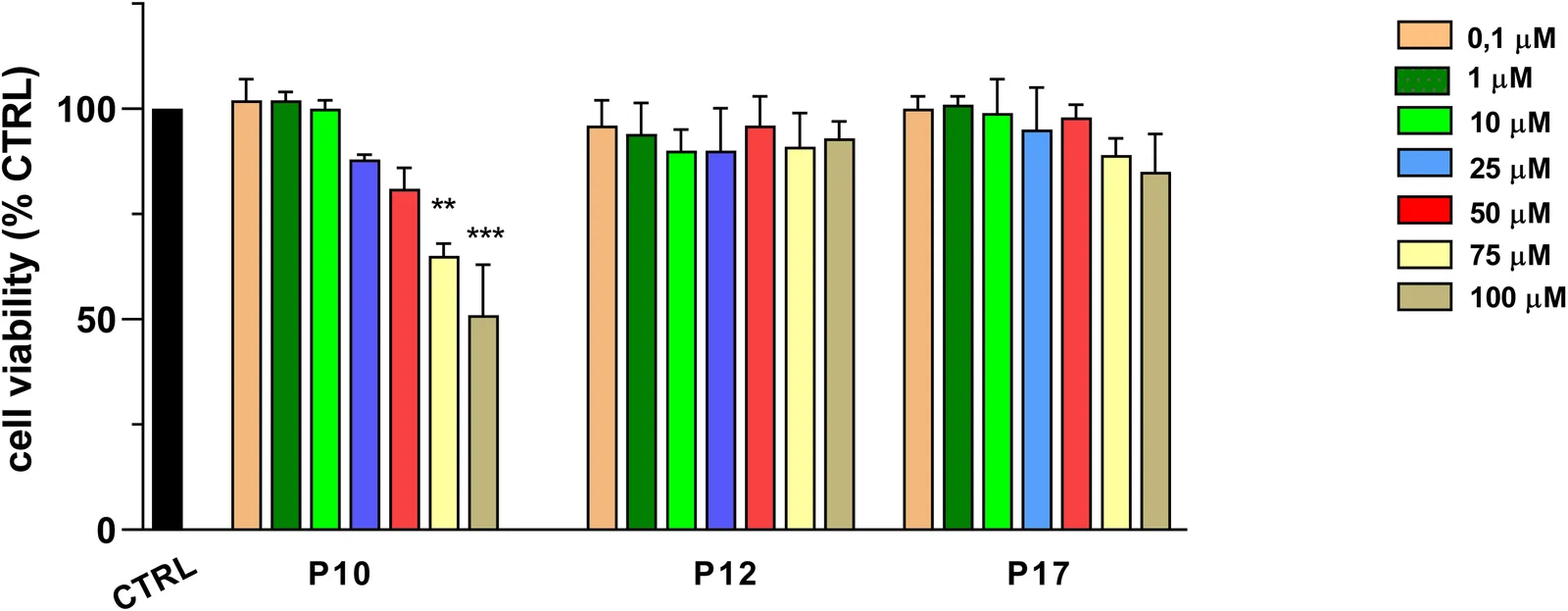
Cytotoxicity studies of compounds P10, P12, and P17 on the SH-SY5Y cells after treatment for 24 h. One-way analysis of variance (ANOVA) followed by multiple comparison tests (Dunnet's test). Levels of significance: **p < 0.001; ***p < 0.0001 referred to CTRL.

Given that the IC_50_ values obtained from enzyme inhibition studies fell within the low micromolar range, our findings suggest that the compounds display low cytotoxicity and are well tolerated in neuronal cells at experimentally tested concentrations.

#### 2.2.3. Protection against Aβ42-induced cytotoxicity

Aβ is a significant component of the pathogenesis of Alzheimer's disease, producing neuronal damage through oxidative stress, mitochondrial dysfunction, and disturbance of calcium homeostasis, ultimately leading to apoptotic cell death. To replicate this effect, SH-SY5Y cells were treated with Aβ42, generating an *in vitro* system of Aβ-induced neurotoxicity. As indicated in Fig. 3, exposure to Aβ42 (20 µM) for 24 h drastically reduced cell viability to around 65–70% of control, demonstrating its profound cytotoxic impact, likely coupled with higher intracellular ROS levels and poor cellular metabolism. The neuroprotective potential of selected compounds was then examined, with P10 (a potent MAO-B inhibitor) and P12 and P17 (the most active inhibitors of Aβ aggregation), throughout a concentration range of 0.1–10 µM, using quercetin as a reference molecule. Among these, P12 displayed the strongest protective effect, notably at 10 µM, where it considerably restored cell viability, indicating effective mitigation of Aβ-induced toxicity. In contrast, P10 and P17 demonstrated only limited protection, with minor improvements in cell survival. Thus, these data identify P12 as the most viable candidate for counteracting Aβ-induced neurotoxicity within this series.

**Fig. 3:**
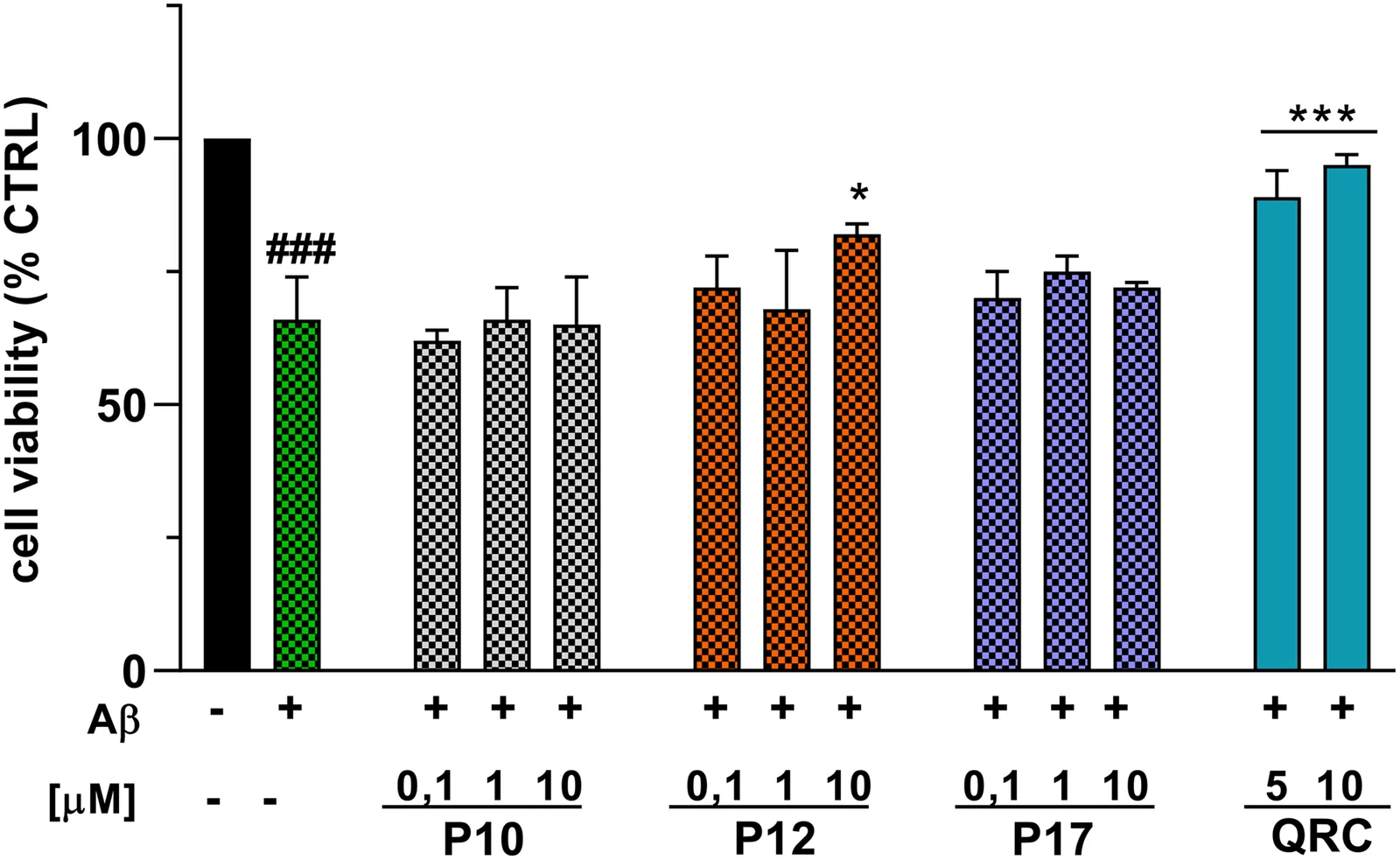
Aβ-induced neuroprotective studies of compounds P10, P12, and P17 on the SH-SY5Y cells after treatment for 24 h. One-way analysis of variance (ANOVA) followed by multiple comparison tests (Dunnet's test). Levels of significance: ^###^*p* < 0.0001 referred to untreated cells; *p < 0.05 and ***p < 0.0001 referred to Aβ.

#### 2.2.4. Protection against H_2_O_2_-induced cytotoxicity

Hydrogen peroxide (H_2_O_2_), a prominent reactive oxygen species formed during oxidative stress and as a byproduct of MAO-B activity, is known to contribute to neuronal injury and to promote Aβ aggregation. Increased levels of H_2_O_2_ can accelerate Aβ misfolding and oligomer formation, thereby intensifying oxidative damage associated with elevated MAO-B activity. As depicted in Fig. 4, exposure of SH-SY5Y cells to H_2_O_2_ (300 µM concentration) for 24 h led to a significant reduction in cell viability up to 65% of control, confirming the establishment of an oxidative stress-induced cytotoxic model. Co-incubation with the most active compounds, P10, P12, and P17 in the concentration range from 0.1–10 µM resulted in concentration-dependent protection against H_2_O_2_-induced damage. A preliminary experiment to disclose a possible intrinsic antioxidant potency was done by means of 2,2-diphenyl-1-picrylhydrazyl (DPPH) radical scavenging assay, which resulted in complete absence of antioxidant efficacy (data not shown).

**Fig. 4:**
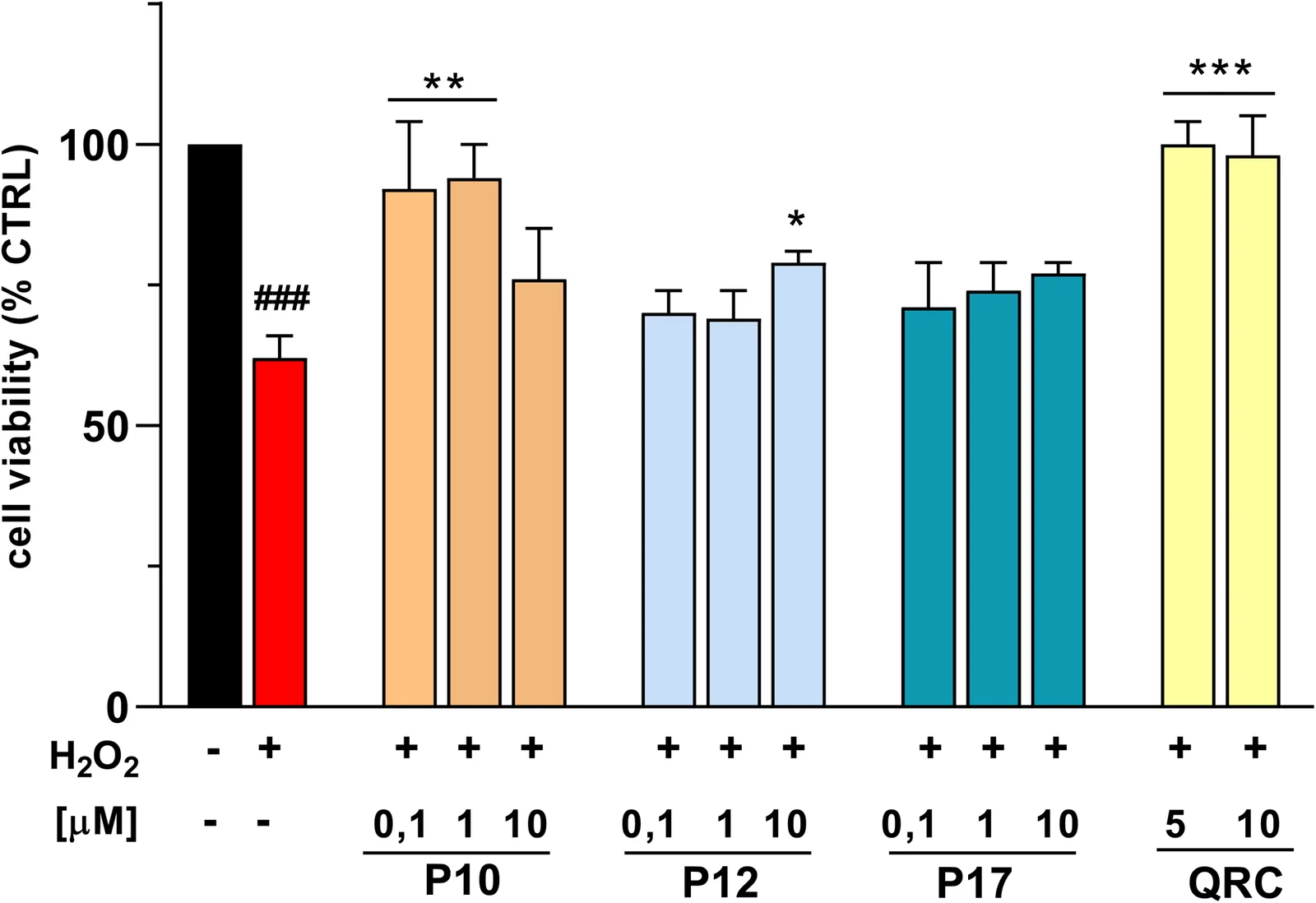
H_2_O_2_-induced neuroprotective studies of tested compounds on the SH-SY5Y cells after treatment for 24 h. One-way analysis of variance (ANOVA) followed by multiple comparison tests (Dunnet's test). Levels of significance: ^###^*p* < 0.0001 referred to untreated cells; *p < 0.05, **p < 0.001 and ***p < 0.0001 referred to H_2_O_2_.

Among the tested compounds, P10 exhibited the strongest cytoprotective effect, significantly improving cell viability at 1 µM, although a slight decline was observed at 10 µM. In comparison, P12 and P17 demonstrated moderate protective effects, with partial restoration of cell viability. The reference compound quercetin (5 and 10 µM) provided the highest level of protection, restoring cell viability close to that of untreated cells.

Collectively, these results indicate that compounds, particularly P10, that reduce H_2_O_2_-induced oxidative stress may also influence oxidative stress-related pathways and Aβ aggregation, supporting their development as potential multifunctional neuroprotective agents.

### 2.3. *In silico* studies

#### 2.3.1. Molecular docking studies

To investigate the putative binding orientations and interaction patterns of the selected compounds, P10 was docked against MAO-B (PDB ID: 2V61), whereas P12 and P17 were docked against the experimentally determined monomeric Aβ1-42 structure (PDB ID: 1IYT). Docking poses were ranked according to the predicted binding affinity obtained using AutoDock Vina, where more negative scores indicate more favorable predicted ligand–target interactions within the Vina scoring function. These docking scores should be considered computational predictions and do not directly represent experimental binding affinities or complex stability. The 0.000 Å RMSD values reported for the top-ranked poses represent the reference pose used for pose comparison in AutoDock Vina and should not be interpreted as an independent measure of docking accuracy or experimental validation. The listed docking scores are shown in Table 2.

**Table 2:** Binding affinities of the most active ligands: P10 against MAO-B (PDB ID: 2V61; Safinamide ligand was used as reference), P12 and P17 against Aβ (PDB ID: 1IYT; Quercetin ligand was used as reference)

PDB ID	Compounds	Binding affinity (kcal mol^−1^)	RMSD l.b (Å)	RMSD u.b (Å)
2V61	P10	−7.3	0.000	0.000
	Safinamide (reference)	−9.2	0.000	0.000
1IYT	P12	−7.2	0.000	0.000
	P17	−7.2	0.000	0.000
	Quercetin (reference)	−5.2	0.000	0.000

Table 2 shows that P10 exhibited a favorable predicted binding affinity toward MAO-B, with an AutoDock Vina docking score of −7.3 kcal mol^−1^. Safinamide, used as the reference ligand, showed a more favorable predicted binding score of −9.2 kcal mol^−1^. P12 and P17 exhibited comparable predicted binding affinities toward Aβ, with identical docking scores of −7.2 kcal mol^−1^. Quercetin, used as the reference compound for the Aβ docking analysis, showed a docking score of −5.2 kcal mol^−1^. Thus, P12 and P17 exhibited more favorable predicted docking scores than quercetin within the AutoDock Vina scoring model. Although P12 and P17 showed identical docking scores, their predicted interaction patterns differed, as illustrated in Fig. 6 and 7. The identical docking scores should therefore be interpreted as comparable computational docking scores rather than evidence of identical experimental binding affinities.

The predicted binding mode of P10 showed that the ligand was accommodated within the MAO-B binding pocket, as illustrated in Fig. 5. The P10–2V61 complex exhibited several predicted ligand-residue interactions. Conventional hydrogen-bond interactions were observed between the carbonyl oxygen of the naphthalimide ring and ARG42, THR43, and THR426. TYR60, GLY205, and GLY434 showed predicted carbon-hydrogen bond interactions involving the methoxy group and the piperazine moiety, while CYS397 showed a predicted halogen interaction with the chlorine atom of the phenyl ring. GLY13 interacted with the naphthalimide benzene ring through a predicted π–sigma interaction, while CYS397 showed a predicted π–sulfur interaction with the phenyl ring. Additional predicted interactions included a π–π T-shaped interaction with TRP398, amide–π stacking with GLY57, a π–cation interaction with LYS296, and alkyl/π–alkyl interactions involving TRP388, PHE343, VAL294, LYS296, TYR435, PRO265, ALA439, and ARG42. GLY58 was also involved through predicted van der Waals interactions. Collectively, these interactions provide a structural basis for the predicted accommodation of P10 within the MAO-B binding pocket.

**Fig. 5:**
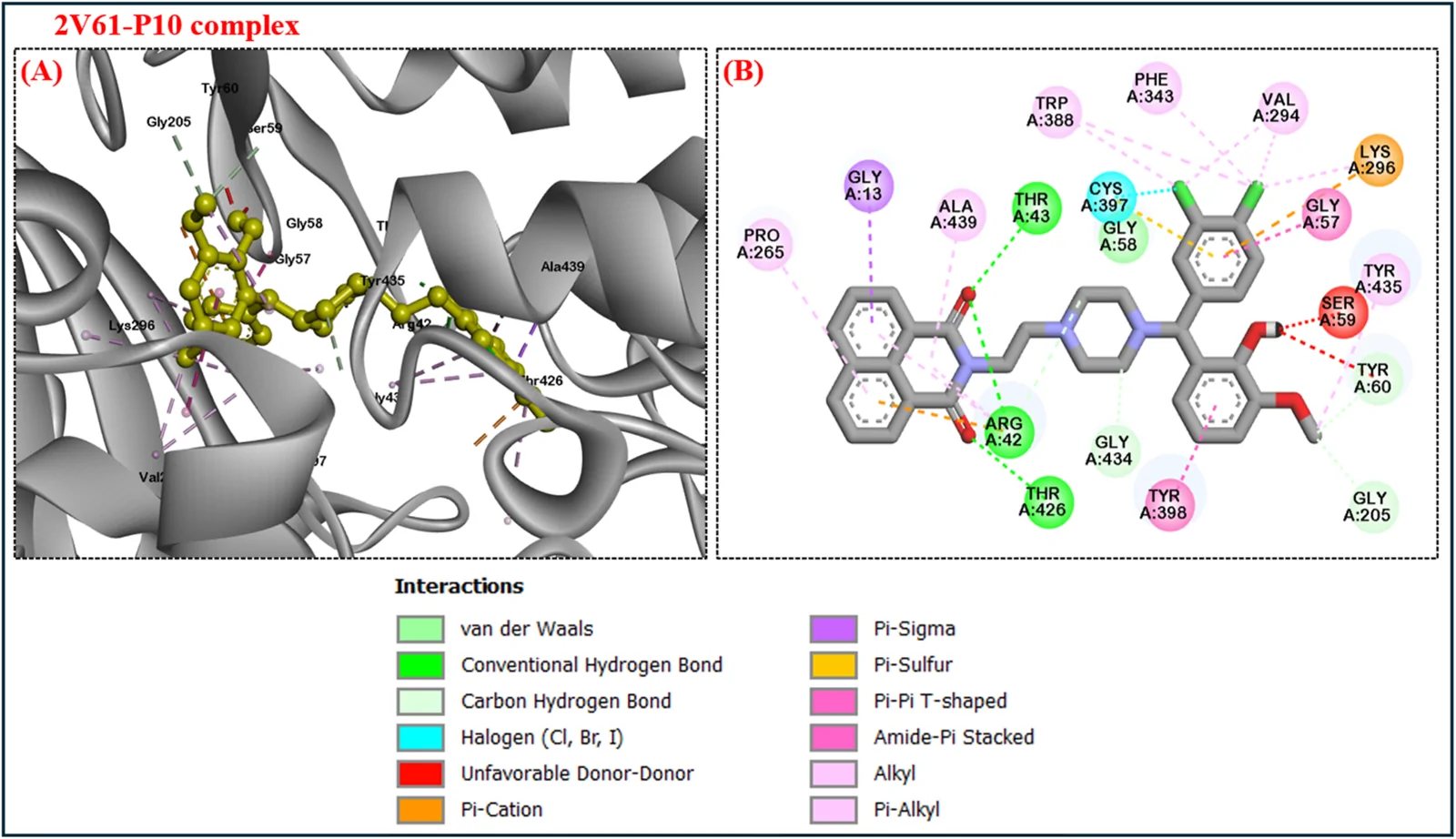
Molecular docking pose 3D (A) and 2D (B) of P10 with protein MAO-B (PDB ID:2V61) surrounded by different amino acid residues represented by several colors.

Similarly, docking analysis of the complexes P12–1IYT and P17–1IYT showed predicted ligand–residue interaction patterns involving the monomeric Aβ1-42 structure, including a conventional hydrogen bond between the –OH group of the phenyl ring and ALA21, as shown in Fig. 6 and 7. Additionally, for both ligands P12 and P17, amide–π stacked interactions with GLY38, LEU34, and PHE20, along with π–alkyl interactions with VAL24, ALA21, and LEU34, were observed in the docking poses. Moreover, surrounding residues in compound P12, including GLY25, ILE31, MET35, VAL39, ILE41, and ALA42, were predicted to contribute through van der Waals interactions, while in P17, MET35 exhibited a predicted π–sulfur interaction, alongside LEU17 and PHE20, which participated in predicted alkyl interactions.

**Fig. 6:**
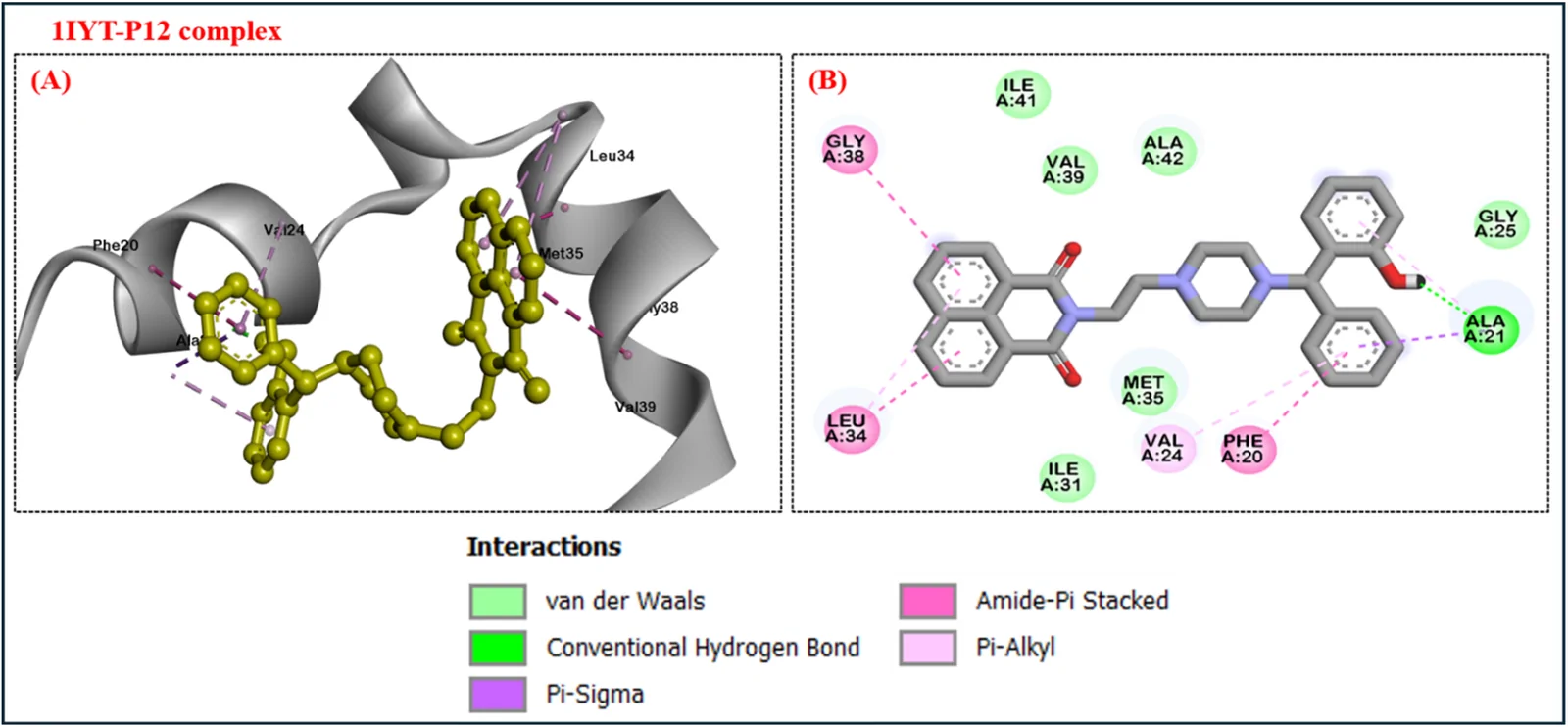
Molecular docking pose 3D (A) and 2D (B) of P12 with protein Aβ (PDB ID:1IYT) surrounded by different amino acid residues represented by several colors.

**Fig. 7:**
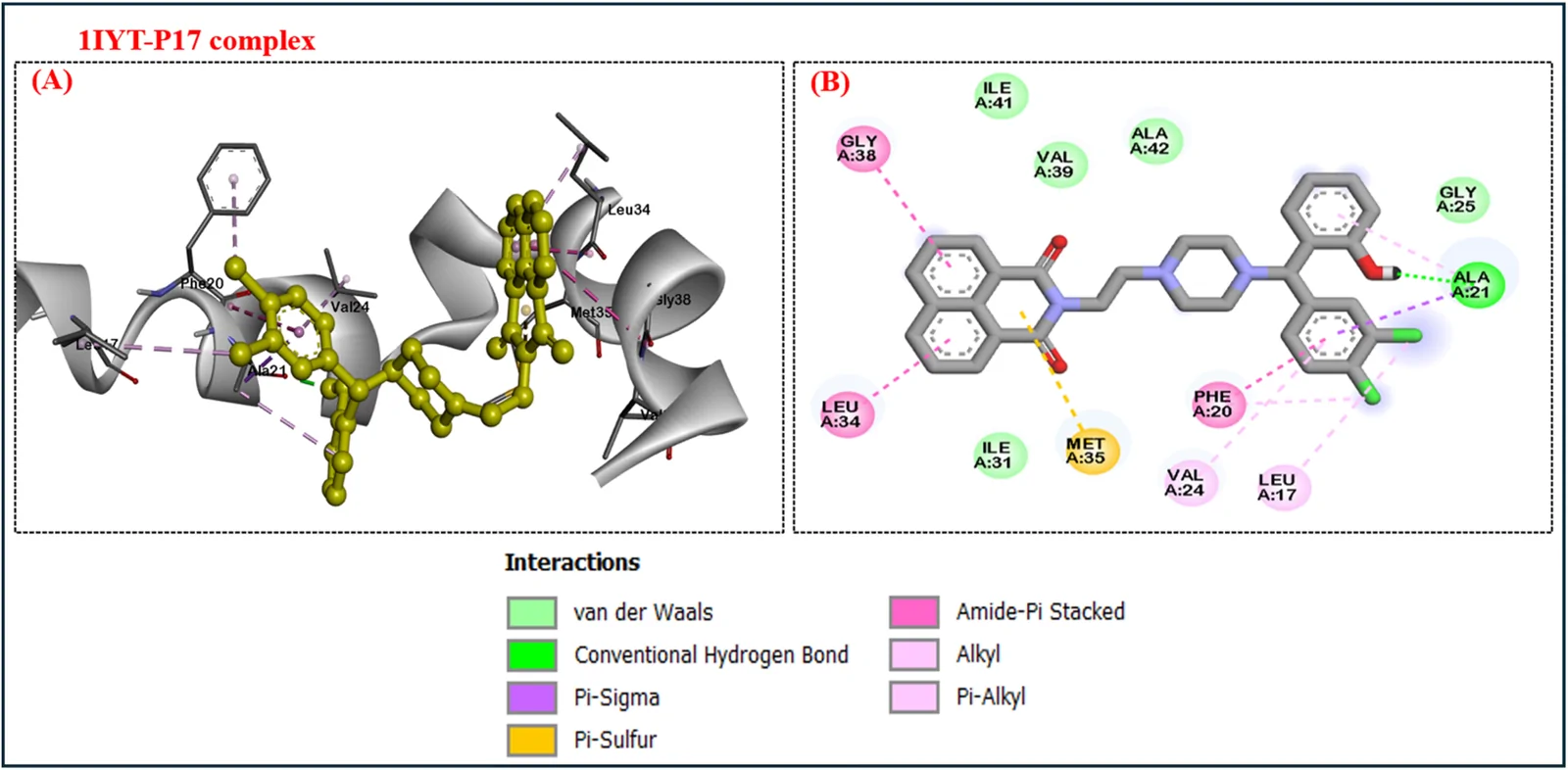
Molecular docking pose 3D (A) and 2D (B) of P17 with protein Aβ (PDB ID:1IYT) surrounded by different amino acid residues represented by several colors.

Collectively, the docking poses of P12 and P17 revealed favorable predicted ligand–Aβ interaction patterns within the monomeric Aβ1-42 structure. However, the docking results alone do not establish complex stability or inhibition of Aβ aggregation. Therefore, the docking analysis indicates that P10, P12, and P17 adopted favorable predicted binding orientations within their respective target structures, with P12 and P17 showing more favorable predicted docking scores than the reference ligand quercetin, while P10 exhibited a favorable predicted docking score toward MAO-B. These findings provide structural insights into the possible ligand-target interaction modes of the selected compounds.

#### 2.3.2. Molecular dynamics simulation studies

The dynamic behaviour of the protein–ligand complexes of MAO-B (PDB ID: 2V61) with ligand P10 and Aβ (PDB ID: 1IYT) with ligands P12 and P17 was investigated using 100 ns molecular dynamics (MD) simulations. For the 2V61-P10 complex, the RMSD profile (Fig. 8A) shows an initial equilibration phase during the first ∼30–40 ns, followed by relatively limited variation of the protein Cα atoms within ∼2.7–3.1 Å. This profile is consistent with comparatively limited conformational rearrangement of the MAO-B structure during the later phase of the simulation. The ligand RMSD increases during the early phase and remains within ∼0.5–1.8 Å thereafter, indicating relatively limited positional displacement of P10 with respect to its reference conformation during the simulation. The RMSF profile analysis (Fig. 8B) further shows that most residues display low fluctuations within ∼0.5–0.9 Å, with a slight increase (∼1.4 Å) toward the terminal region. These relatively low residue-level fluctuations are consistent with limited flexibility of most of the MAO-B residues analyzed.

**Fig. 8:**
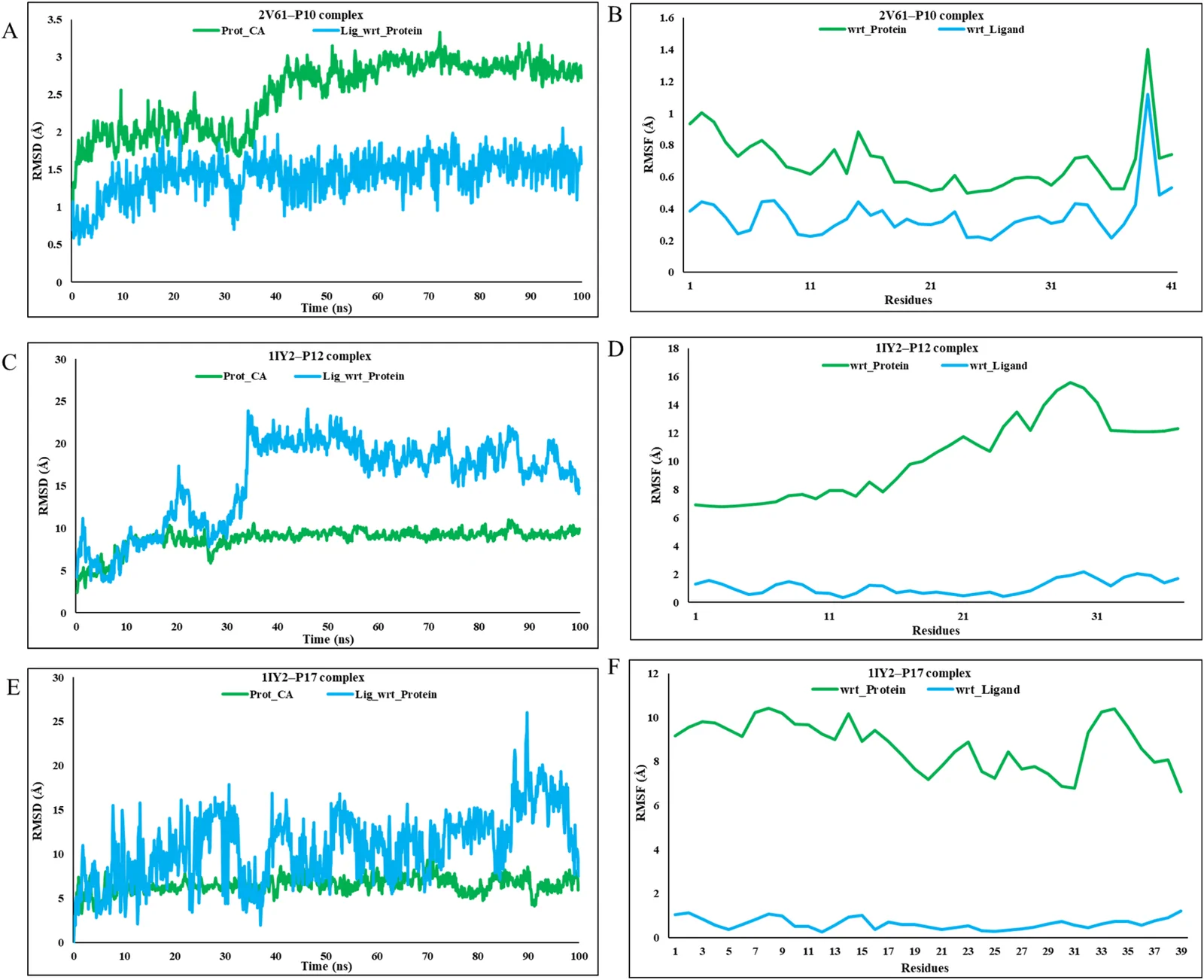
MD-simulation analysis of protein–ligand complexes over 100 ns of simulation time (A) RMSD and (B) RMSF profiles of MAO-B and its complex with P10, (C) RMSD and (D) RMSF profiles of amyloid-β and its complex with P12, (E) RMSD and (F) RMSF profiles of amyloid-β and its complex with P17.

In contrast, the RMSD profile of the 1IYT–P12 complex (Fig. 8C) shows substantially larger conformational fluctuations than those observed for the MAO-B–P10 complex. The protein Cα atoms exhibit RMSD values within ∼8.0–10.5 Å, indicating substantial conformational rearrangement of the monomeric Aβ1-42 structure during the simulation. The ligand RMSD shows substantial positional variation, reaching approximately ∼15–22 Å relative to the reference conformation. These relatively large deviations indicate considerable conformational flexibility of the Aβ1-42 structure and positional rearrangement of P12 during the simulation. Therefore, these RMSD values should not be interpreted as direct evidence of a rigidly stable Aβ-P12 complex. The RMSF profile (Fig. 8D) reveals elevated residue fluctuations ranging from ∼7–16 Å, particularly toward the terminal regions, reflecting the pronounced conformational flexibility of Aβ1-42. The ligand exhibits comparatively lower local fluctuations (∼0.5–2.2 Å). The difference between the ligand RMSD and RMSF reflects the distinct nature of these parameters, as RMSD describes positional displacement relative to a reference structure, whereas RMSF describes local fluctuations around the average position. Similarly, the RMSD profile (Fig. 8E) for the 1IYT–P17 complex shows substantial conformational variation during the 100 ns simulation. The protein Cα atoms exhibit RMSD values within ∼6.0–8.5 Å, indicating considerable conformational rearrangement of the monomeric Aβ1-42 structure. The ligand RMSD fluctuates within ∼5–25 Å, indicating substantial positional rearrangement of P17 relative to its initial reference conformation. Thus, the ligand RMSD profile reflects dynamic positional behavior rather than definitive evidence of stable binding. The RMSF analysis (Fig. 8F) indicates protein residue fluctuations within ∼7–11 Å, consistent with the conformational flexibility of Aβ1-42, while the ligand exhibits comparatively lower local fluctuations (∼0.3–1.2 Å). These RMSF values indicate relatively limited local ligand fluctuations around the sampled conformations but do not independently establish stable binding.

The molecular surface area (MolSA), polar surface area (PSA), radius of gyration (rGyr), and solvent-accessible surface area (SASA) profiles of the 2V61–P10, 1IYT–P12, and 1IYT–P17 complexes were further analyzed to characterize changes in surface properties and conformational behavior throughout the 100 ns simulations (Fig. 9). The MolSA profiles (Fig. 9A) show relatively consistent values for all three complexes throughout the simulation, although the 1IYT–P12 complex exhibits more pronounced transient fluctuations compared with the 2V61–P10 and 1IYT–P17 complexes. The PSA profiles (Fig. 9B) show fluctuations within a relatively comparable range for all three systems, indicating variations in polar surface exposure during the simulations. The rGyr profiles (Fig. 9C) show comparatively limited variation in the overall compactness of the 2V61–P10 complex, whereas the 1IYT–P12 and 1IYT–P17 complexes exhibit greater fluctuations during certain periods of the simulation. These variations are consistent with the greater conformational flexibility observed for the Aβ-containing systems. The SASA profiles (Fig. 9D) show a clear distinction between the MAO-B–P10 and Aβ-containing complexes. The 2V61–P10 complex maintains substantially lower SASA values with relatively limited variation throughout the simulation, whereas the 1IYT–P12 and 1IYT–P17 complexes display considerably higher SASA values and greater fluctuations. These observations indicate increased solvent exposure and conformational rearrangement in the Aβ-containing systems.

**Fig. 9:**
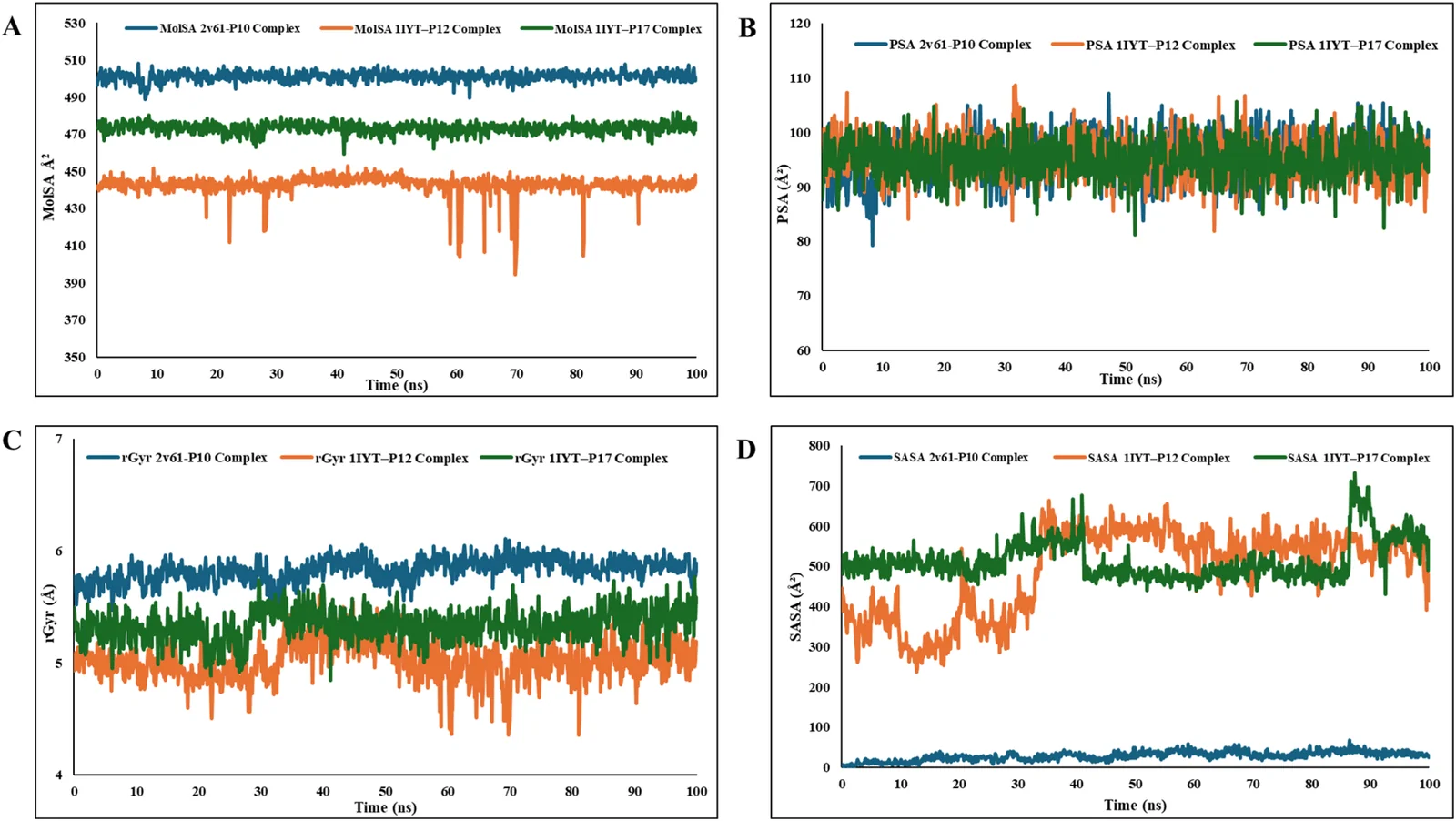
(A) MolSA profiles, (B) PSA analysis, (C) radius of gyration (rGyr), and (D) SASA profiles of the compounds P10 complexed with 2v61 (blue) and P12 and P17 complexed with 1IYT (orange and green) over the course of a 100 ns simulation.

Collectively, the trajectory analyses indicate that the 2V61–P10 complex exhibited comparatively limited structural and positional fluctuations during the 100 ns simulation. In contrast, the 1IYT–P12 and 1IYT–P17 complexes showed substantially greater conformational and positional fluctuations, reflecting the dynamic nature and flexibility of the monomeric Aβ1-42 structure. Therefore, the MD results provide insights into the dynamic behavior and conformational characteristics of the simulated complexes but should not be considered alone as definitive evidence of stable Aβ-ligand binding.

#### 2.3.3. Physicochemical properties

The *in silico* physicochemical properties of the most active compounds (P10, P12, and P17), along with the reference compounds Safinamide and Quercetin, were analyzed using the QikProp tool to calculate predicted physicochemical and ADME-related parameters, and the key parameters are displayed in Tables 3 and 4. The calculated descriptors included molecular weight, hydrogen-bonding properties, lipophilicity, polar surface area, oral absorption, and predicted brain/blood distribution. Compound P12 complied with the evaluated Lipinski's Rule of Five (RO5)^18^ criteria without any violations, whereas P10 and P17 showed two RO5 violations due to their molecular weights (>500 Da) and QPlogPo/w values (>5.0).^19^ Similarly, the reference compounds Safinamide and Quercetin also complied with Lipinski's Rule of Five without any violations. Although P10 and P17 satisfied the evaluated limits for hydrogen bond donors, hydrogen bond acceptors, and rotatable bonds, their two RO5 violations indicate physicochemical liabilities that may require further optimization. Therefore, P10 and P17 should not be considered fully Lipinski-compliant, whereas P12 showed compliance with the evaluated RO5 criteria. Additionally, the significant ADME parameters were predicted for the most active compounds (P10, P12, and P17), and Table 4 presents the findings along with their predicted values. The predicted human oral absorption values were 84.60%, 92.18%, and 87.95% for P10, P12, and P17, respectively, while the predicted QPlogBB values were 0.063, −0.009, and 0.075, respectively. The QPlogBB values provide preliminary computational estimates of brain/blood distribution and should not be interpreted as experimental evidence of blood–brain barrier (BBB) penetration or established CNS exposure. Furthermore, the predicted QPPCaco, QPlogKhsa, QPlogHERG, and QPlogS values provided preliminary estimates of intestinal permeability, serum albumin binding, hERG-related risk, and aqueous solubility, respectively. Although Quercetin exhibited comparatively lower predicted Caco-2 permeability and oral absorption than the synthesized compounds, its values remain within the reported pharmacokinetic profile. Overall, the QikProp analysis provides preliminary computational information that can guide the physicochemical and ADME-related optimization of P10, P12, and P17. Experimental pharmacokinetic studies, BBB permeability assessment, and CNS exposure studies would be required to establish their suitability as CNS-active drug candidates.

**Table 3:** Prediction of Lipinski RO5 for active compounds (P10, P12, and P17), in comparison with safinamide and quercetin

Compound	MW (<500 Da)	HBD (<5)	HBA (<10)	QPlogPo/w (<5)	RO5 violations (<2)	nRB (0–15)
P10	590.50	2	8.50	5.49	2	8
P12	491.58	2	7.75	4.41	0	7
P17	560.48	2	7.75	5.37	2	7
Safinamide	302.35	3	4.75	2.084	0	7
Quercetin	302.24	4	5.25	0.367	0	5

**Table 4:** Prediction of ADME parameters of active compounds P10, P12, and P17

Compound	Human oral absorption (>80%-high& <25%-poor)	QPPCaco (<25 poor; >500 great)	QPlogBB (−3.0 to 1.2)	QPlogKhsa (−1.5 to 1.5)	QPlogHERG (>−5)	QPlogS (−6.5 to 0.5)	PSA (70–200 Å^2^)
P10	84.60	312.47	0.063	0.52	−6.89	−5.12	88.77
P12	92.18	615.81	−0.009	0.41	−6.12	−4.36	76.65
P17	87.95	458.29	0.075	0.49	−6.54	−4.91	82.21
Safinamide	76.00	118.00	−0.443	−0.236	−5.484	−2.087	70.31
Quercetin	52.00	18.00	−2.419	−0.343	−5.109	−2.909	143.33

## 3. Conclusion

To develop effective therapeutics for AD, a multitargeted approach can be effective in evaluating the potential of our compounds. Due to the multifactorial nature of this disease, drugs targeting a single factor have proved ineffective. In this study, a series of naphthalimide-based hybrids (P1–P18) was synthesized and evaluated against key pathological targets, including hAChE, esBuChE, MAO-A, MAO-B, Aβ, and PHF6 aggregation. The compounds predominantly exhibited selective MAO-B inhibition along with potent Aβ aggregation inhibitory activity. In the series, P10 showed remarkable inhibition of MAO-B with an IC_50_ value of 3.09 ± 0.10 µM. P12 and P17 were the most potent inhibitors of Aβ, showing 83% and 91% inhibition, respectively. *In vitro* cytotoxicity assay of the three lead compounds was conducted using SH-SY5Y cell lines, and all compounds were found to be non-toxic even at concentrations up to 25 µM. Furthermore, these compounds also exhibited neuroprotective effects against Aβ and H_2_O_2_-induced neurotoxicity in SH-SY5Y cells. Notably, P10 was most effective in reducing H_2_O_2_ induced oxidative stress, while P12 emerged as the most promising candidate for mitigating Aβ-induced neurotoxicity within this series. Molecular docking studies provided structural insights into the predicted interaction modes of P10 with MAO-B and P12 and P17 with the monomeric Aβ1-42 structure. Molecular dynamics simulations over 100 ns further characterized the conformational and positional behavior of these complexes, with comparatively limited fluctuations observed for the MAO-B–P10 system and greater conformational flexibility observed in the Aβ-containing systems. Physicochemical and ADME evaluation provided preliminary computational information regarding the predicted drug-related properties of the selected compounds, including oral absorption and brain/blood distribution. These computational predictions require further experimental validation, particularly for pharmacokinetic properties and BBB penetration. Accordingly, these dual-acting compounds represent promising candidates for further development of new MTDLs as potential therapeutics for Alzheimer's disease.

## 4. Experimental section

### 4.1. Chemistry

All chemicals and solvents used in the synthesis and characterization of the target compounds were procured from certified commercial suppliers, including Sigma-Aldrich, BLD Pharmatech Pvt. Ltd, and Spectrochem Pvt. Ltd (India), and were utilized without further purification unless otherwise specified. Solvents were dried following standard procedures before use. The course of each reaction was periodically assessed by thin-layer chromatography (TLC) using aluminum precoated silica gel 60 F_254_ Merck TLC plates. UV light with wavelengths of 254 nm and 365 nm was used to visualize the spots. The synthesized compounds were purified by column chromatography employing silica gel (mesh size 200–400) as the stationary phase and appropriate solvent systems as the mobile phase. Melting points of solid compounds were determined on a Tanco PLT-276 apparatus using the open capillary method and are reported as uncorrected. Using a Bruker Avance spectrometer, ^1^H NMR and ^13^C NMR spectra were obtained. Chemical shifts (*δ*) are expressed in parts per million (ppm) using tetramethylsilane (TMS) as the internal reference. Coupling constants (*J*) are reported in hertz (Hz). Signal patterns are designated as singlet (s), doublet (d), doublet of doublets (dd), triplet (t), quartet (q), and multiplet (m). Spectral analysis was performed using MestreNova (version 6.0.2). High-resolution mass spectra (HRMS) and accurate mass measurements were obtained using an Agilent G6530AA Q-TOF (LC-HRMS) instrument.

#### 4.1.1. General procedure for the synthesis of 2-(2-(piperazin-1-yl)ethyl)-1*H*-benzo[de]isoquinoline-1,3(2*H*)-dione (3)

In an oven-dried 100 mL round-bottom flask, 1,8-naphthalic anhydride (3.96 g, 20 mmol) and triethylamine (2.8 mL, 20 mmol) were dissolved in anhydrous toluene (20 mL). *N*-(2-Aminoethyl) piperazine (2.62 mL, 20 mmol) was added dropwise to the stirred solution maintained at 60 °C. The resulting mixture was refluxed for 12 h at 100–110 °C under a nitrogen atmosphere, and its progress was tracked by thin-layer chromatography (TLC). After completion, the mixture was allowed to cool to ambient temperature, and the solvent (toluene) was evaporated under reduced pressure. The resulting crude material was washed three times with hexane and diethyl ether, then air-dried to yield a creamy solid. Further purification was performed using silica gel column chromatography with a methanol/chloroform (10 : 90) eluent, yielding compound 3 as an off-white solid in 83% yield. ^1^H NMR (400 MHz, chloroform-*d*) *δ* 8.54 (d, *J* = 7.2 Hz, 2H), 8.17 (d, *J* = 8.3 Hz, 2H), 7.71 (t, *J* = 7.8 Hz, 2H), 4.31 (t, 2H), 2.88–2.81 (m, 4H), 2.66 (t, 2H), 2.56 (brs, 4H). ^13^C NMR (101 MHz, chloroform-*d*) *δ*: 164.23, 133.98, 131.65, 131.25, 127.81, 125.93, 122.73, 56.73, 54.68, 46.95, 38.26.

#### 4.1.2. General procedure for the synthesis of 2-(2-(4-((substituted)methyl)piperazin-1-yl)ethyl)-1*H*-benzo[de]isoquinoline-1,3(2*H*)-dione (P1–P18)

To a stirred solution of intermediate 3 (1 mmol) in dry DMF, salicylaldehyde/*o*-vanillin (1 mmol) was added dropwise at room temperature. Subsequently, the substituted boronic acid (1.5 mmol) was added, and the reaction mixture was refluxed at 90 °C under an inert atmosphere for 8–10 hours. The reaction progress was periodically monitored by thin-layer chromatography (TLC). After confirming completion by TLC, the reaction mixture was cooled to room temperature and slowly poured into ice-cold water, resulting in the precipitation of the targeted compounds (P1–P18). The resultant solid was filtered, washed repeatedly with water, and allowed to air dry. Purification of the crude product was achieved by column chromatography using EtOAc/pet ether (20 : 80) as the eluent, affording the pure compounds (P1–P18) in yields of 70–80%.

##### 4.1.2.1. 2-(2-(4-((4-Fluorophenyl)(2-hydroxy-3-methoxyphenyl)methyl)piperazin-1-yl)ethyl)-1*H*-benzo[de]isoquinoline-1,3(2*H*)-dione (P1)

White powder; yield 73%; mp 186–188 °C. ^1^H NMR (400 MHz, chloroform-*d*) *δ* 12.18 (s, 1H), 8.56 (d, *J* = 7.3 Hz, 2H), 8.19 (d, *J* = 8.2 Hz, 2H), 7.73 (t, *J* = 7.8 Hz, 2H), 7.38–7.35 (m, 2H), 6.95 (t, *J* = 8.4 Hz, 2H), 6.73 (d, *J* = 7.9 Hz, 1H), 6.65 (t, *J* = 7.9 Hz, 1H), 6.50 (d, *J* = 7.7 Hz, 1H), 4.39 (s, 1H), 4.30 (t, *J* = 7.1 Hz, 2H), 3.86 (s, 3H), 2.76–2.44 (m, 10H). ^13^C NMR (101 MHz, chloroform-*d*) *δ* 164.25, 162.43(d, *J*_CF_ = 246.8 Hz), 149.64, 148.45, 145.76, 135.61, 134.05, 131.67, 131.31, 130.28(d, *J*_CF_ = 8.8 Hz), 128.26, 127.03, 125.48, 122.70, 120.95, 119.13, 115.76(d, *J*_CF_ = 20.7Hz), 110.44, 75.24, 55.89, 55.53, 53.36 (2C), 37.47. HRMS (ESI) for C_32_H_31_FN_3_O_4_: observed [M + H]^+^: 540.2263; calculated [M + H]^+^: 540.2299.

##### 4.1.2.2. 2-(2-(4-((2-Hydroxy-3-methoxyphenyl)(phenyl)methyl)piperazin-1-yl)ethyl)-1*H*-benzo [de]isoquinoline-1,3(2*H*)-dione (P2)

White solid; yield 78%; mp 190–192 °C.^1^H NMR (400 MHz, chloroform-*d*) *δ* 12.31 (s, 1H), 8.56 (dd, *J* = 7.3, 1.2 Hz, 2H), 8.19 (dd, *J* = 8.4, 1.2 Hz, 2H), 7.73 (t, *J* = 8.3 Hz, 2H), 7.39 (d, *J* = 7.4 Hz, 2H), 7.28 (d, *J* = 1.9 Hz, 1H), 7.24–7.19 (m, 2H), 6.72 (dd, *J* = 8.1, 1.5 Hz, 1H), 6.64 (t, *J* = 7.9 Hz, 1H), 6.53 (dd, *J* = 7.7, 1.5 Hz, 1H), 4.39 (s, 1H), 4.29 (t, *J* = 6.2 Hz, 2H), 3.86 (s, 3H), 2.76–2.46 (m, 10H). ^13^C NMR (101 MHz, chloroform-*d*) *δ* 164.26, 148.39, 145.94, 139.74, 134.05, 131.68, 131.32, 128.90, 128.63, 128.27, 128.04, 127.03, 125.63, 122.71, 121.14, 118.99, 110.35, 76.17, 55.90, 55.57, 53.43 (2C), 37.50. HRMS (ESI) for C_32_H_32_N_3_O_4_: observed [M + H]^+^: 522.2365; calculated [M + H]^+^: 522.2393.

##### 4.1.2.3. 2-(2-(4-((3-Chloro-4-fluorophenyl)(2-hydroxy-3-methoxyphenyl)methyl)piperazin-1-yl) ethyl)-1*H*-benzo[de]isoquinoline-1,3(2*H*)-dione (P3)

Pale yellow solid; yield 70%; mp 152–154 °C. ^1^H NMR (400 MHz, chloroform-*d*) *δ* 11.87 (s, 1H), 8.56 (d, *J* = 6.1 Hz, 2H), 8.19 (d, *J* = 7.1 Hz, 2H), 7.73 (t, *J* = 7.8 Hz, 2H), 7.43–7.41 (m, 1H), 7.32–7.28 (m, 1H), 7.03 (t, *J* = 8.6 Hz, 1H), 6.75–6.73 (m, 1H), 6.67 (t, *J* = 7.9 Hz, 1H), 6.51–6.49 (m, 1H), 4.34 (s, 1H), 4.30 (t, *J* = 6.5 Hz, 2H), 3.86 (s, 3H), 2.76–2.43 (m, 10H). ^13^C NMR (101 MHz, chloroform-*d*) *δ* 164.26, 157.72 (d, *J*_CF_ = 249.7 Hz), 148.50, 145.60, 137.05 (d, *J*_CF_ = 3.4 Hz), 134.06, 131.67, 131.31, 130.62, 128.26, 127.03, 124.88, 122.69, 120.81, 119.32, 117.06 (d, *J*_CF_ = 20.7 Hz), 110.67, 74.82, 55.93, 55.52, 53.31 (2C), 37.47. HRMS (ESI) for C_32_H_30_ClFN_3_O_4_: observed [M + H]^+^: 574.1914; calculated [M + H]^+^: 574.1909.

##### 4.1.2.4. 2-(2-(4-((3,4-Difluorophenyl)(2-hydroxy-3-methoxyphenyl)methyl)piperazin-1-yl)ethyl)-1*H*-benzo[de]isoquinoline-1,3(2*H*)-dione (P4)

Pale yellow solid; yield 80%; mp 222–224 °C.^1^H NMR (400 MHz, chloroform-*d*) *δ* 11.93 (s, 1H), 8.57 (d, *J* = 7.3 Hz, 2H), 8.20 (d, *J* = 8.2 Hz, 2H), 7.74 (t, *J* = 7.8 Hz, 2H), 7.30–7.25 (m, 1H), 7.16–7.13 (m, 1H), 7.09–7.03 (m, 1H), 6.76 (dd, *J* = 8.1, 1.5 Hz, 1H), 6.69 (t, *J* = 7.9 Hz, 1H), 6.52 (dd, *J* = 7.8, 1.5 Hz, 1H), 4.36 (s, 1H), 4.31 (t, *J* = 6.9 Hz, 2H), 3.88 (s, 3H), 2.82–2.38 (m, 10H). ^13^C NMR (101 MHz, chloroform-*d*) *δ* 164.17 (2C), 150.31 (dd, *J*_CF_ = 249.2, 13.1 Hz), 149.95 (dd, *J*_CF_ = 249.1, 12.6 Hz), 148.43 (2C), 145.51 (2C), 136.89 (t, *J*_CF_ = 4.5 Hz), 133.97 (2C), 131.59, 131.22 (2C), 128.17, 126.95 (2C), 124.92, 124.56, 122.61, 120.74, 119.26, 117.42 (t, *J*_CF_ = 17.6 Hz), 110.61, 74.88, 55.86 (2C), 55.46, 53.25 (2C), 51.63, 37.40. HRMS (ESI) for C_32_H_30_F_2_N_3_O_4_: observed [M + H]^+^: 558.2187; calculated [M + H]^+^: 558.2204.

##### 4.1.2.5. 2-(2-(4-((2-Hydroxy-3-methoxyphenyl)(*p*-tolyl)methyl)piperazin-1-yl)ethyl)-1*H*-benzo [de]isoquinoline-1,3(2*H*)-dione (P5)

White solid; yield 75%; mp 184–186 °C.^1^H NMR (400 MHz, chloroform-*d*) *δ* 12.39 (s, 1H), 8.56 (dd, *J* = 7.3, 1.1 Hz, 2H), 8.19 (dd, *J* = 8.3, 1.2 Hz, 2H), 7.75–7.71 (m, 1H), 7.27 (d, *J* = 7.7 Hz, 1H), 7.06 (d, *J* = 8.0 Hz, 2H), 6.71 (dd, *J* = 8.1, 1.5 Hz, 1H), 6.63 (t, *J* = 7.9 Hz, 1H), 6.51 (dd, *J* = 7.8, 1.5 Hz, 1H), 4.36 (s, 1H), 4.29 (t, *J* = 6.9 Hz, 2H), 3.86 (s, 3H), 2.73–2.27 (m, 10H), 2.27 (s, 3H). ^13^C NMR (101 MHz, chloroform-*d*) *δ* 164.26, 148.36, 145.92, 137.77, 136.72, 134.05, 131.67, 131.32, 129.56, 128.54, 128.27, 127.03, 125.80, 122.71, 121.10, 118.97, 110.24, 75.85, 55.89, 55.56, 53.44 (2C), 37.50, 21.17. HRMS (ESI) for C_33_H_34_N_3_O_4_: observed [M + H]^+^: 536.2536; calculated [M + H]^+^: 536.2549.

##### 4.1.2.6. 2-(2-(4-((4-Chlorophenyl)(2-hydroxy-3-methoxyphenyl)methyl)piperazin-1-yl)ethyl)- 1*H*-benzo[de]isoquinoline-1,3(2*H*)-dione (P6)

White solid; yield 78%; mp 202–204 °C.^1^H NMR (400 MHz, chloroform-*d*) *δ* 12.10 (s, 1H), 8.55 (dd, *J* = 7.3, 1.2 Hz, 2H), 8.19 (dd, *J* = 8.3, 1.2 Hz, 2H), 7.75–7.70 (m, 2H), 7.33 (d, *J* = 8.2 Hz, 2H), 7.25–7.21 (m, 2H), 6.73 (dd, *J* = 8.1, 1.5 Hz, 1H), 6.65 (t, *J* = 7.9 Hz, 1H), 6.49 (dd, *J* = 7.8, 1.5 Hz, 1H), 4.37 (s, 1H), 4.29 (t, *J* = 6.9 Hz, 2H), 3.86 (s, 3H), 2.84–2.39 (m, 10H). ^13^C NMR (101 MHz, chloroform-*d*) *δ* 164.26, 148.46, 145.74, 138.33, 134.06, 133.85, 131.68, 131.32, 129.94, 129.09, 128.27, 127.04, 125.22, 122.71, 120.91, 119.20, 110.53, 75.33, 55.91, 55.54, 53.36 (2C), 37.49. HRMS (ESI) for C_32_H_31_ClN_3_O_4_: observed [M + H]^+^: 556.1992; calculated [M + H]^+^: 556.2003.

##### 4.1.2.7. 2-(2-(4-((2-Hydroxy-3-methoxyphenyl)(4-methoxyphenyl)methyl)piperazin-1-yl)ethyl)-1*H*-benzo[de]isoquinoline-1,3(2*H*)-dione (P7)

Pale yellow solid; yield 68%; mp 98–100 °C.^1^H NMR (400 MHz, chloroform-*d*) *δ* 12.39 (s, 1H), 8.56 (d, *J* = 7.3 Hz, 2H), 8.19 (d, *J* = 8.4 Hz, 2H), 7.76–7.70 (m, 2H), 7.29 (d, *J* = 8.1 Hz, 2H), 6.78 (d, *J* = 9.0 Hz, 2H), 6.72 (dd, *J* = 8.1, 1.6 Hz, 1H), 6.64 (t, *J* = 7.9 Hz, 1H), 6.51 (dd, *J* = 7.7, 1.6 Hz, 1H), 4.36 (s, 1H), 4.29 (t, *J* = 6.9 Hz, 2H), 3.86 (s, 3H), 3.74 (s, 3H), 2.81–2.37 (m, 10H). ^13^C NMR (101 MHz, chloroform-*d*) *δ* 164.26, 159.30, 148.39, 145.92, 134.05, 131.77, 131.68, 131.32, 129.87, 128.27, 127.03, 125.91, 122.71, 121.09, 118.96, 114.16, 110.25, 74.79, 55.89, 55.56, 55.31, 53.44 (2C), 37.50. HRMS (ESI) for C_33_H_34_N_3_O_5_: observed [M + H]^+^: 552.2491; calculated [M + H]^+^: 552.2498.

##### 4.1.2.8. 2-(2-(4-((2-Hydroxy-3-methoxyphenyl)(4-(trifluoromethyl)phenyl)methyl)piperazin-1-yl) ethyl)-1*H*-benzo[de]isoquinoline-1,3(2*H*)-dione (P8)

Pale yellow solid; yield 70%; mp 84–86 °C.^1^H NMR (400 MHz, chloroform-*d*) *δ* 11.90 (s, 1H), 8.57 (d, *J* = 7.3 Hz, 2H), 8.21 (d, *J* = 8.3 Hz, 2H), 7.81–7.69 (m, 2H), 7.59–7.50 (m, 4H), 6.76 (d, *J* = 8.1 Hz, 1H), 6.68 (t, *J* = 7.9 Hz, 1H), 6.54 (d, *J* = 7.7 Hz, 1H), 4.47 (s, 1H), 4.32 ((t, *J* = 6.9 Hz, 2H), 3.88 (s, 3H), 2.88–2.33 (m, 10H). ^19^F NMR (376 MHz, chloroform-*d*) *δ* −62.63 (s, 3H). ^13^C NMR (101 MHz, CDCl_3_) *δ* 164.19, 148.42, 145.59, 143.87, 135.09, 133.98, 131.61, 131.23, 130.12(d, *J*_CF_ = 32.32 Hz), 128.75, 128.19, 126.95, 125.85(d, *J*_CF_ = 4.04 Hz), 124.80, 122.63, 120.78, 119.28, 110.65, 77.37, 77.05, 76.73, 75.41, 55.85, 55.47, 53.24, 37.36. HRMS (ESI) for C_33_H_31_F_3_N_3_O_4_: observed [M + H]^+^: 590.2259; calculated [M + H]^+^: 590.2267.

##### 4.1.2.9. 2-(2-(4-((2-Hydroxy-3-methoxyphenyl)(4-(trifluoromethoxy)phenyl)methyl)piperazin-1-yl)ethyl)-1*H*-benzo[de]isoquinoline-1,3(2*H*)-dione (P9)

Pale yellow solid; yield 72%; mp 86–88 °C.^1^H NMR (400 MHz, chloroform-*d*) *δ* 12.06 (s, 1H), 8.58 (d, *J* = 7.5 Hz, 2H), 8.21 (d, *J* = 8.4 Hz, 2H), 7.75 (t, *J* = 7.8 Hz, 2H), 7.45 (d, *J* = 8.3 Hz, 2H), 7.12 (d, *J* = 8.7 Hz, 2H), 6.76 (d, *J* = 8.1 Hz, 1H), 6.68 (t, *J* = 7.9 Hz, 1H), 6.53 (d, *J* = 7.8 Hz, 1H), 4.42 (s, 1H), 4.32 (t, *J* = 6.9 Hz, 2H), 3.88 (s, 3H), 2.82–2.41 (m, 10H). ^13^C NMR (101 MHz, chloroform-*d*) *δ* 164.19, 148.79, 148.42, 145.65, 138.45, 135.43, 133.97, 131.61, 131.24, 129.93, 128.20, 126.95, 125.14, 122.64, 121.20, 120.86, 119.16, 110.54, 75.14, 55.85, 55.49, 53.28, 37.40. HRMS (ESI) for C_33_H_31_F_3_N_3_O_5_: observed [M + H]^+^: 606.2209; calculated [M + H]^+^: 606.2216.

##### 4.1.2.10. 2-(2-(4-((3,4-Dichlorophenyl)(2-hydroxy-3-methoxyphenyl)methyl)piperazin-1-yl)ethyl) -1*H*-benzo[de]isoquinoline-1,3(2*H*)-dione (P10)

Off-white solid; yield 78%; mp 208–210 °C.^1^H NMR (400 MHz, chloroform-*d*) *δ* 11.78 (s, 1H), 8.57 (dd, *J* = 7.3, 1.2 Hz, 2H), 8.20 (dd, *J* = 8.3, 1.2 Hz, 2H), 7.76–7.73 (m, 2H), 7.48 (d, *J* = 1.9 Hz, 1H), 7.36–7.28 (m, 2H), 6.76 (dd, *J* = 8.1, 1.6 Hz, 1H), 6.69 (t, *J* = 7.9 Hz, 1H), 6.52 (dd, *J* = 7.7, 1.5 Hz, 1H), 4.36 (s, 1H), 4.31 (t, *J* = 6.8 Hz, 2H), 3.88 (s, 3H), 2.85–2.39 (m, 10H).^13^C NMR (101 MHz, chloroform-*d*) *δ* 164.19, 148.42, 145.53, 140.12, 133.97, 132.72, 132.01, 131.59, 131.23, 130.92, 130.32, 128.18, 127.67, 126.95, 124.58, 122.62, 120.71, 119.31, 110.67, 74.74, 55.87, 55.47, 53.23, 37.38. HRMS (ESI) for C_32_H_30_Cl_2_N_3_O_4_: observed [M + H]^+^: 590.1604; calculated [M + H]^+^: 590.1613.

##### 4.1.2.11. 2-(2-(4-((4-Fluorophenyl)(2-hydroxyphenyl)methyl)piperazin-1-yl)ethyl)-1*H*-benzo[de] isoquinoline-1,3(2*H*)-dione (P11)

White solid; yield 72%; mp 210–212 °C.^1^H NMR (400 MHz, chloroform-*d*) *δ* 11.94 (s, 1H), 8.57 (d, *J* = 7.2 Hz, 2H), 8.20 (d, *J* = 7.6 Hz, 2H), 7.78–7.72 (m, 2H), 7.38 (m, 2H), 7.12 (ddd, *J* = 8.9, 7.4, 1.7 Hz, 1H), 7.00–6.95 (m, 2H), 6.89–6.85 (m, 2H), 6.71 (td, *J* = 7.4, 1.2 Hz, 1H), 4.40 (s, 1H), 4.32 (t, *J* = 6.9 Hz, 2H), 2.89–2.36 (m, 10H). ^13^C NMR (101 MHz, chloroform-*d*) *δ* 163.10, 161.32(d, *J*_CF_ = 247.45 Hz), 155.25, 134.18(d, *J*_CF_ = 3.03 Hz), 132.92, 130.50, 130.15, 129.23(d, *J*_CF_ = 6.06 Hz), 128.07, 127.68, 127.07, 125.87, 123.89, 121.49, 118.40, 116.15, 114.67(d, *J*_CF_ = 21.21 Hz), 74.30, 54.36, 52.09, 36.29. HRMS (ESI) for C_31_H_29_FN_3_O_3_: observed [M + H]^+^: 510.2177; calculated [M + H]^+^: 510.2193.

##### 4.1.2.12. 2-(2-(4-((2-Hydroxyphenyl)(phenyl)methyl)piperazin-1-yl)ethyl)-1*H*-benzo[de] isoquinoline-1,3(2*H*)-dione (P12)

White solid; yield 74%; mp 176–178 °C.^1^H NMR (400 MHz, chloroform-*d*) *δ* 12.03 (s, 1H), 8.56 (dd, *J* = 7.3, 1.2 Hz, 2H), 8.18 (dd, *J* = 8.2, 1.2 Hz, 2H), 7.75–7.71 (m, 2H), 7.38 (d, *J* = 7.1 Hz, 2H), 7.29–7.23 (m, 3H), 7.09 (ddd, *J* = 8.7, 7.4, 1.7 Hz, 1H), 6.89 (dd, *J* = 7.7, 1.7 Hz, 1H), 6.83 (dd, *J* = 8.1, 1.2 Hz, 1H), 6.68 (td, *J* = 7.4, 1.3 Hz, 1H), 4.40 (s, 1H), 4.30 (t, *J* = 7.8 Hz, 2H), 2.81–2.33 (m, 10H). ^13^C NMR (101 MHz, chloroform-*d*) *δ* 164.27, 156.57, 139.72, 134.05, 131.68, 131.32, 129.32, 128.91, 128.58, 128.27, 128.03, 127.92, 127.03, 125.36, 122.71, 119.40, 117.05, 76.31, 55.57, 53.41, 37. HRMS (ESI) for C_31_H_30_N_3_O_3_: observed [M + H]^+^: 492.2275; calculated [M + H]^+^: 492.2287.

##### 4.1.2.13. 2-(2-(4-((3-Chloro-4-fluorophenyl)(2-hydroxyphenyl)methyl)piperazin-1-yl)ethyl)-1*H*-benzo[de]isoquinoline-1,3(2*H*)-dione (P13)

Pale yellow solid; yield 74%; mp 184–186 °C.^1^H NMR (400 MHz, chloroform-*d*) *δ* 11.72 (s, 1H), 8.56 (dd, *J* = 7.3, 1.2 Hz, 2H), 8.19 (dd, *J* = 8.3, 1.1 Hz, 2H), 7.75–7.71 (m, 2H), 7.43 (d, *J* = 6.8 Hz, 1H), 7.28 (d, *J* = 7.9 Hz, 1H), 7.12 (ddd, *J* = 8.9, 7.3, 1.6 Hz, 1H), 7.04 (t, *J* = 8.7 Hz, 1H), 6.85 (ddd, *J* = 8.0, 5.4, 1.4 Hz, 2H), 6.70 (td, *J* = 7.4, 1.2 Hz, 1H), 4.33 (s, 1H), 4.32–4.28 (m, 2H), 2.78–2.36 (m, 10H). ^13^C NMR (101 MHz, chloroform-*d*) *δ* 164.27, 157.73(d, *J*_CF_ = 250.48 Hz), 156.27, 136.99(d, *J*_CF_ = 4.04 Hz),134.06, 131.68, 131.31, 130.67, 129.09, 128.99, 128.26, 127.03, 124.58, 122.69, 119.65, 117.37, 117.02(d, *J*_CF_ = 23.23 Hz), 75.19, 55.50, 53.27, 37.48. HRMS (ESI) for C_31_H_28_ClFN_3_O_3_: observed [M + H]^+^: 544.1770; calculated [M + H]^+^: 544.1803.

##### 4.1.2.14. 2-(2-(4-((2-Hydroxyphenyl)(*p*-tolyl)methyl)piperazin-1-yl)ethyl)-1*H*-benzo[de] isoquinoline-1,3(2*H*)-dione (P14)

White solid; yield 72%; mp 170–172 °C.^1^H NMR (400 MHz, chloroform-*d*) *δ* 12.07 (s, 1H), 8.56 (dd, *J* = 7.2, 1.1 Hz, 2H), 8.18 (dd, *J* = 8.3, 1.2 Hz, 2H), 7.75–7.71 (m, 2H), 7.27–7.25 (m, 2H), 7.10–7.05 (m, 3H), 6.88 (dd, *J* = 7.6, 1.7 Hz, 1H), 6.83 (dd, *J* = 8.1, 1.2 Hz, 1H), 6.67 (td, *J* = 7.4, 1.3 Hz, 1H), 4.37 (s, 1H), 4.30 (t, *J* = 7.2 Hz, 2H), 2.82–2.32 (m, 10H), 2.28 (s, 3H). ^13^C NMR (101 MHz, chloroform-*d*) *δ* 164.26, 156.58, 137.73, 136.69, 134.05, 131.68, 131.32, 129.56, 129.27, 128.47, 128.27, 127.03, 125.55, 122.71, 119.38, 116.98, 75.95, 55.56, 53.44, 37.53, 21.16. HRMS (ESI) for C_32_H_32_N_3_O_3_: observed [M + H]^+^: 506.2422; calculated [M + H]^+^: 506.2444.

##### 4.1.2.15. 2-(2-(4-((4-Chlorophenyl)(2-hydroxyphenyl)methyl)piperazin-1-yl)ethyl)-1*H*-benzo [de]isoquinoline-1,3(2*H*)-dione (P15)

White solid; yield 78%; mp 182–184 °C.^1^H NMR (400 MHz, chloroform-*d*) *δ* 11.87 (s, 1H), 8.56 (d, *J* = 7.3 Hz, 2H), 8.19 (d, *J* = 8.3 Hz, 2H), 7.73 (t, *J* = 7.8 Hz, 2H), 7.32 (d, *J* = 7.8 Hz, 2H), 7.26–7.22 (m, 2H), 7.10 (t, *J* = 7.7 Hz, 1H), 6.85 (td, *J* = 9.2, 8.0, 2.6 Hz, 2H), 6.69 (t, *J* = 7.4 Hz, 1H), 4.36 (s, 1H), 4.30 (t, *J* = 7.0 Hz, 2H), 2.82–2.37 (m, 10H). ^13^C NMR (101 MHz, chloroform-*d*) *δ* 164.28, 156.41, 138.28, 134.07, 133.85, 131.69, 131.33, 129.97, 129.16, 129.10, 128.82, 128.27, 127.05, 124.92, 122.70, 119.56, 117.21, 75.55, 55.52, 53.34, 37.51. HRMS (ESI) for C_31_H_29_ClN_3_O_3_: observed [M + H]^+^: 526.1885; calculated [M + H]^+^: 526.1897.

##### 4.1.2.16. 2-(2-(4-((2-Hydroxyphenyl)(4-methoxyphenyl)methyl)piperazin-1-yl)ethyl)-1*H*-benzo [de]isoquinoline-1,3(2*H*)-dione (P16)

White solid; yield 70%; mp 204–206 °C.^1^H NMR (400 MHz, chloroform-*d*) *δ* 12.06 (s, 1H), 8.56 (d, *J* = 7.4 Hz, 2H), 8.19 (d, *J* = 8.3 Hz, 2H), 7.73 (t, *J* = 7.8 Hz, 2H), 7.27 (dd, *J* = 13.8, 6.0 Hz, 3H), 7.09 (t, *J* = 7.8 Hz, 1H), 6.88–6.79 (m, 4H), 6.68 (t, *J* = 7.6 Hz, 1H), 4.36 (s, 1H), 4.30 (m, 2H), 3.75 (s, 3H), 2.85–2.37 (m, 10H). ^13^C NMR (101 MHz, chloroform-*d*) *δ* 164.26, 159.29, 156.58, 134.05, 131.69, 131.32, 129.87, 129.26, 128.47, 128.28, 127.04, 125.63, 122.72, 119.38, 117.00, 114.17, 75.47, 55.55, 55.31, 53.43, 37.48. HRMS (ESI) for C_32_H_32_N_3_O_4_: observed [M + H]^+^: 522.2380; calculated [M + H]^+^: 522.2393.

##### 4.1.2.17. 2-(2-(4-((3,4-Dichlorophenyl)(2-hydroxyphenyl)methyl)piperazin-1-yl)ethyl)-1*H*-benzo [de]isoquinoline-1,3(2*H*)-dione (P17)

White solid; yield 80%; mp 174–176 °C. ^1^H NMR (400 MHz, chloroform-*d*) *δ* 11.65 (s, 1H), 8.57 (d, *J* = 7.3 Hz, 2H), 8.20 (d, *J* = 8.3 Hz, 2H), 7.77–7.71 (m, 2H), 7.49 (s, 1H), 7.36 (d, *J* = 8.3 Hz, 1H), 7.28 (d, *J* = 13.9 Hz, 1H), 7.13 (t, *J* = 6.9 Hz, 1H), 6.87 (d, *J* = 8.0 Hz, 2H), 6.72 (t, *J* = 7.4 Hz, 1H), 4.35–4.29 (m, 3H), 2.95–2.33 (m, 10H). ^13^C NMR (101 MHz, chloroform-*d*) *δ* 164.22, 156.13, 139.80, 134.03, 132.84, 132.17, 131.60, 131.26, 130.96, 130.35, 129.09, 129.02, 128.18, 127.73, 126.97, 124.10, 122.58, 119.21, 117.40, 75.22, 55.42, 53.08, 37.35. HRMS (ESI) for C_31_H_28_Cl_2_N_3_O_3_: observed [M + H]^+^: 560.1494; calculated [M + H]^+^: 560.1508.

##### 4.1.2.18. 2-(2-(4-(Benzo[*b*]thiophen-3-yl(2-hydroxy-3-methoxyphenyl)methyl)piperazin-1-yl) ethyl)-1*H*-benzo[de]isoquinoline-1,3(2*H*)-dione (P18)

Light pink solid; yield 76%; mp 210–212 °C. ^1^H NMR (400 MHz, chloroform-*d*) *δ* 11.56 (s, 1H), 8.56 (dd, *J* = 7.3, 1.2 Hz, 2H), 8.18 (dd, *J* = 8.3, 1.2 Hz, 2H), 7.76–7.63 (m, 4H), 7.33–7.22 (m, 3H), 6.77 (dd, *J* = 7.9, 1.7 Hz, 1H), 6.69 (t, *J* = 7.8 Hz, 1H), 6.64 (dd, *J* = 7.8, 1.7 Hz, 1H), 4.78 (s, 1H), 4.31 (t, *J* = 6.9 Hz, 2H), 3.88 (s, 3H), 2.75–2.67 (m, 10H). ^13^C NMR (101 MHz, chloroform-*d*) *δ* 164.14, 148.33, 145.63, 143.44, 140.13, 139.12, 133.88, 131.61, 131.18, 128.20, 126.92, 124.89, 124.40, 124.28, 123.46, 123.18, 122.69, 122.32, 120.86, 119.11, 111.01, 70.77, 55.92, 55.47, 53.31, 37.46. HRMS (ESI) for C_34_H_32_N_3_O_4_S: observed [M + H]^+^: 578.2113; calculated [M + H]^+^: 578.2114.

### 4.2. Biological assays

All enzymes and chemicals were obtained from Merck (Milan, Italy), unless otherwise stated. GenicBio (Shanghai, China) and JPT Peptide Technologies (Berlin, Germany) provided the Aβ40/42 and PHF6 peptides, respectively. 96-well microtiter plates (Greiner Bio-One GmbH, Frickenhausen, Germany) were used to perform the inhibition tests. A BioTek Synergy H1 plate reader (Agilent, Milan, Italy) was used for the incubations and readouts. Prism (version 5.01 for Windows, GraphPad Software, San Diego, CA, USA) was used to compute the inhibition data, which are expressed as mean ± SEM.

#### 4.2.1. Enzyme inhibition test

Commercial human recombinant AChE, MAO A and MAO B, and BuChE from equine serum were used by applying already published procedures.^20^ All compounds were initially tested in triplicate at single-point (10 µM) concentration and only for those showing >60% inhibition IC_50_ s were calculated in a concentration range of 30–0.01 µM. In all assays, measures of samples were always subtracted of blanks, in order to eliminate any possible interference from intrinsic fluorescence. Briefly, for cholinesterase inhibition the spectrophotometric method of Ellman was followed, measuring the absorbance at 412 nm in flat-bottomed transparent plates. MAO inhibition was performed in black, round-bottomed plates by measuring the fluorescence of 4-hydroxyquinoline as the oxidation product of MAO enzymes at 310/400 nm ex/em wavelength.

#### 4.2.2. Protein aggregation tests

Aβ40 was pretreated with 1,1,1,3,3,3-hexafluoroisopropanol (HFIP), then lyophilized and dissolved in phosphate buffered saline (PBS) before use. Similarly, work stock solutions were made by dissolving the PHF6 peptide in 1,1,1-trifluoroethanol (TFE) and then diluting it with PBS. Using black low-binding, flat-bottomed polystyrene 96-well microtiter plates, *in vitro* aggregation tests were carried out in triplicate using a spectrofluorimetric assay based on ThT fluorescence at excitation/emission wavelengths of 440/485 nm.

Aβ40 aggregation inhibition was carried out as described^20^ by incubating peptide (30 µM) and compounds (10 µM) in PBS containing 2% HFIP for 2 hours at 25 °C. After the addition of ThT (25 µM) to a free aggregating peptide sample, the fluorescence was measured and data expressed as a % of inhibition compared to a freely aggregating sample.

Following previously described procedures,^21^ compounds (10 µM) and ThT (10 µM) were incubated with tau peptide PHF6 (50 µM) in PBS. Incubations were carried out at 30 °C for 3 h, with continuous reading of ThT fluorescence.

### 4.3. Cellular assays

Confluent SH-SY5Y cells were incubated in DMEM serum-free for 24 h. After the incubation period, cell viability was assessed using the MTT assay.^22^ Results were expressed as % of cell viability referred to untreated cells (CTRL) and represent the mean ± SD of three independent experiments. Data analysis and statistics were obtained with Graph Pad Prism.

#### 4.3.1. Cytotoxicity assay

Compounds P10, P12, and P17 were tested at concentrations ranging from 0.1 to 100 µM. Validation of data was made with one-way analysis of variance (ANOVA) followed by multiple comparison tests (Dunnet's test). Levels of significance: **p < 0.001; ***p < 0.0001 referred to control.

#### 4.3.2. Neuroprotection assay

Compounds were incubated with 20 µM Aβ42 (for amyloid-induced neurotoxicity) or 300 µM H_2_O_2_ (for peroxide-induced insult) in the absence or in the co-presence of increasing concentrations of compounds P10, P12 and P17 (from 0.1 to 10 µM). Quercetin at 5 and 10 µM was used as standard control. Validation of data was made with one-way analysis of variance (ANOVA) followed by multiple comparison tests (Dunnet's test).

### 4.4. Computational study

#### 4.4.1. Molecular docking analysis

The three-dimensional monomeric Aβ1-42 NMR structure (PDB ID: 1IYT) and MAO-B (PDB ID: 2V61) were retrieved from the RCSB Protein Data Bank,^23^ and were selected based on their relevance to neurodegenerative disorders, particularly AD and MAO-associated pathways. The retrieved proteins were prepared by removing water molecules, co-crystallized ligands, and heteroatoms, followed by the addition of polar hydrogen atoms and assignment of Kollman charges using AutoDock Tools. The structures were then energy-minimized, protonated using Open Babel, and converted into PDBQT format for docking studies. Molecular docking was carried out using AutoDock Vina v1.2.5.^24^ For Aβ (PDB ID:1IYT), the grid box was defined with center coordinates *x* = −2.4700, *y* = −0.0652, and *z* = 1.2694, and dimensions of 37.3694 Å × 22.3151 Å × 49.5333 Å. For MAO-B (PDB ID: 2V61), the grid box was centered at *x* = 53.096, *y* = 141.478, and *z* = 17.648, with dimensions of 15 Å × 23.25 Å × 19.5 Å, covering the active binding region. Docking results were evaluated based on the predicted binding affinity (kcal mol^−1^) and RMSD values reported by AutoDock Vina. More negative docking scores indicate more favorable predicted ligand-target interactions within the Vina scoring model. The 0.000 Å RMSD value reported for the top-ranked pose represents the reference pose used for pose comparison and should not be interpreted as an independent measure of docking accuracy or binding stability. The docked protein–ligand complexes were investigated using PyMOL and Discovery Studio to recognize key interactions, including hydrophobic contacts, hydrogen bonds, and π–π stacking, which were used to characterize the predicted ligand-target interaction patterns and binding orientation. For Aβ, these interactions were interpreted within the ligand-interaction region of the monomeric Aβ1-42 structure rather than as an active site.

#### 4.4.2. Molecular dynamics simulation

Molecular dynamics (MD) simulations were implemented through Desmond (Molecular Dynamics Desmond's engine)^25^ to investigate the dynamic behavior and conformational properties of the protein–ligand complexes. An OPLS-based force field was used, and the system was solvated in a TIP3P water model within an orthorhombic simulation box. To neutralize the system, periodic boundary parameters were applied, and appropriate counter ions were included. 100-nanosecond (ns) simulations were carried out under the NPT (isothermal-isobaric ensemble) configuration at 1 atm pressure and 300 K temperature. Trajectory analysis was performed to characterize structural deviations, conformational flexibility, molecular compactness, and solvent exposure throughout the simulation period. Key parameters, such as root mean square deviation (RMSD), root mean square fluctuation (RMSF), molecular surface area (MolSA), polar surface area (PSA), radius of gyration (rGyr), and solvent-accessible surface area (SASA), were examined. These parameters were evaluated collectively to characterize the dynamic and conformational behavior of the simulated protein–ligand systems.

#### 4.4.3. ADME analysis and physicochemical properties

ADME parameters and physicochemical properties of the selected compounds were measured using the QikProp program in Schrodinger. Geometry-optimized ligand structures were utilized as input for the calculation of key parameters related to physicochemical and ADME properties. Parameters, including molecular weight (MW), hydrogen bond donors (HBD), hydrogen bond acceptors (HBA), human oral absorption octanol/water partition coefficient (QPlogPo/w), polar surface area (PSA), and brain/blood partition coefficient (QPlogBB), were characterized. The predicted values were used to provide preliminary computational estimates of oral absorption, lipophilicity, polar surface characteristics, and brain/blood distribution. These predictions were interpreted as computational indicators and were not considered as experimental confirmation of pharmacokinetic properties or blood–brain barrier (BBB) penetration, as reported in previous studies.^26^

## Author contributions

R. P: designed the molecule, standardization of synthetic protocol, synthesis of compounds, analyzed NMR data, compiled the results, and wrote the original draft of the manuscript. A Reza and R. P: planned and performed *in silico* studies and contributed to manuscript writing. T. Y. C and A. R: reviewed and edited the original manuscript. A. G: performed *in vitro* MAO and ChEs inhibition assays. G. L. S: performed *in vitro* antiaggregating assays. D. V. M: performed cell assays. M Cat: supervised the *in vitro* biological studies, provided resources, and helped in writing the manuscript. N. H: supervised the synthetic work, edited the manuscript, and arranged the funds for this project. All authors read and approved the final manuscript.

## Conflicts of interest

The authors declare no conflicts of interest.

## Supplementary Material

RA-016-D6RA07131G-s001

## Data Availability

The data that support the findings of this study are available in the supplementary information (SI) of this article. Supplementary information is available. See DOI: https://doi.org/10.1039/d6ra07131g.
