# Supplementary material for: Design, synthesis, and biological evaluation of naphthalimide derivatives as dual monoamine oxidase-B and amyloid-β aggregation inhibitors for Alzheimer's disease
Source: RSC Adv. 2026 Oct 6;16(46):60370–85. doi: 10.1039/d6ra07131g (PMC13639867; doi:10.1039/d6ra07131g)
Supplement: Supplementary file 1 [file RA-016-D6RA07131G-s001.pdf]

Supplementary Information (SI) for RSC Advances.
This journal is © The Royal Society of Chemistry

Design, Synthesis, and Biological Evaluation of Naphthalimide Derivatives as Dual Monoamine Oxidase-B and Amyloid-β Aggregation Inhibitors for Alzheimer's Disease

Rukshar Praveen,[a] Alessandro Gadaleta,[b] Gabriella La Spada,[b] Daniela Valeria Miniero,[c] Aquib Reza,[d] Tohasib Yusub Chaudhari,[a] Abdur Rahman,[a] Marco Catto,*[b] Nasimul Hoda*[a]

[a] *Drug Design and Synthesis Lab., Department of Chemistry, Jamia Millia Islamia, New Delhi-110025, India*

[b] *Department of Pharmacy-Pharmaceutical Sciences, University of Bari Aldo Moro, Via E. Orabona 4, 70125 Bari (Italy)*

[c] *Department of Medicine and Surgery, LUM University Giuseppe Degennaro, Torre Rossi, 70010 Casamassima, Italy*

[d] *Department of Molecular Medicine, Jamia Hamdard, New Delhi, 110062, India*

*Corresponding authors

Prof. Nasimul Hoda
Department of Chemistry,
Jamia Millia Islamia,
Jamia Nagar, New Delhi-110025, India
Tel.: +0091-9910200655;
Fax: +0091-11-26985507;
E-mail: nhoda@jmi.ac.in

Prof. Marco Catto
Department of Pharmacy-Pharmaceutical Sciences
University of Bari Aldo Moro,
Via E. Orabona 4, 70125 Bari (Italy)
E-mail: marco.catto@uniba.it

Table of Contents

1. ^{1}H NMR and ^{13}C NMR spectra of compounds 3 and P1-P18 **S3-S21**
2. HRMS data of compounds P1-P18 **S22-S27**
3. ^{19}F NMR of compound P8 **S-28**

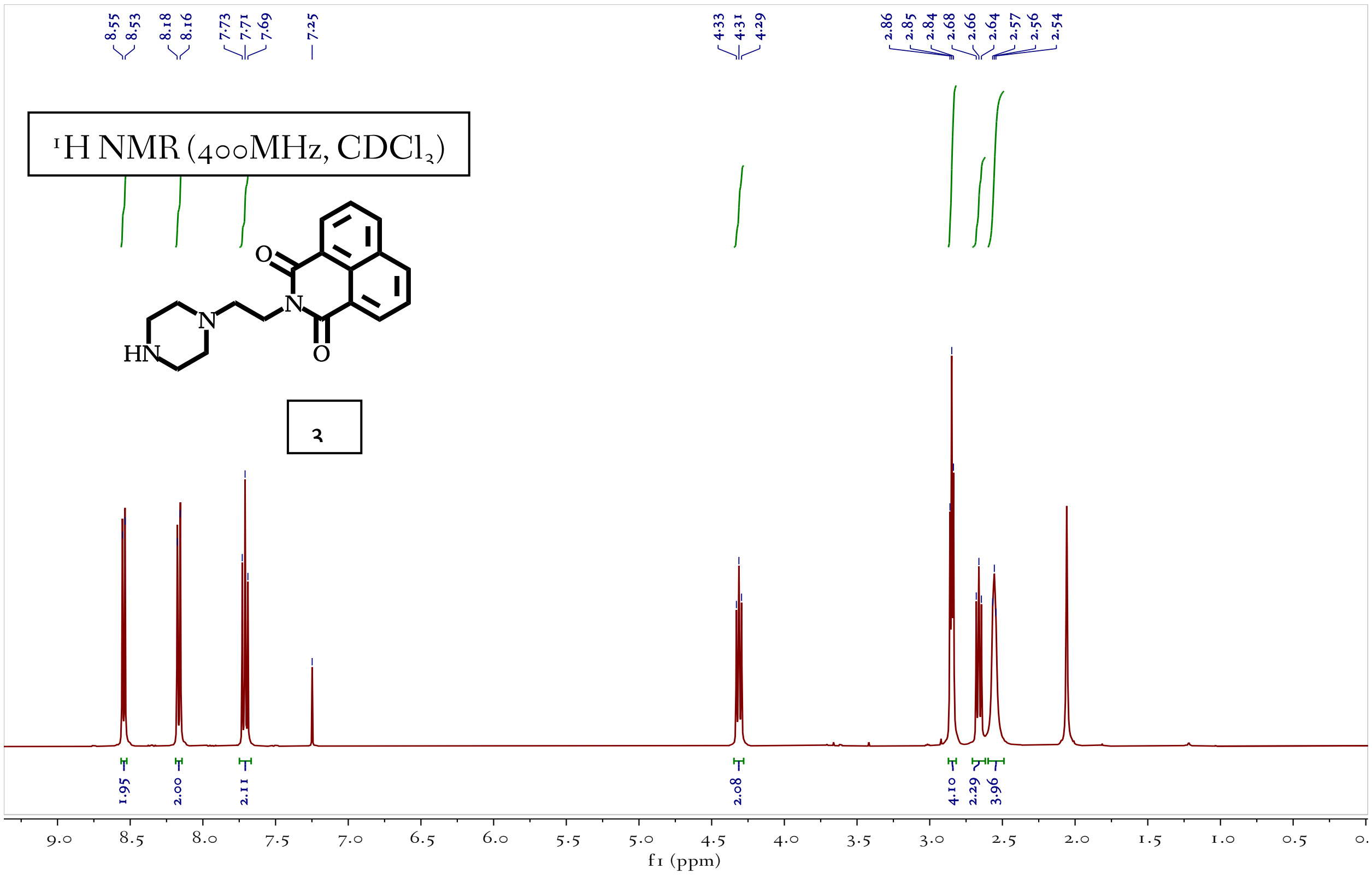
8.55
8.53
8.18
8.16
7.73
7.71
7.69
7.25
4.33
4.31
4.29
2.86
2.85
2.84
2.68
2.66
2.64
2.57
2.56
2.54
¹H NMR (400MHz, CDCl₃)
HN
N
N
O
O
2
1.95
2.00
2.11
2.08
4.10
2.29
3.96
f1 (ppm)

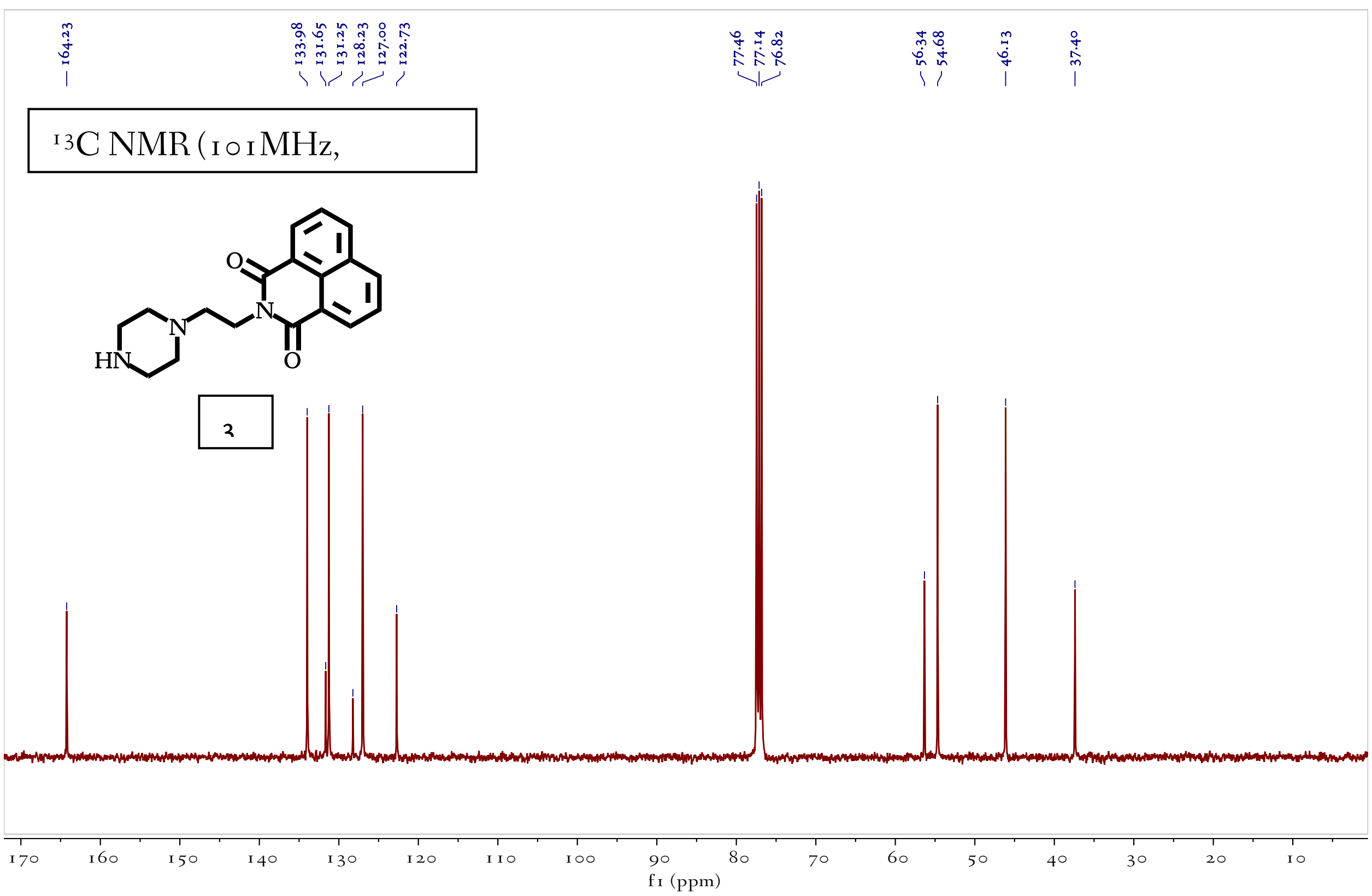
164.23
133.98
131.65
131.25
128.23
127.00
122.73
77.46
77.14
76.82
56.34
54.68
46.13
37.40
¹³C NMR (101MHz,
HN
N
N
O
O
2
f1 (ppm)

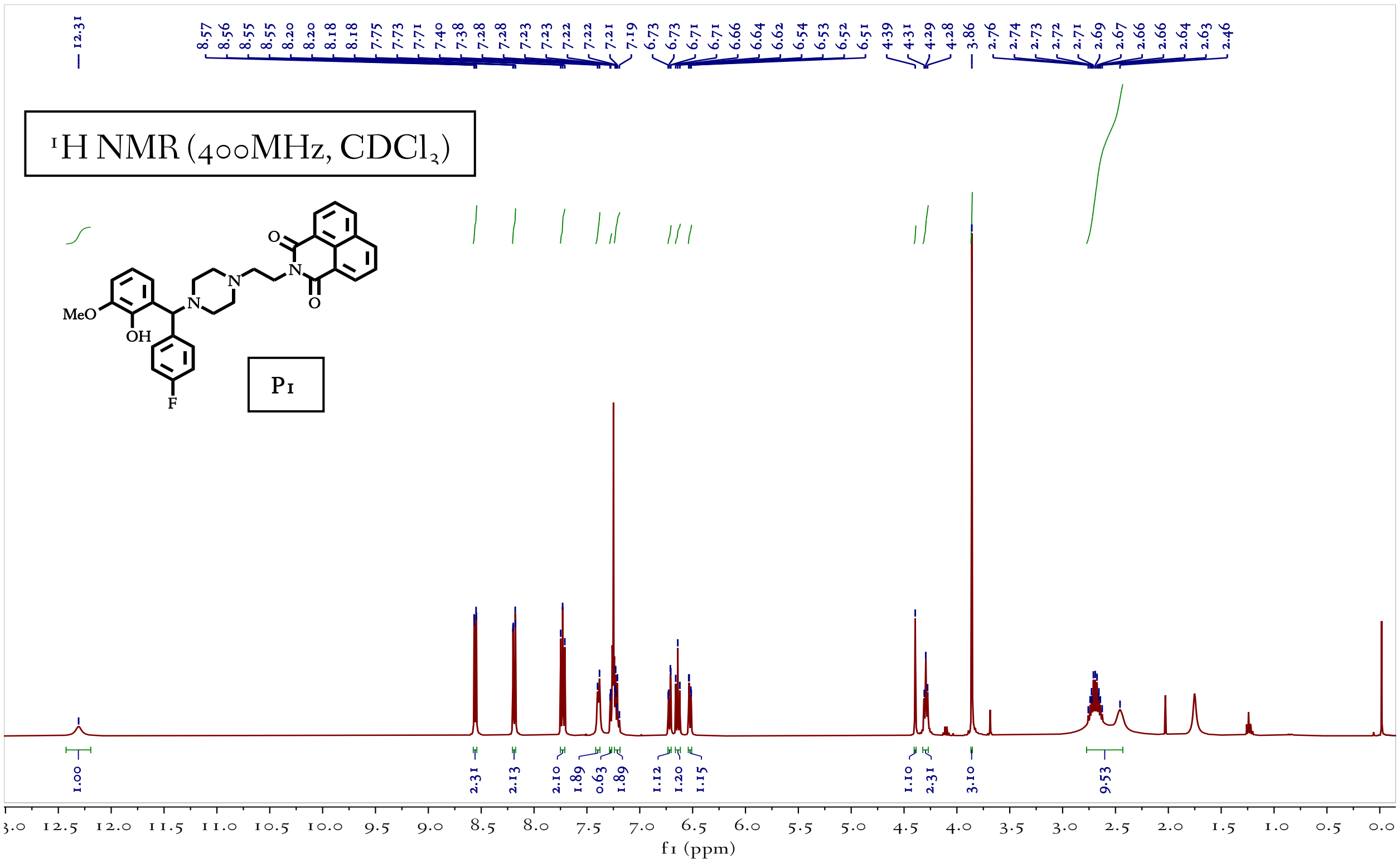

¹H NMR (400MHz, CDCl₃)
P1
MeO
OH
F
N
O
12.31
8.57 8.56 8.55 8.55 8.20 8.18 8.18 8.18 7.75 7.73 7.73 7.71 7.40 7.38 7.28 7.28 7.23 7.23 7.22 7.22 7.21 7.19 6.73 6.73 6.71 6.71 6.66 6.64 6.62 6.54 6.53 6.52 6.51
4.39 4.31 4.29 4.28 3.86 2.76 2.74 2.73 2.72 2.71 2.69 2.67 2.66 2.66 2.64 2.63 2.46
1.00 2.31 2.13 2.10 1.89 0.63 1.89 1.12 1.20 1.15 1.10 2.31 3.10 9.53
f1 (ppm)

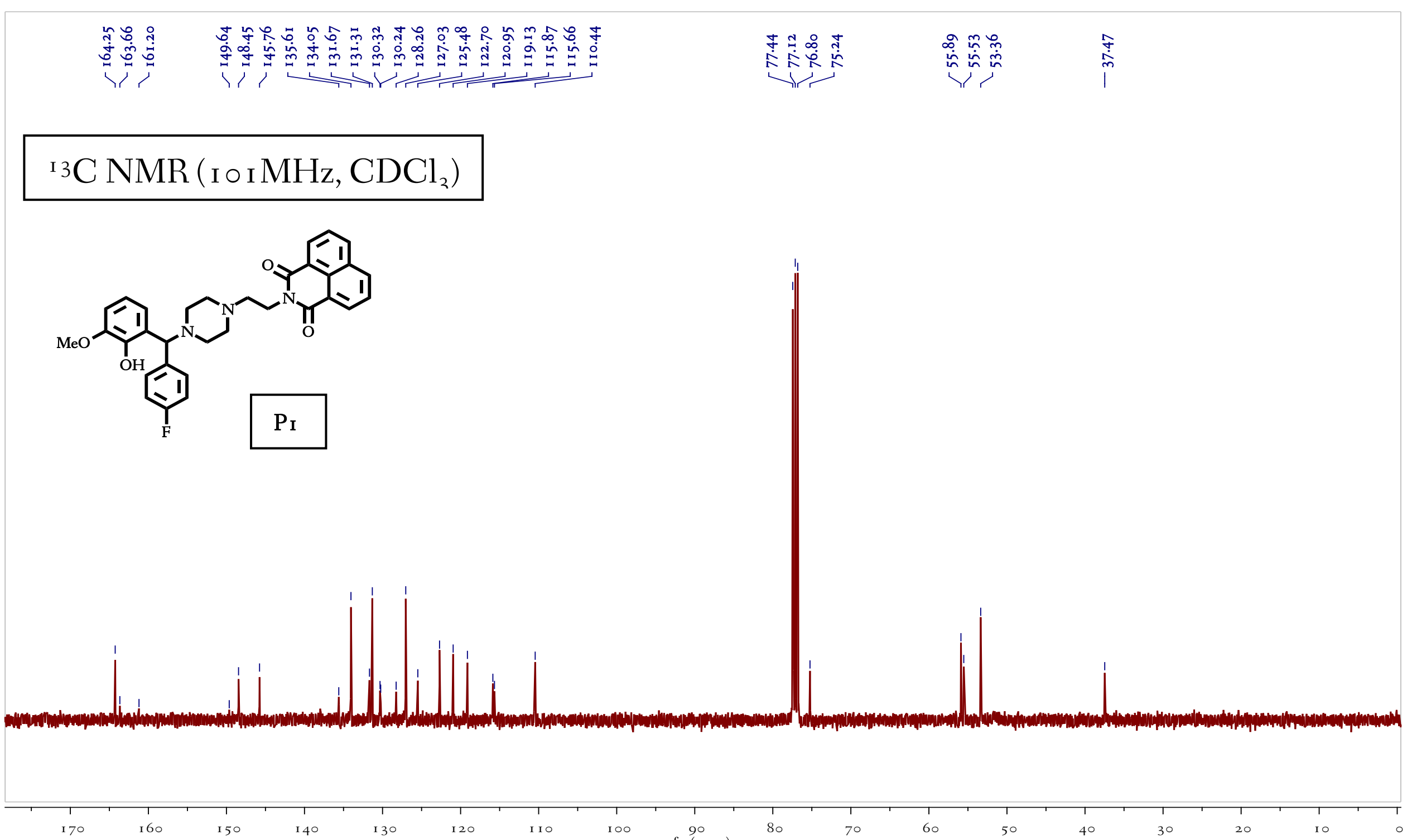

¹³C NMR (101MHz, CDCl₃)
P1
MeO
OH
F
N
O
164.25 163.66 161.20 149.64 148.45 145.76 135.61 134.05 131.67 131.31 130.32 130.24 128.26 127.03 125.48 122.70 120.95 119.13 115.87 115.66 110.44
77.44 77.12 76.80 75.24 55.89 55.53 53.36 37.47
f1 (ppm)

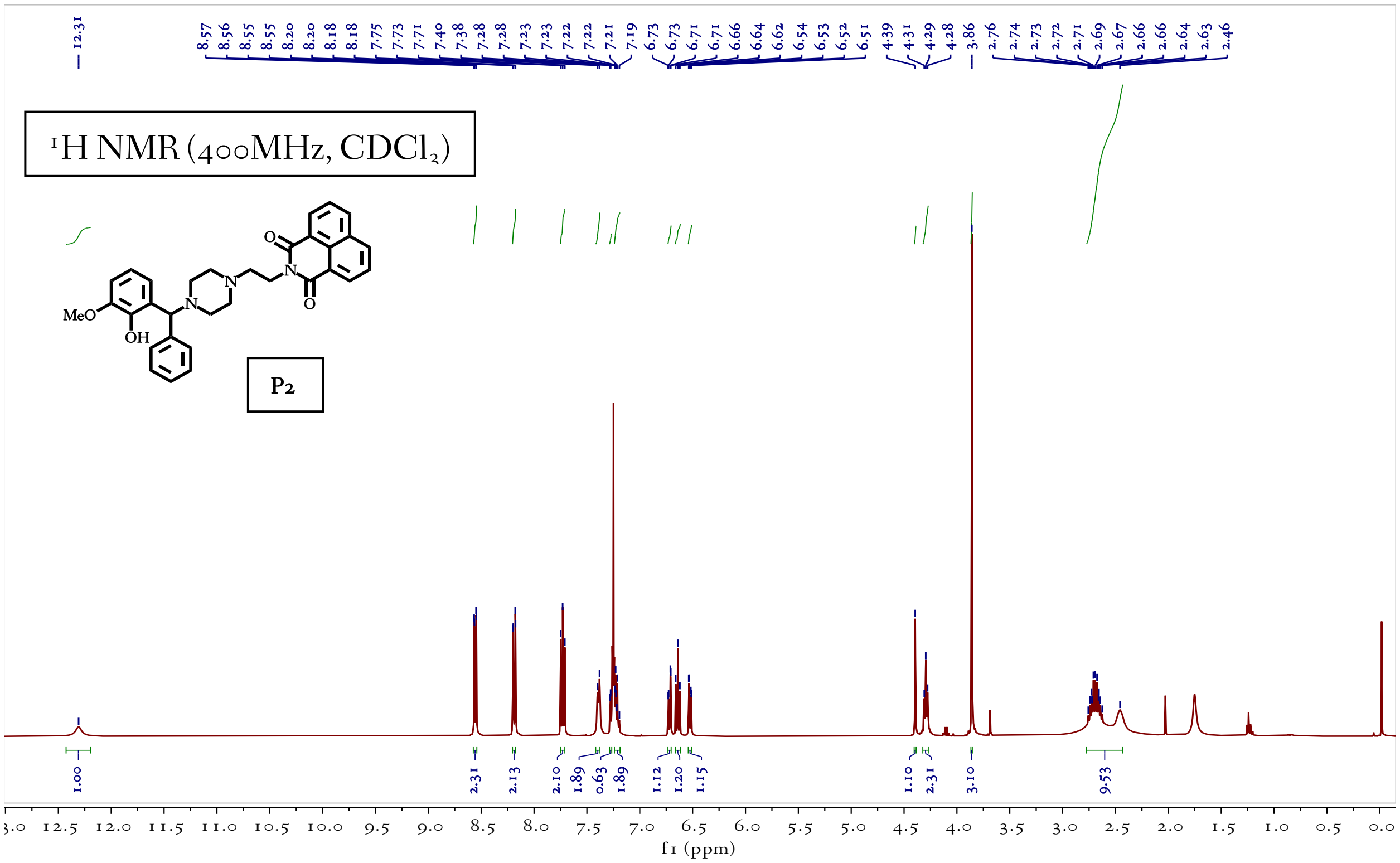
1H NMR (400MHz, CDCl3)
MeO
OH
O
N
P2
f1 (ppm)

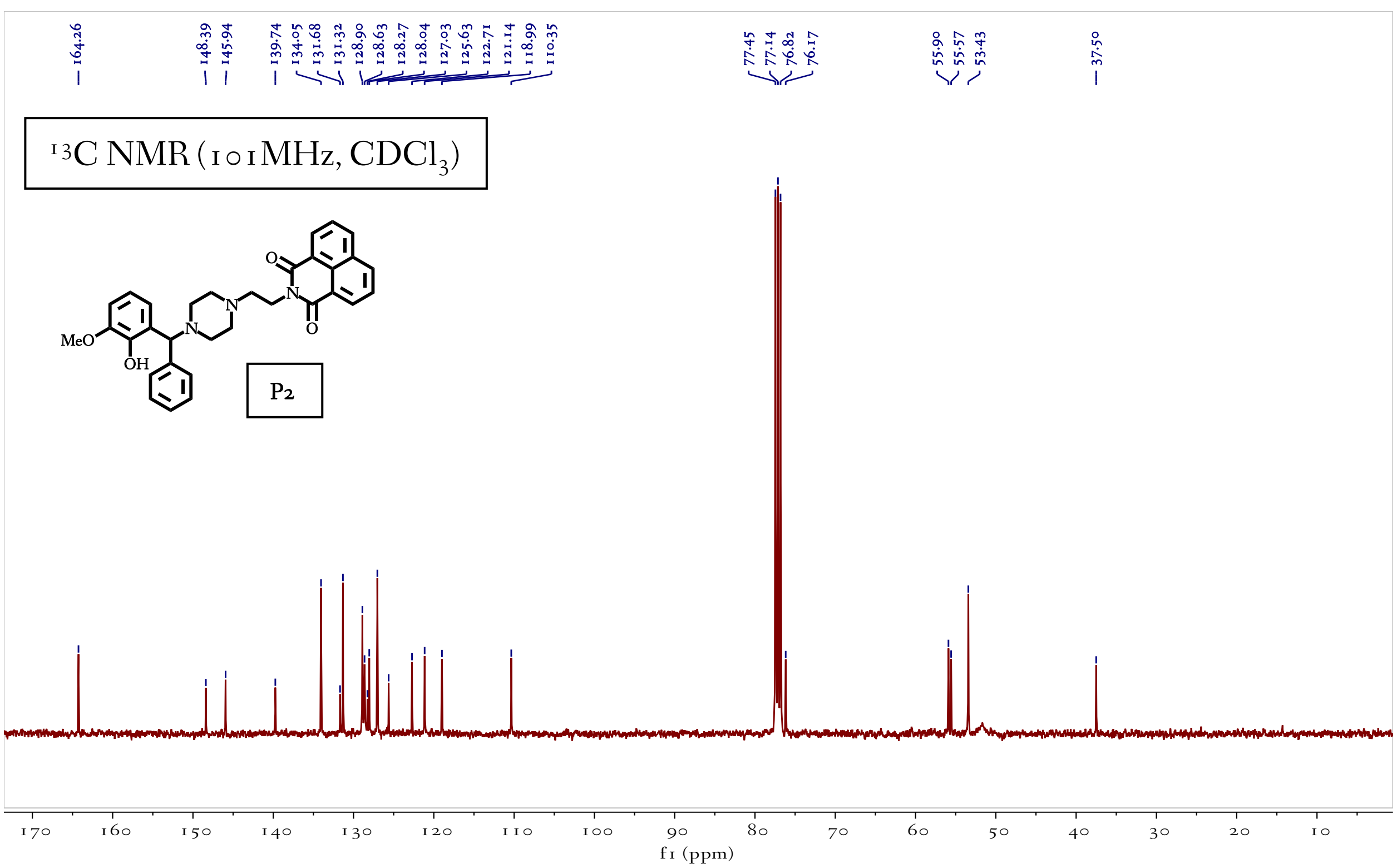
13C NMR (101MHz, CDCl3)
MeO
OH
O
N
P2
f1 (ppm)

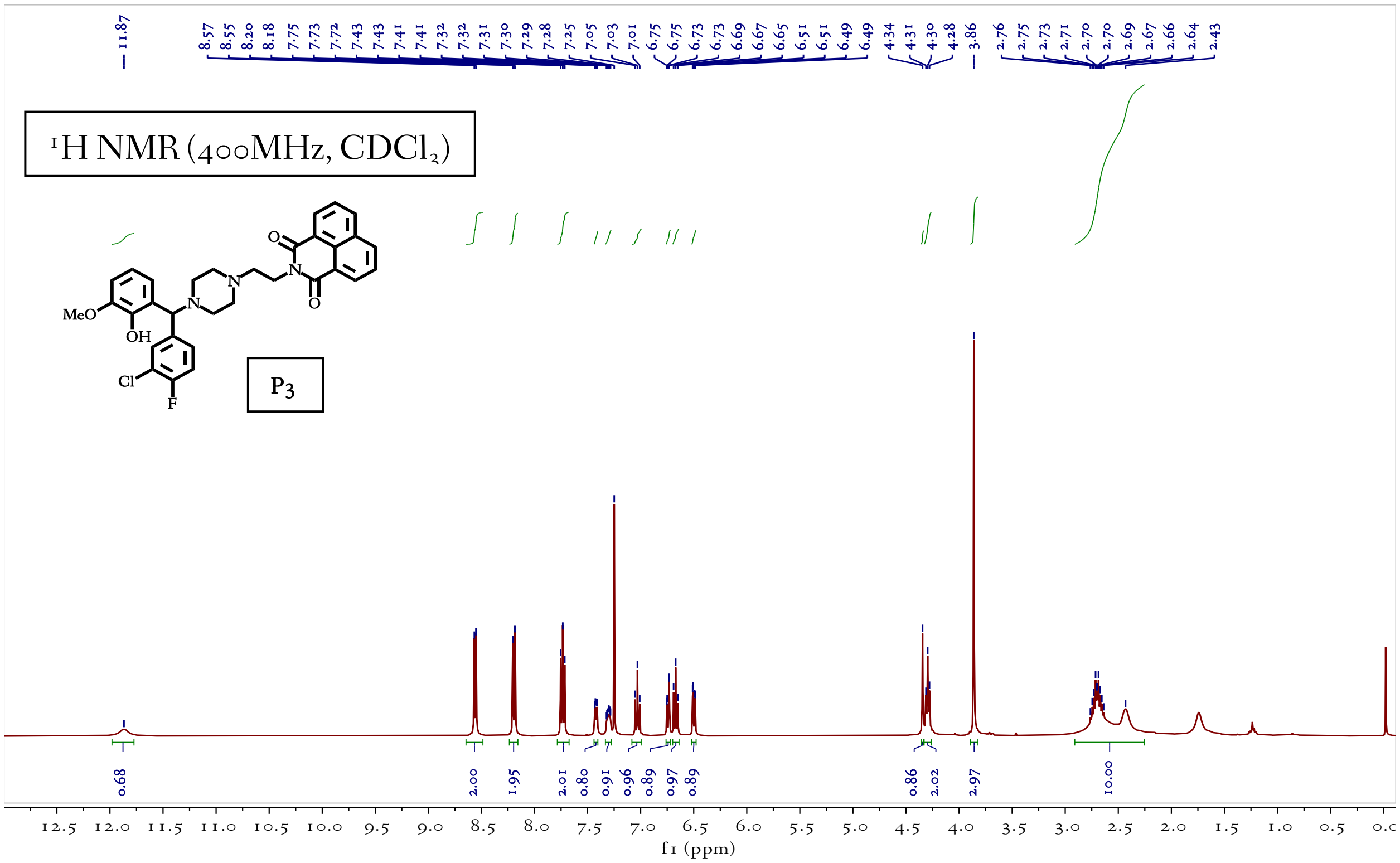

¹H NMR (400MHz, CDCl₃)
MeO
OH
Cl
F
N
O
P3
f1 (ppm)

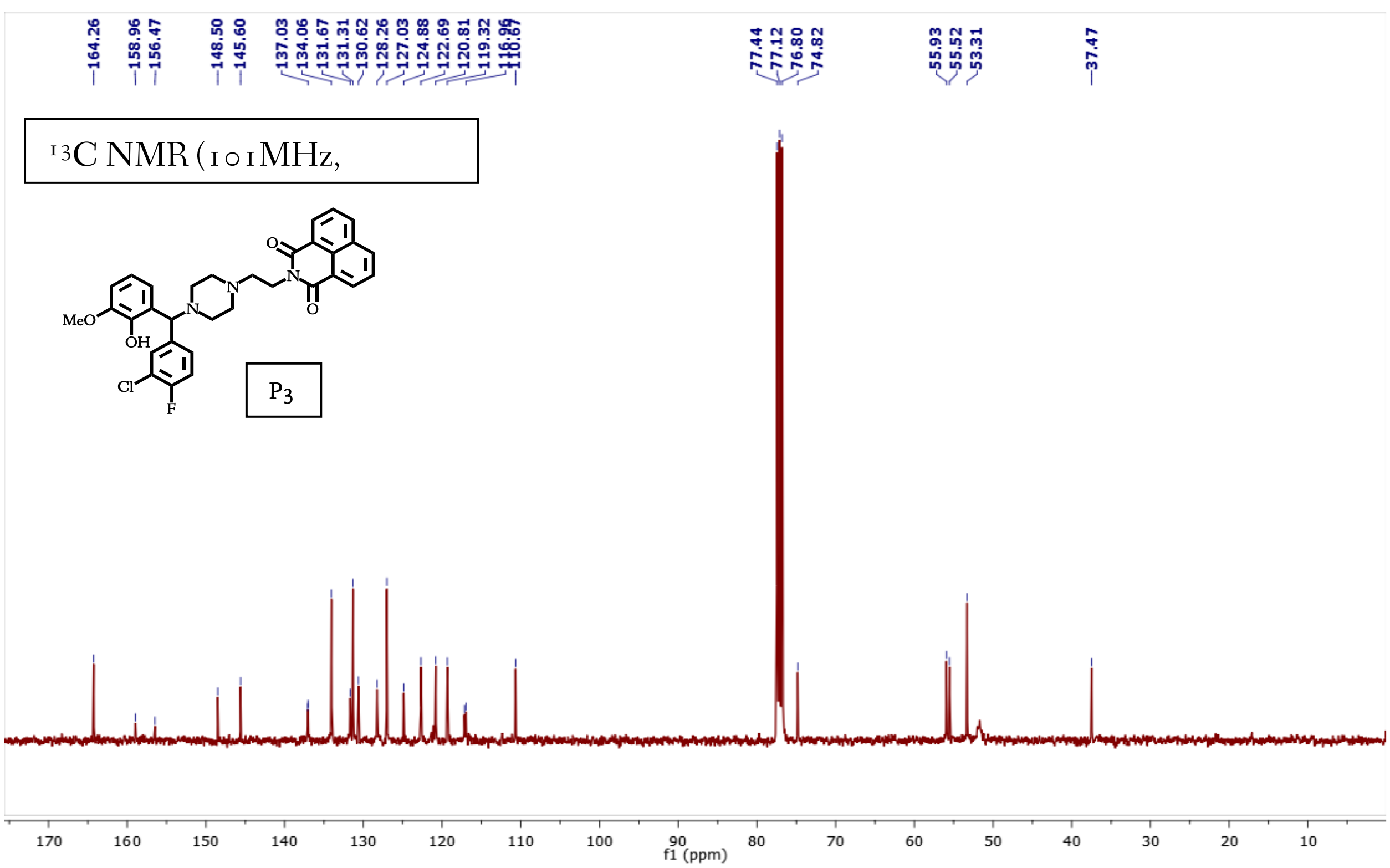

¹³C NMR (101MHz,
MeO
OH
Cl
F
N
O
P3
f1 (ppm)

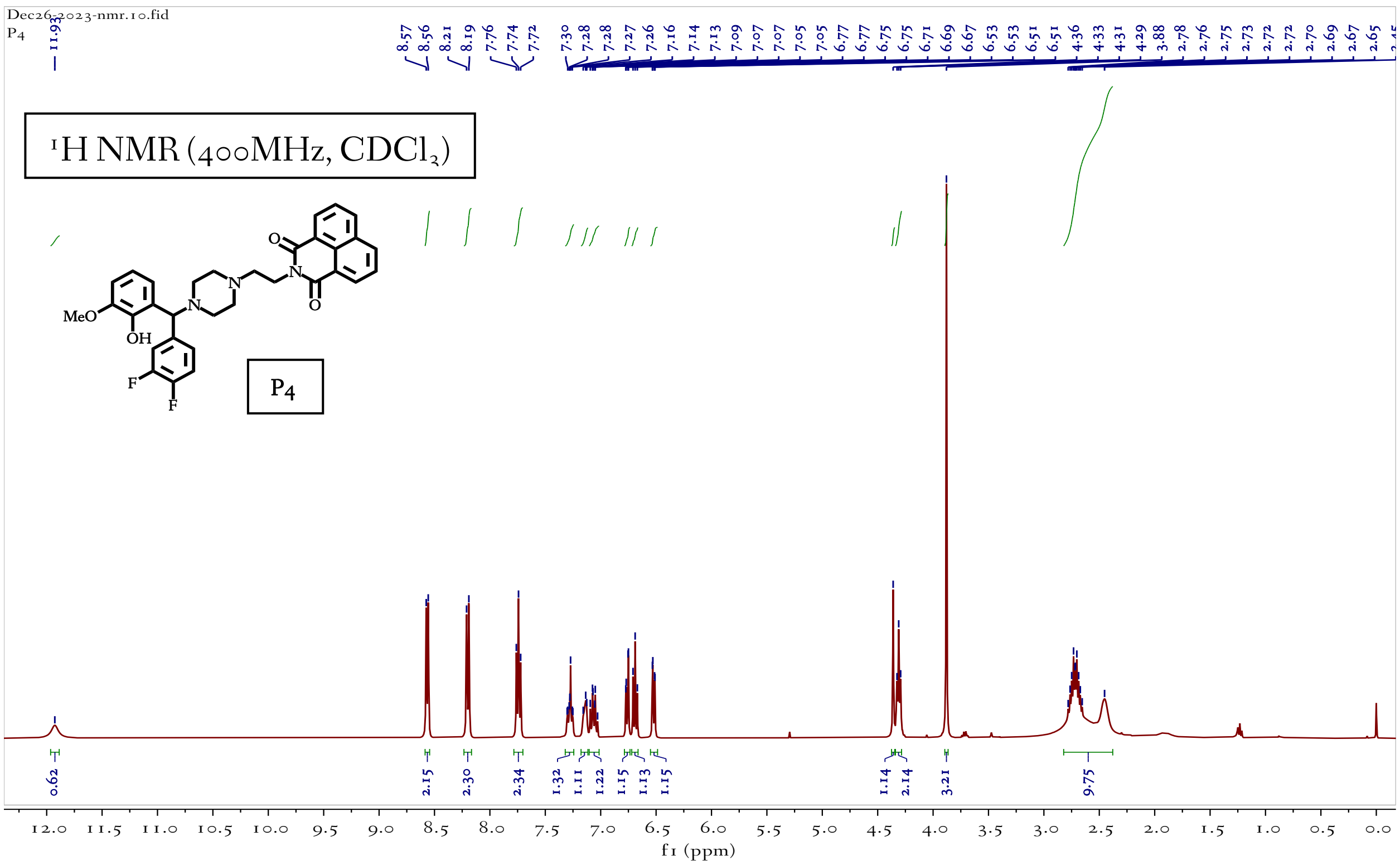

Dec26-2023-nmr.10.fid
P4
¹H NMR (400MHz, CDCl₃)
MeO
OH
F
F
N
N
N
O
O
P4
f1 (ppm)

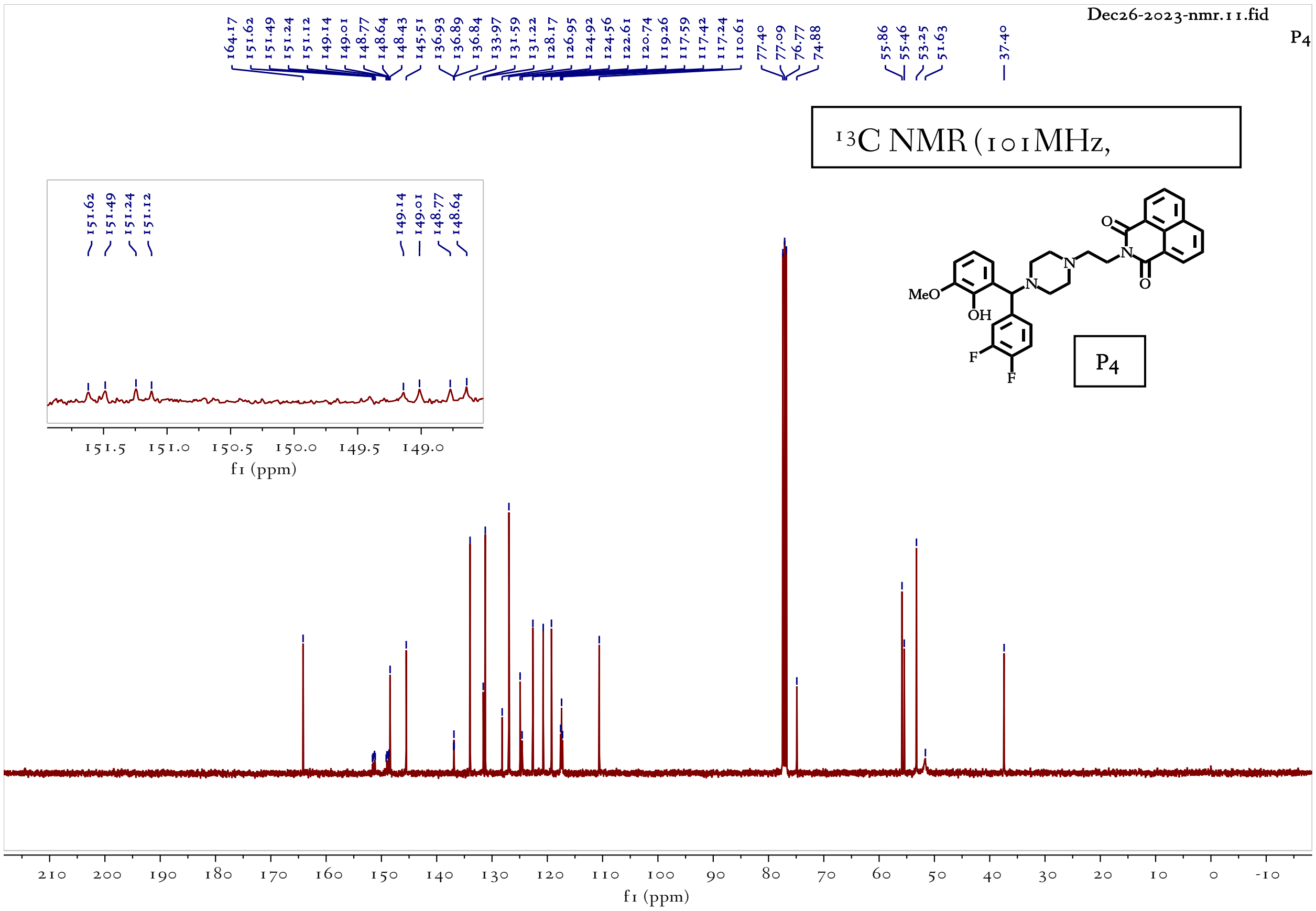
Dec26-2023-nmr.11.fid
P4
164.17 151.62 151.49 151.24 151.12 149.14 149.01 148.77 148.64 148.43 145.51 136.93 136.89 136.84 133.97 131.59 131.22 128.17 126.95 124.92 124.56 122.61 120.74 119.26 117.59 117.42 117.24 110.61 77.40 77.09 76.77 74.88 55.86 55.46 53.25 51.63 37.40
13C NMR (101MHz,
151.62 151.49 151.24 151.12 149.14 149.01 148.77 148.64
f1 (ppm)
MeO
OH
F
F
N
N
N
O
O
P4
f1 (ppm)

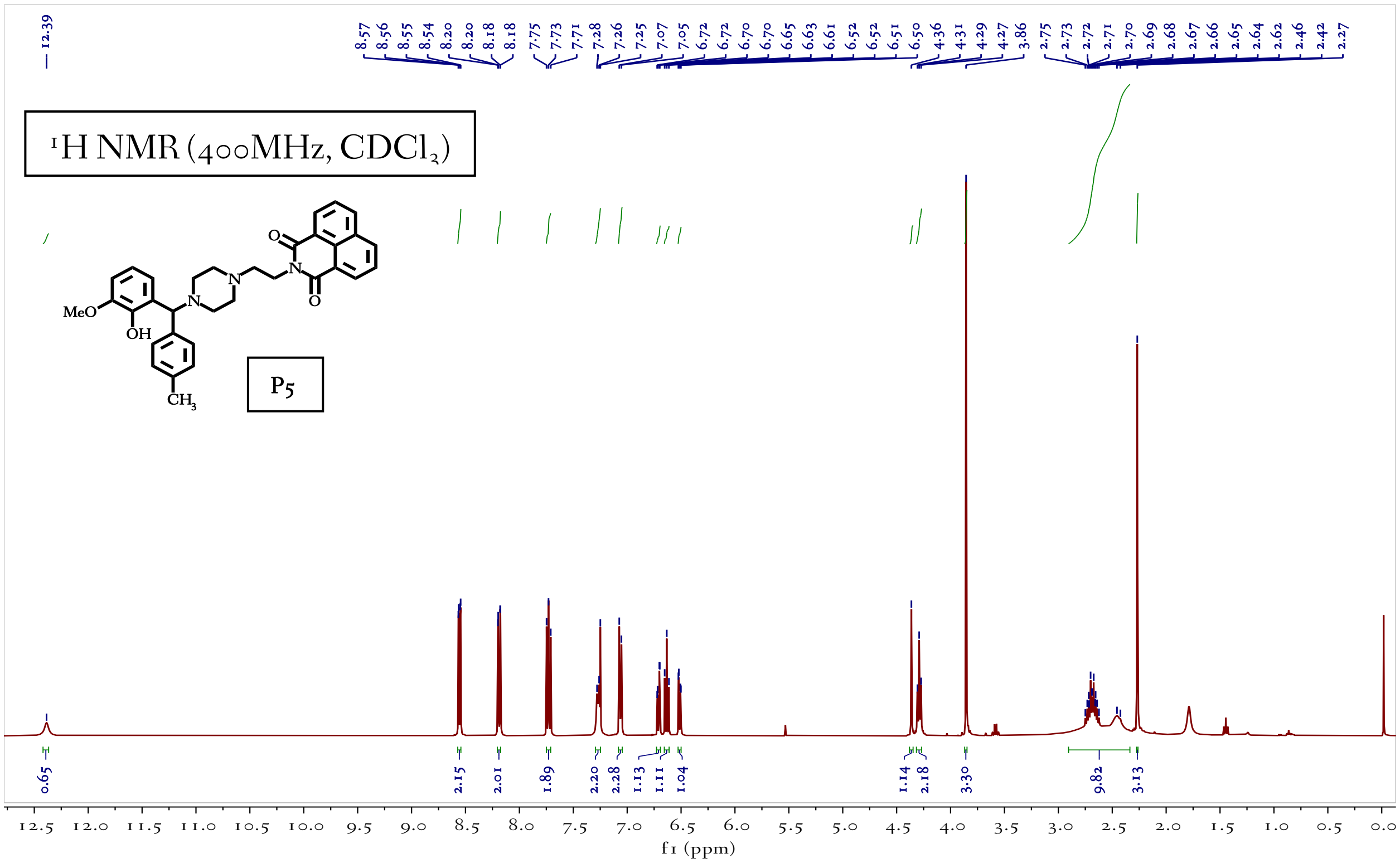

1H NMR (400MHz, CDCl3)
P5
MeO
OH
CH3
12.39
8.57 8.56 8.55 8.54 8.20 8.18 8.18 7.75 7.73 7.71 7.28 7.26 7.25 7.07 7.05 6.72 6.72 6.70 6.70 6.65 6.63 6.61 6.52 6.52 6.51 6.50 4.36 4.31 4.29 4.27 3.86 2.75 2.73 2.72 2.71 2.70 2.69 2.68 2.67 2.66 2.65 2.64 2.62 2.46 2.42 2.27
0.65 2.15 2.01 1.89 2.20 2.28 1.13 1.11 1.04 1.14 2.18 3.30 9.82 3.13
f1 (ppm)

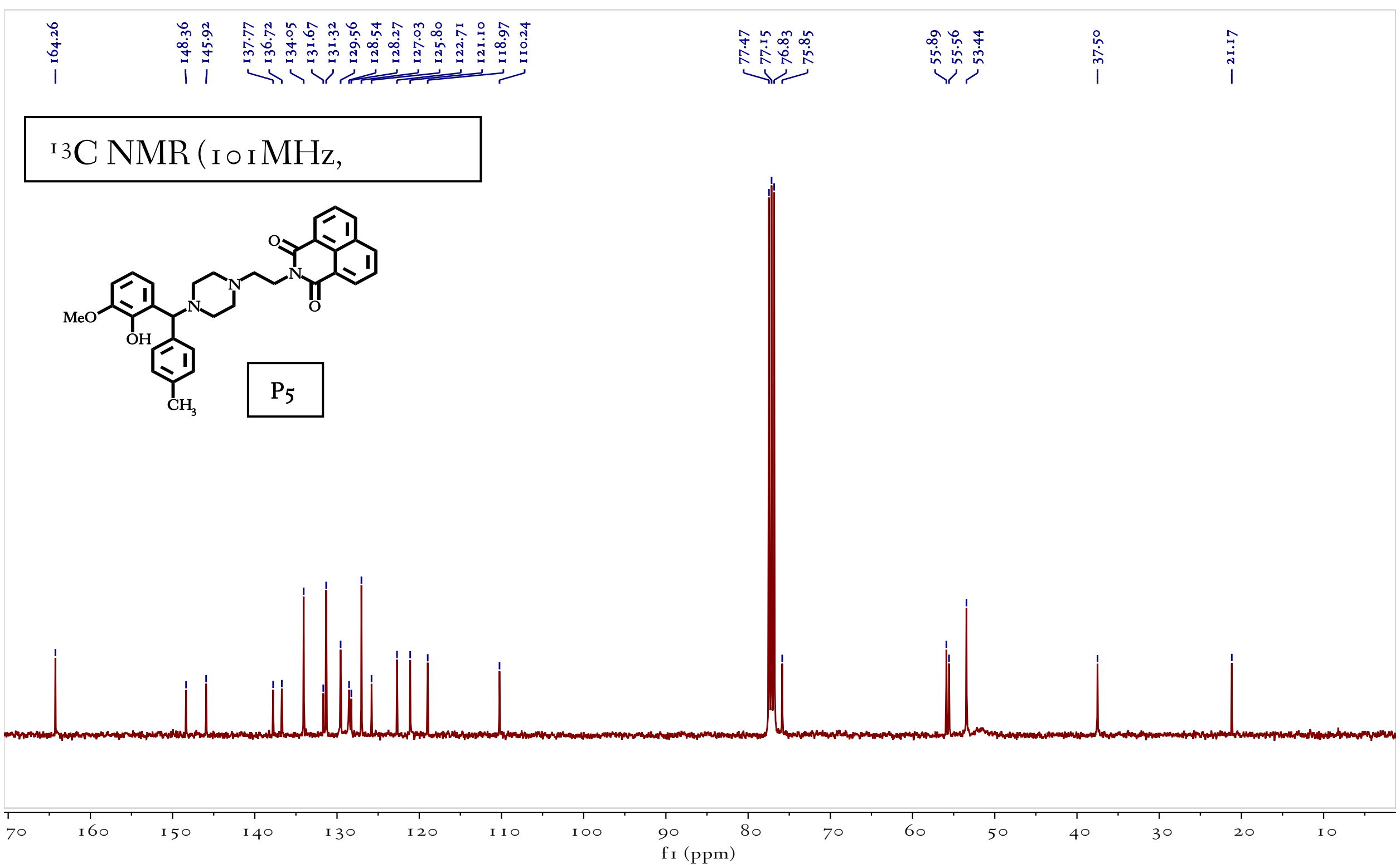

13C NMR (101MHz,
P5
MeO
OH
CH3
164.26
148.36 145.92
137.77 136.72 134.05 131.67 131.32 129.56 128.54 128.27 127.03 125.80 122.71 121.10 118.97 110.24
77.47 77.15 76.83 75.85
55.89 55.56 53.44
37.50
21.17
f1 (ppm)

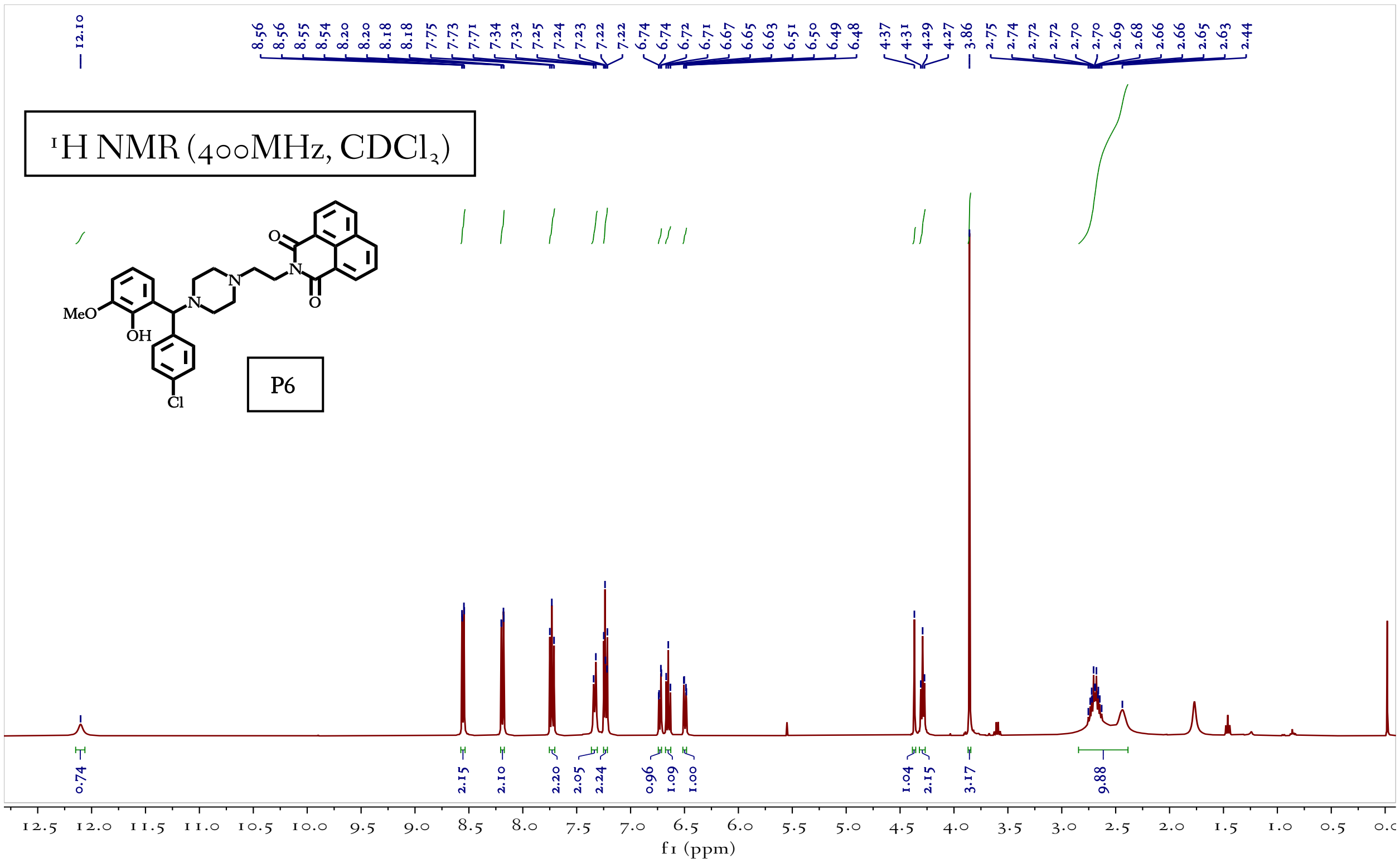

12.10
8.56 8.56 8.55 8.54 8.20 8.20 8.18 8.18 7.75 7.73 7.71 7.34 7.32 7.25 7.24 7.23 7.22 7.22 6.74 6.74 6.72 6.71 6.67 6.65 6.63 6.51 6.50 6.49 6.48
4.37 4.31 4.29 4.27 3.86
2.75 2.74 2.72 2.72 2.70 2.70 2.69 2.68 2.66 2.66 2.65 2.63 2.44
1H NMR (400MHz, CDCl3)
MeO
OH
Cl
N
O
P6
0.74 2.15 2.10 2.20 2.05 2.24 0.96 1.09 1.00 1.04 2.15 3.17 9.88
f1 (ppm)

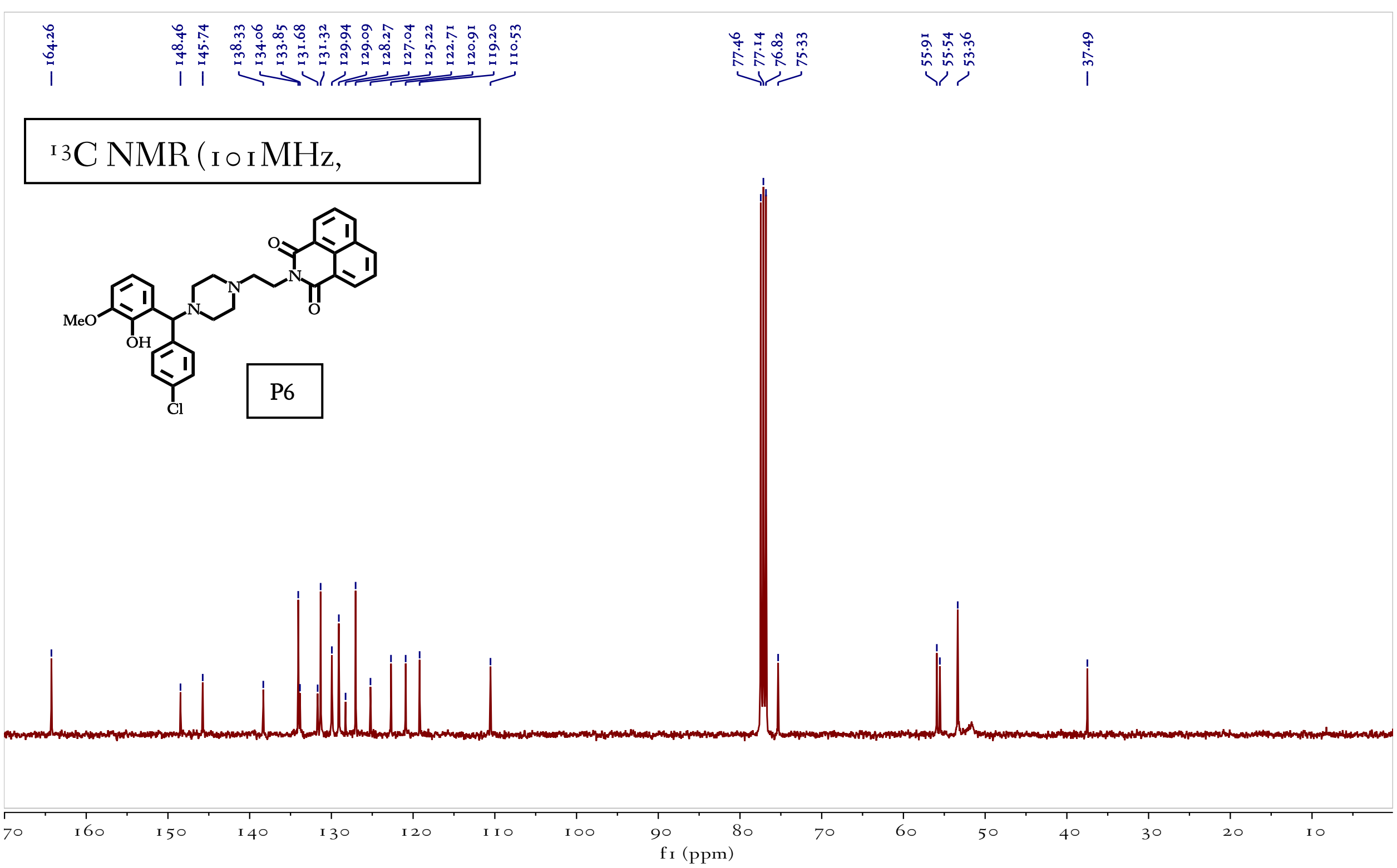

164.26
148.46 145.74
138.33 134.06 133.85 131.68 131.32 129.94 129.09 128.27 127.04 125.22 122.71 120.91 119.20 110.53
77.46 77.14 76.82 75.33
55.91 55.54 53.36
37.49
13C NMR (101MHz,
MeO
OH
Cl
N
O
P6
f1 (ppm)

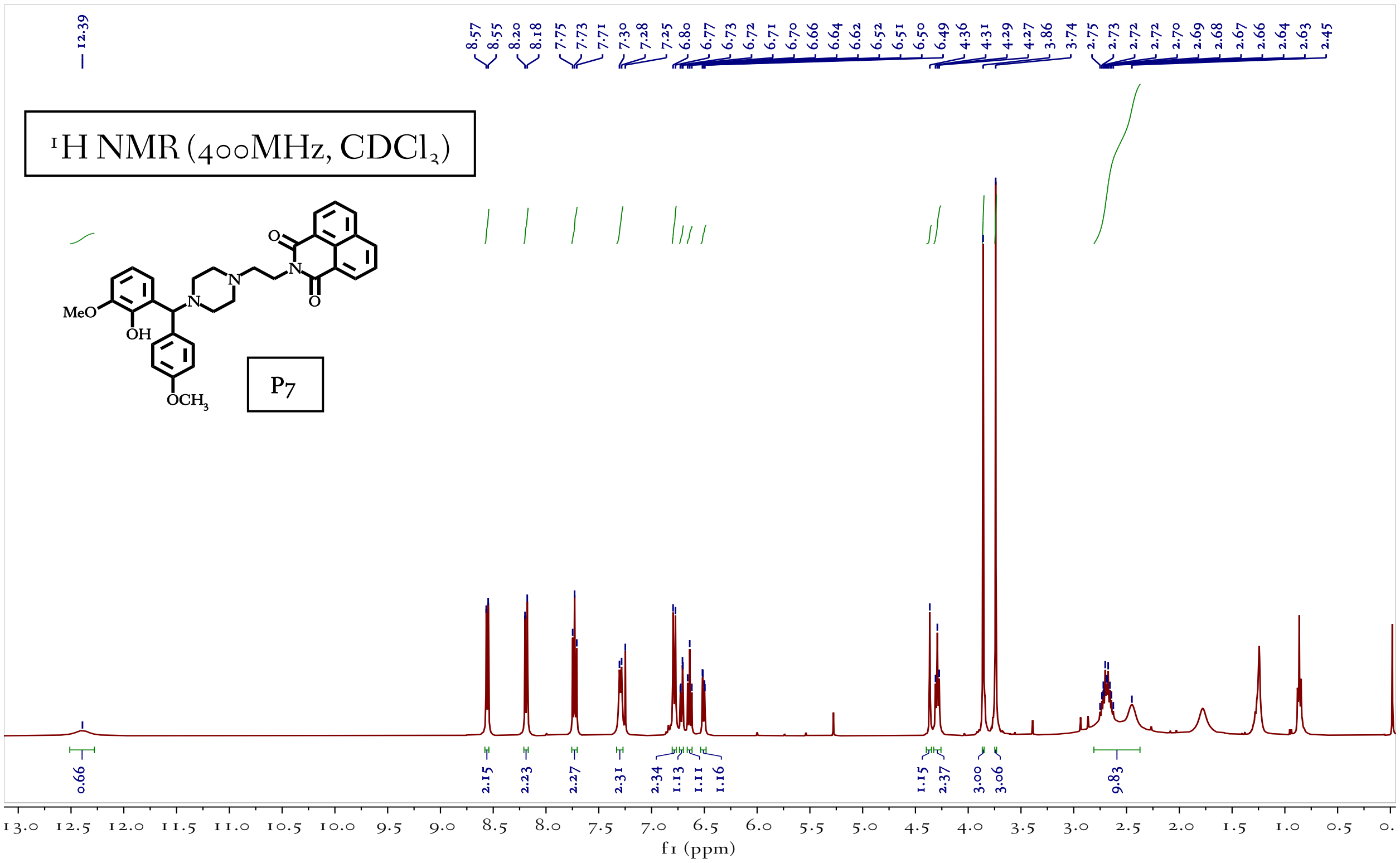
¹H NMR (400MHz, CDCl₃)
P7
f1 (ppm)

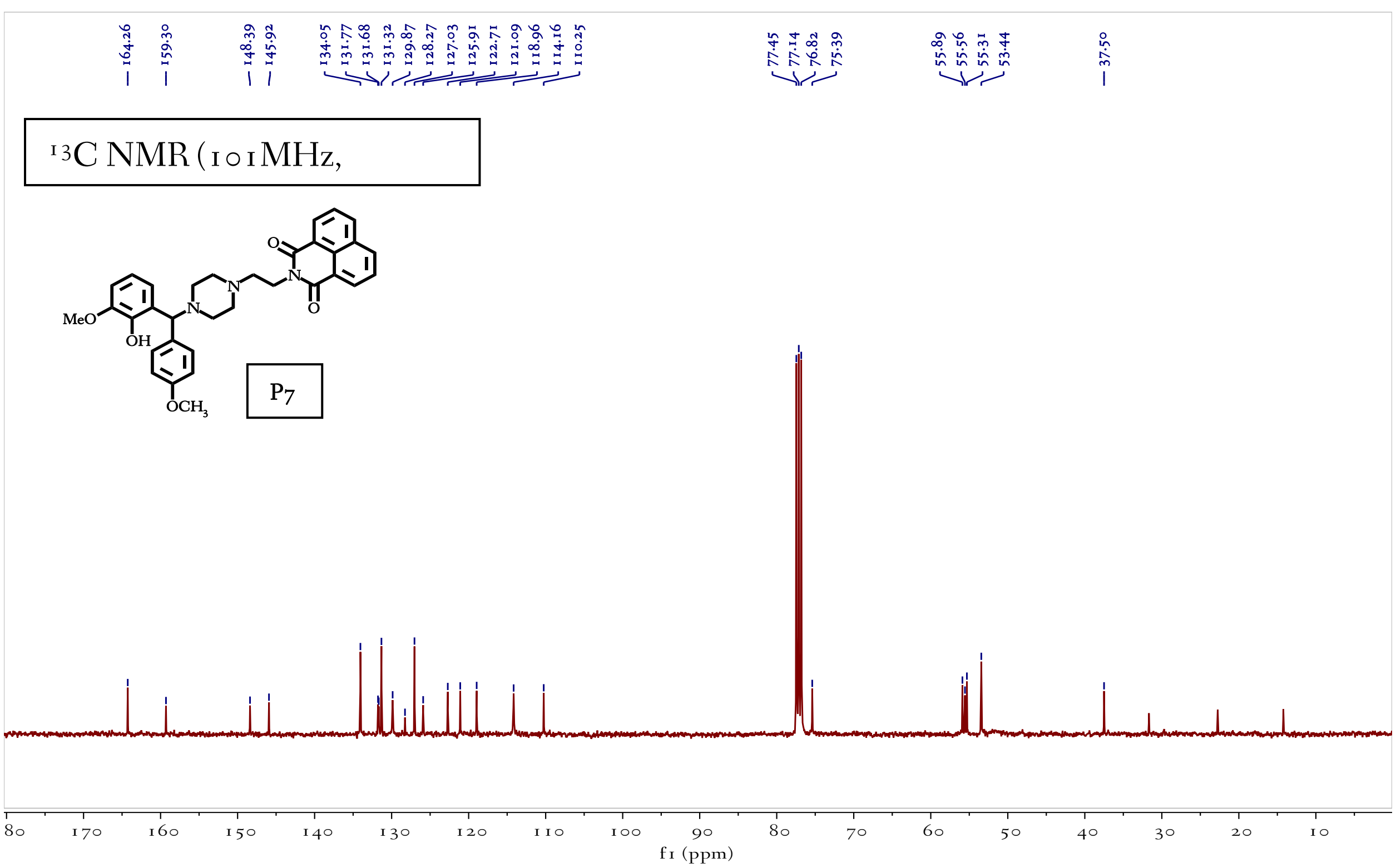
¹³C NMR (101MHz,
P7
f1 (ppm)

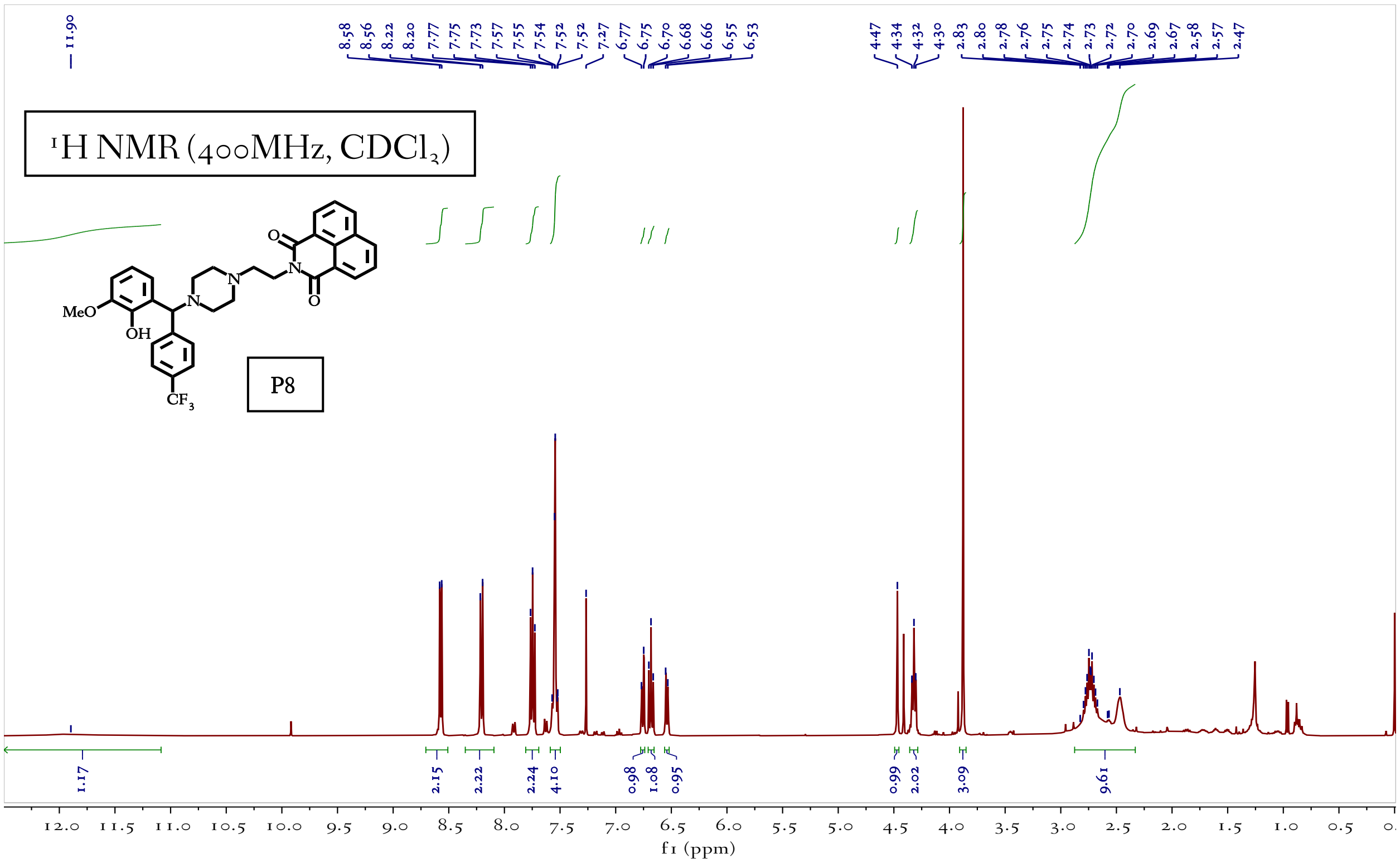

1H NMR (400MHz, CDCl3)
MeO
OH
CF3
N
O
P8
11.90
8.58 8.56 8.22 8.20 7.77 7.75 7.73 7.57 7.55 7.54 7.52 7.52 7.27 6.77 6.75 6.70 6.68 6.66 6.55 6.53
4.47 4.34 4.32 4.30 2.83 2.80 2.78 2.76 2.75 2.74 2.73 2.72 2.70 2.69 2.67 2.58 2.57 2.47
1.17 2.15 2.22 2.24 4.10 0.98 1.08 0.95 0.99 2.02 3.09 9.61
f1 (ppm)

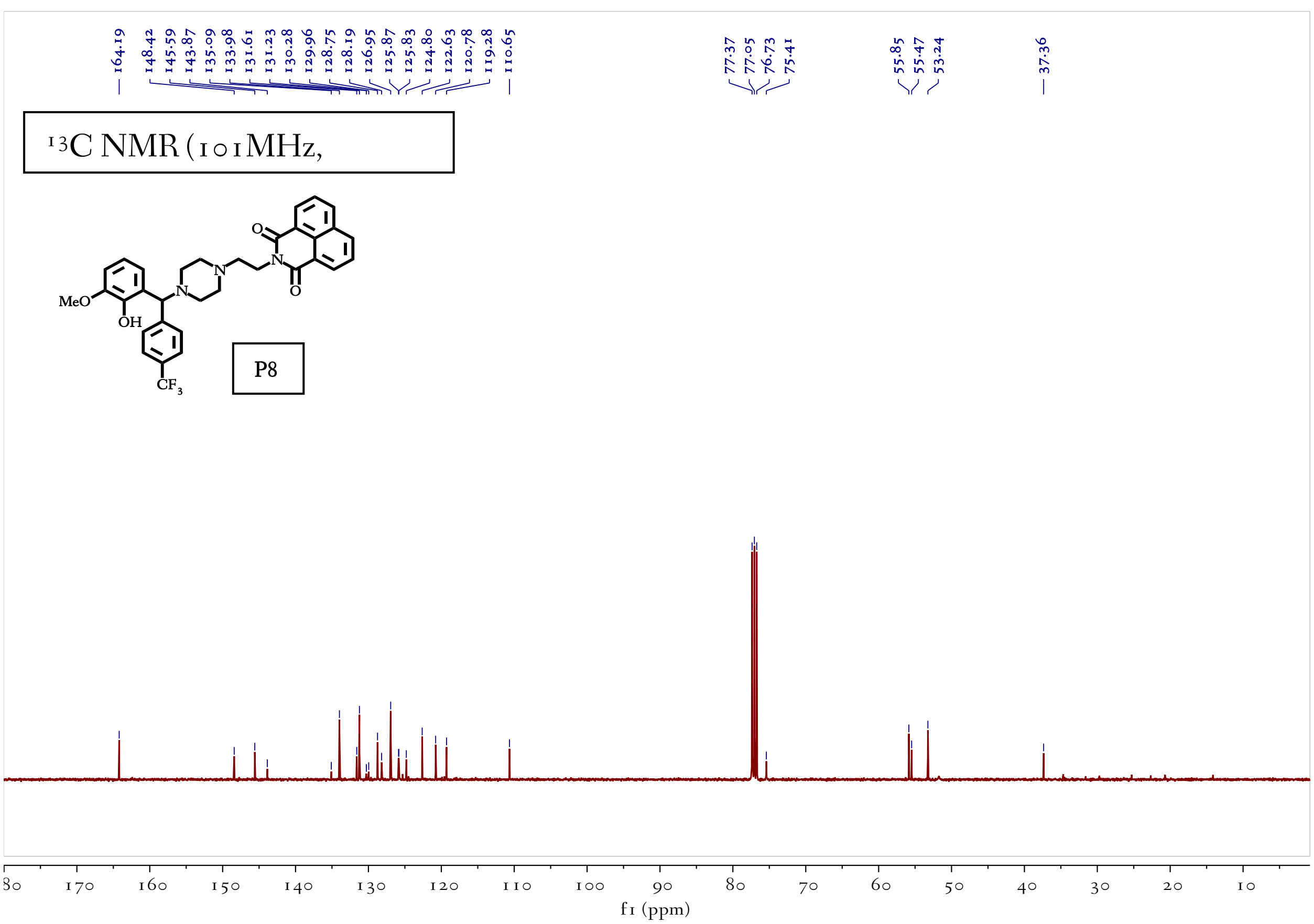

13C NMR (101MHz,
MeO
OH
CF3
N
O
P8
164.19
148.42 145.59 143.87 135.09 133.98 131.61 131.23 130.28 129.96 128.75 128.19 126.95 125.87 125.83 124.80 122.63 120.78 119.28 110.65
77.37 77.05 76.73 75.41
55.85 55.47 53.24
37.36
f1 (ppm)

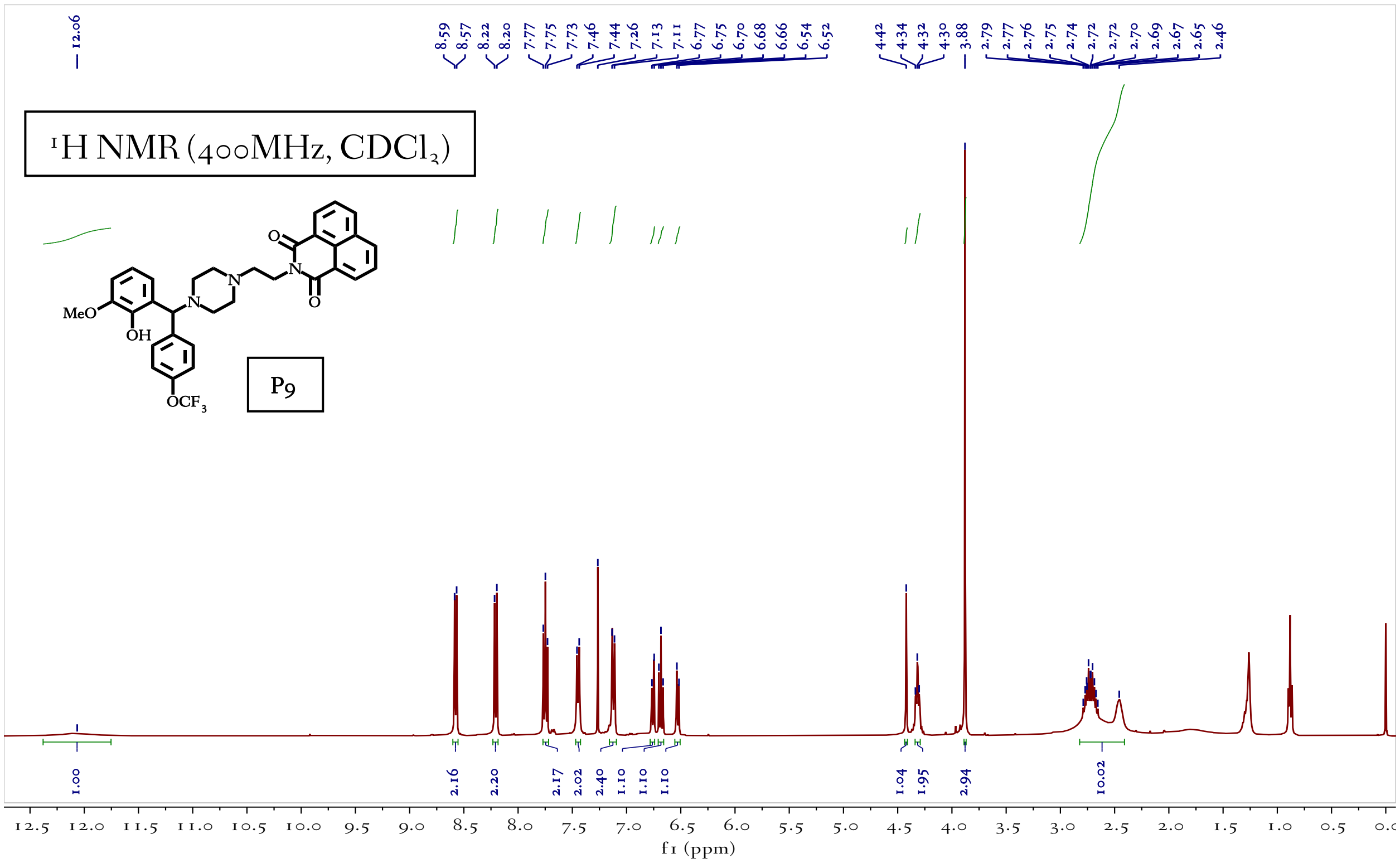

1H NMR (400MHz, CDCl3)
P9
MeO
OH
OCF3
12.06
8.59 8.57 8.22 8.20 7.77 7.75 7.73 7.46 7.44 7.26 7.13 7.11 6.77 6.75 6.70 6.68 6.66 6.54 6.52
4.42 4.34 4.32 4.30 3.88 2.79 2.77 2.76 2.75 2.74 2.72 2.72 2.70 2.69 2.67 2.65 2.46
1.00 2.16 2.20 2.17 2.02 2.40 1.10 1.10 1.10 1.04 1.95 2.94 10.02
f1 (ppm)

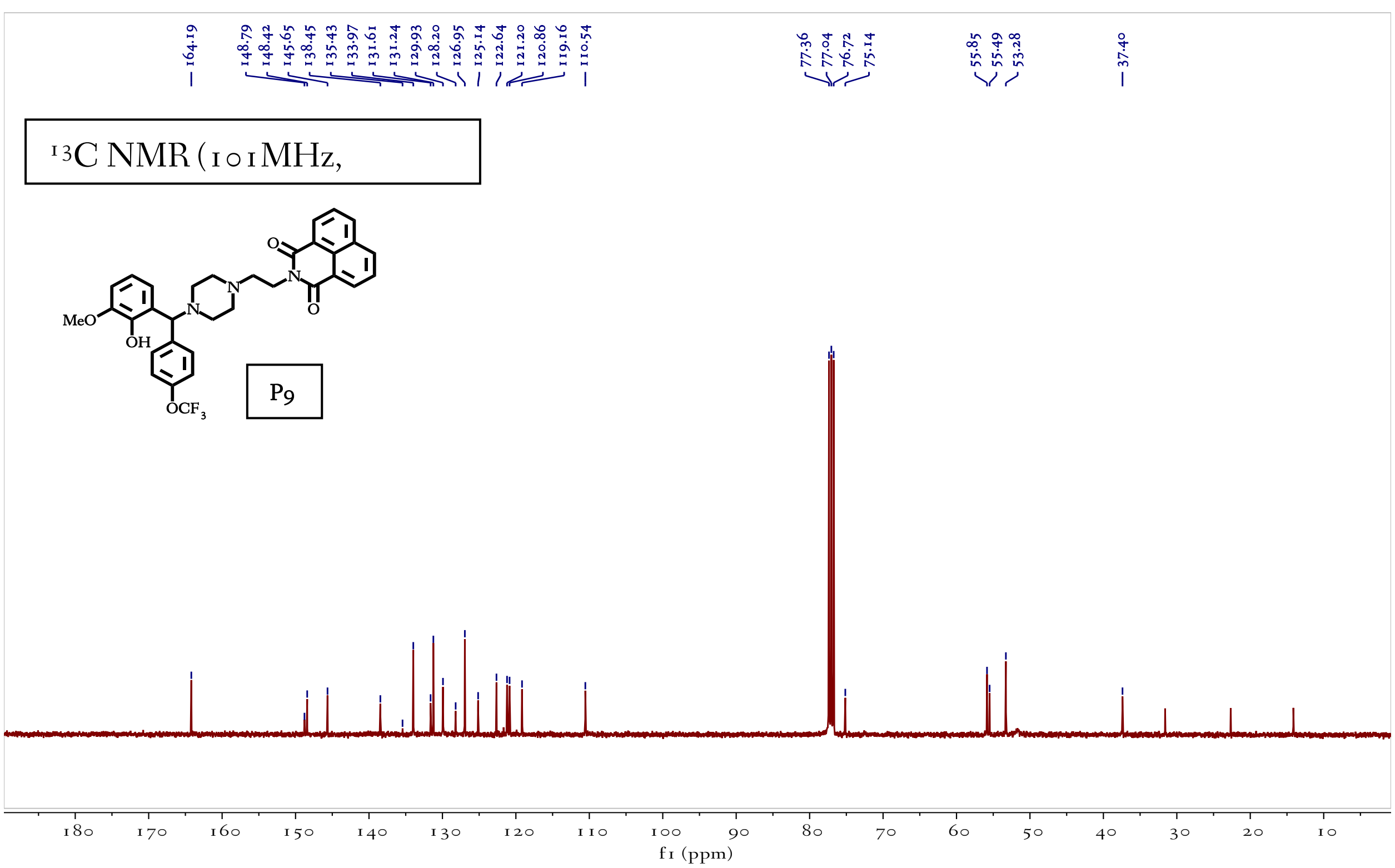

13C NMR (101MHz,
P9
MeO
OH
OCF3
164.19
148.79 148.42 145.65 138.45 135.43 133.97 131.61 131.24 129.93 128.20 126.95 125.14 122.64 121.20 120.86 119.16 110.54
77.36 77.04 76.72 75.14
55.85 55.49 53.28
37.40
f1 (ppm)

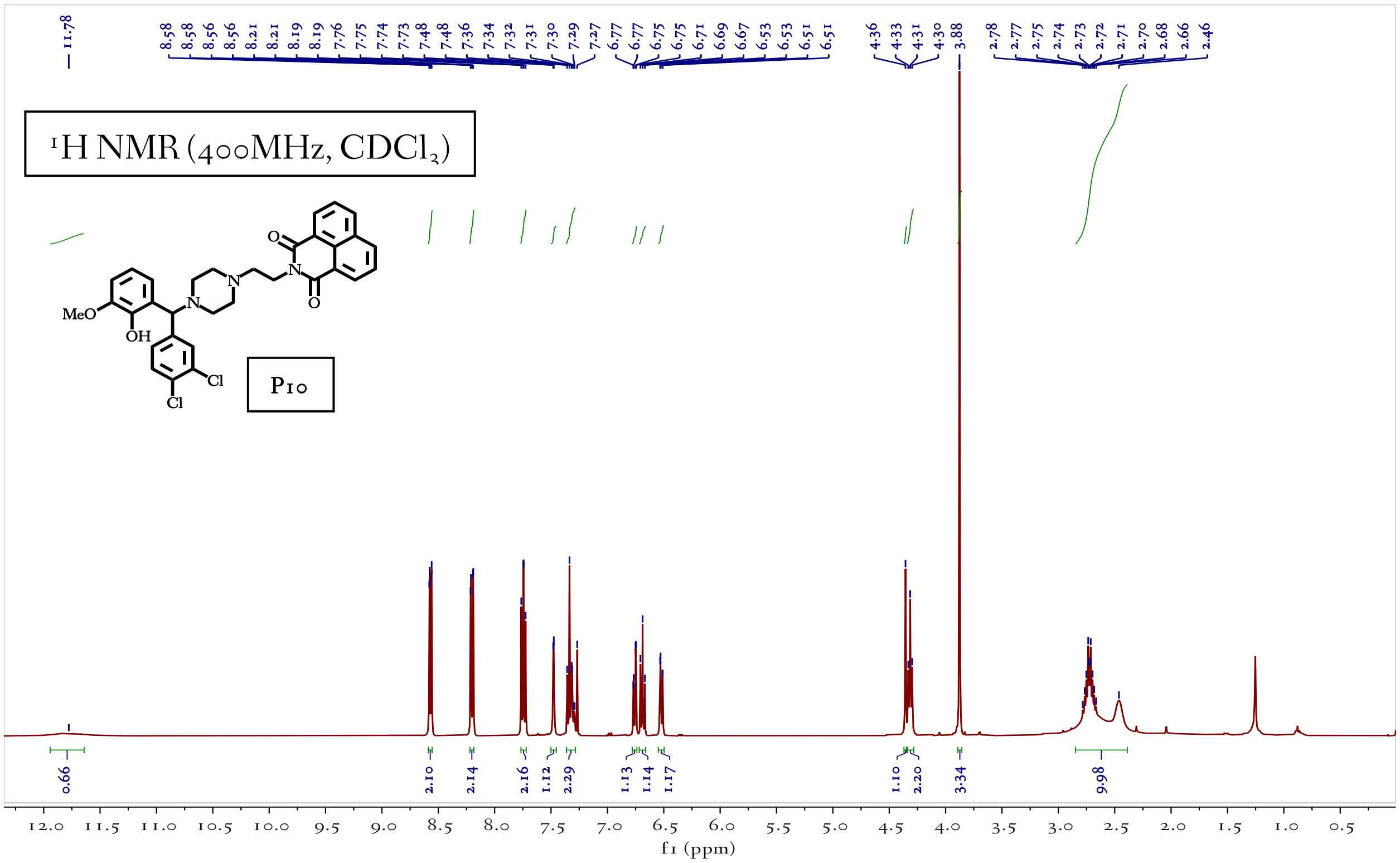

1H NMR (400MHz, CDCl3)
P10
MeO
OH
Cl
Cl
O
N
N
N
O
11.78
8.58 8.58 8.56 8.56 8.21 8.21 8.19 8.19 7.76 7.75 7.74 7.73 7.48 7.48 7.36 7.34 7.32 7.31 7.30 7.29 7.27 6.77 6.77 6.75 6.75 6.71 6.69 6.67 6.53 6.53 6.51 6.51
4.36 4.33 4.31 4.30 3.88
2.78 2.77 2.75 2.74 2.73 2.72 2.71 2.70 2.68 2.66 2.46
0.66 2.10 2.14 2.16 1.12 2.29 1.13 1.14 1.17 1.10 2.20 3.34 9.98
f1 (ppm)

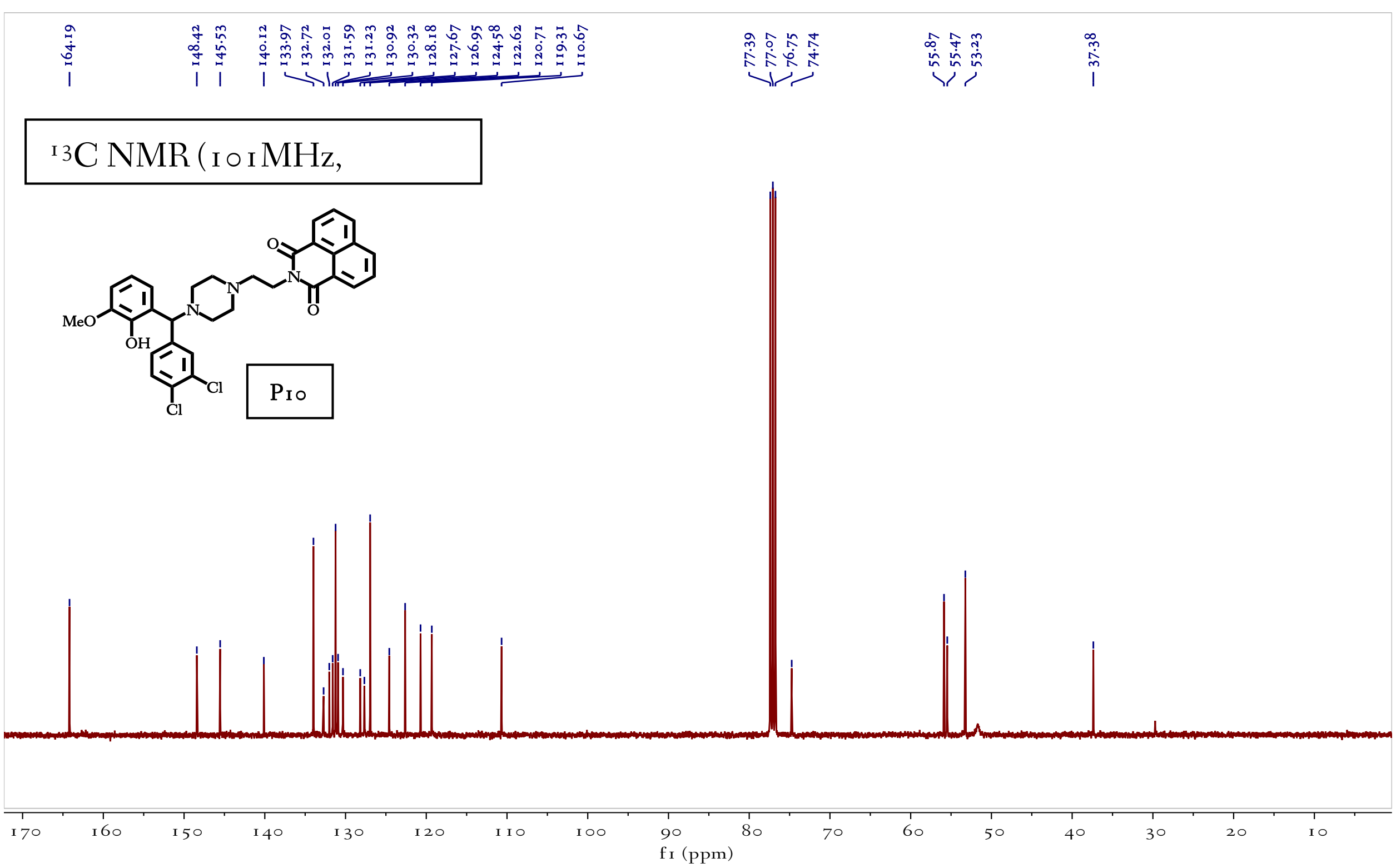

13C NMR (101MHz,
P10
MeO
OH
Cl
Cl
O
N
N
N
O
164.19
148.42 145.53
140.12 133.97 132.72 132.01 131.59 131.23 130.92 130.32 128.18 127.67 126.95 124.58 122.62 120.71 119.31 110.67
77.39 77.07 76.75 74.74
55.87 55.47 53.23
37.38
f1 (ppm)

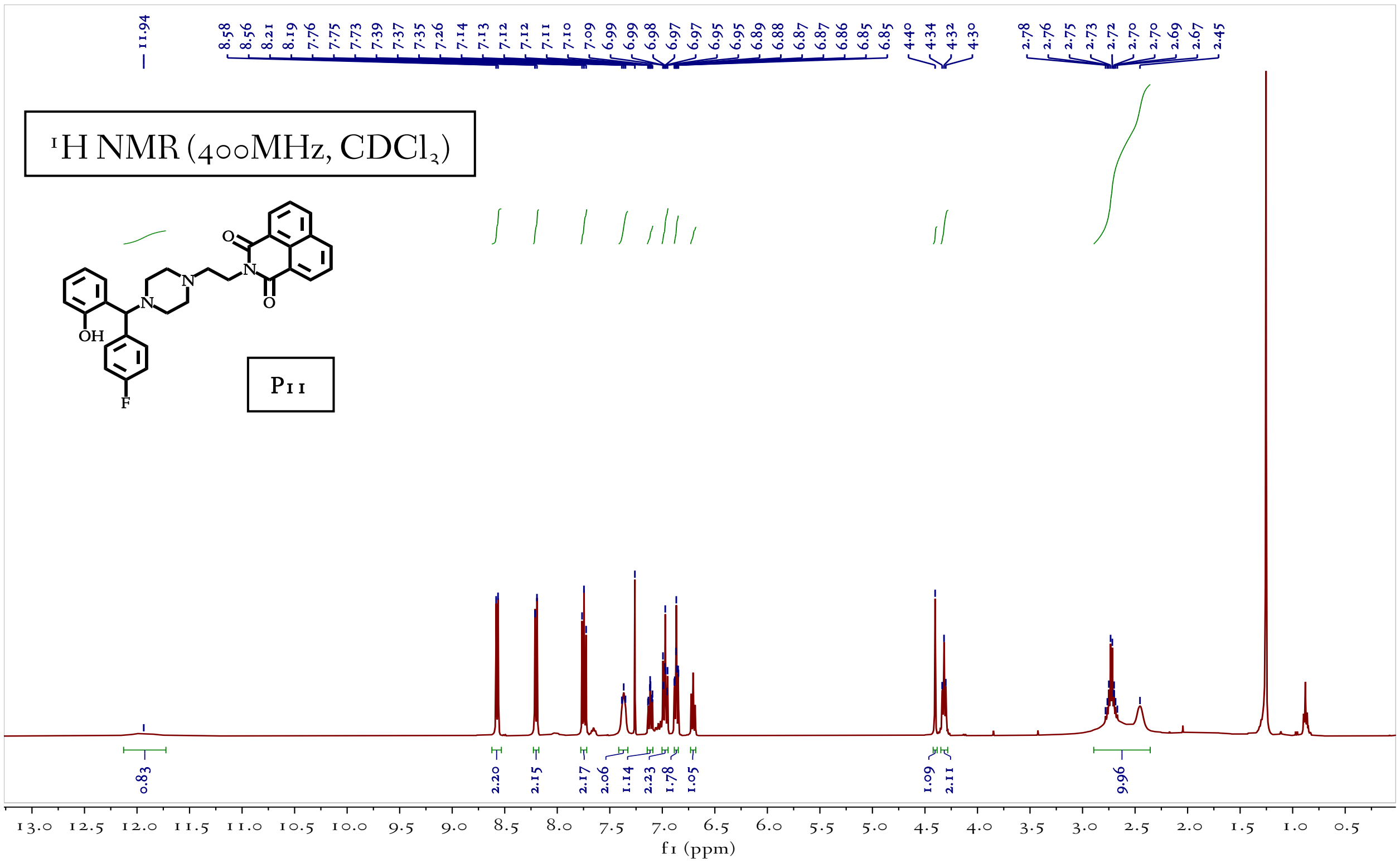
1H NMR (400MHz, CDCl3)
P11
f1 (ppm)

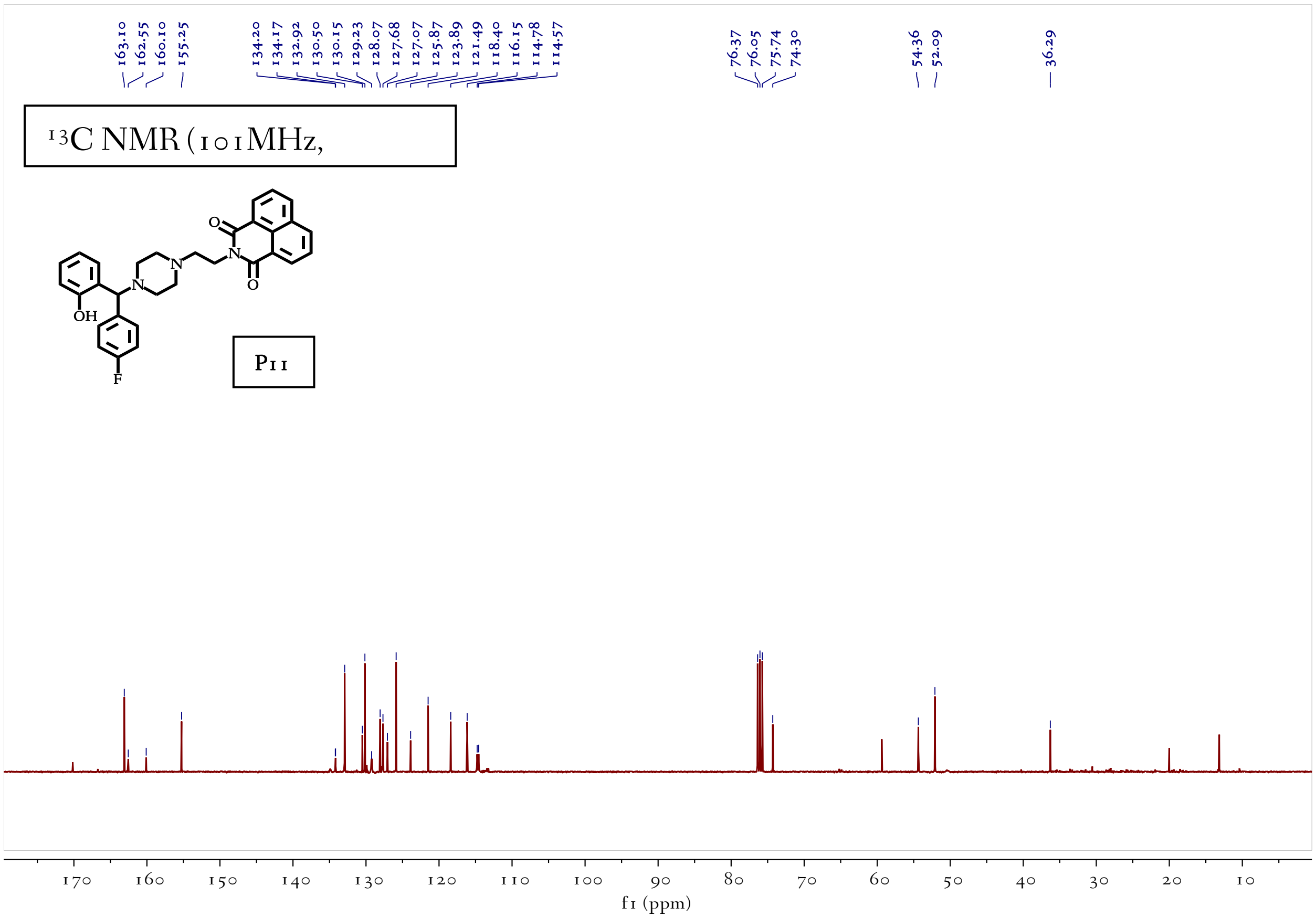

163.10
162.55
160.10
155.25
134.20
134.17
132.92
130.50
130.15
129.23
128.07
127.68
127.07
125.87
123.89
121.49
118.40
116.15
114.78
114.57
76.37
76.05
75.74
74.30
54.36
52.09
36.29
13C NMR (101MHz,
O
N
N
N
O
OH
F
P11
170
160
150
140
130
120
110
100
90
80
70
60
50
40
30
20
10
f1 (ppm)

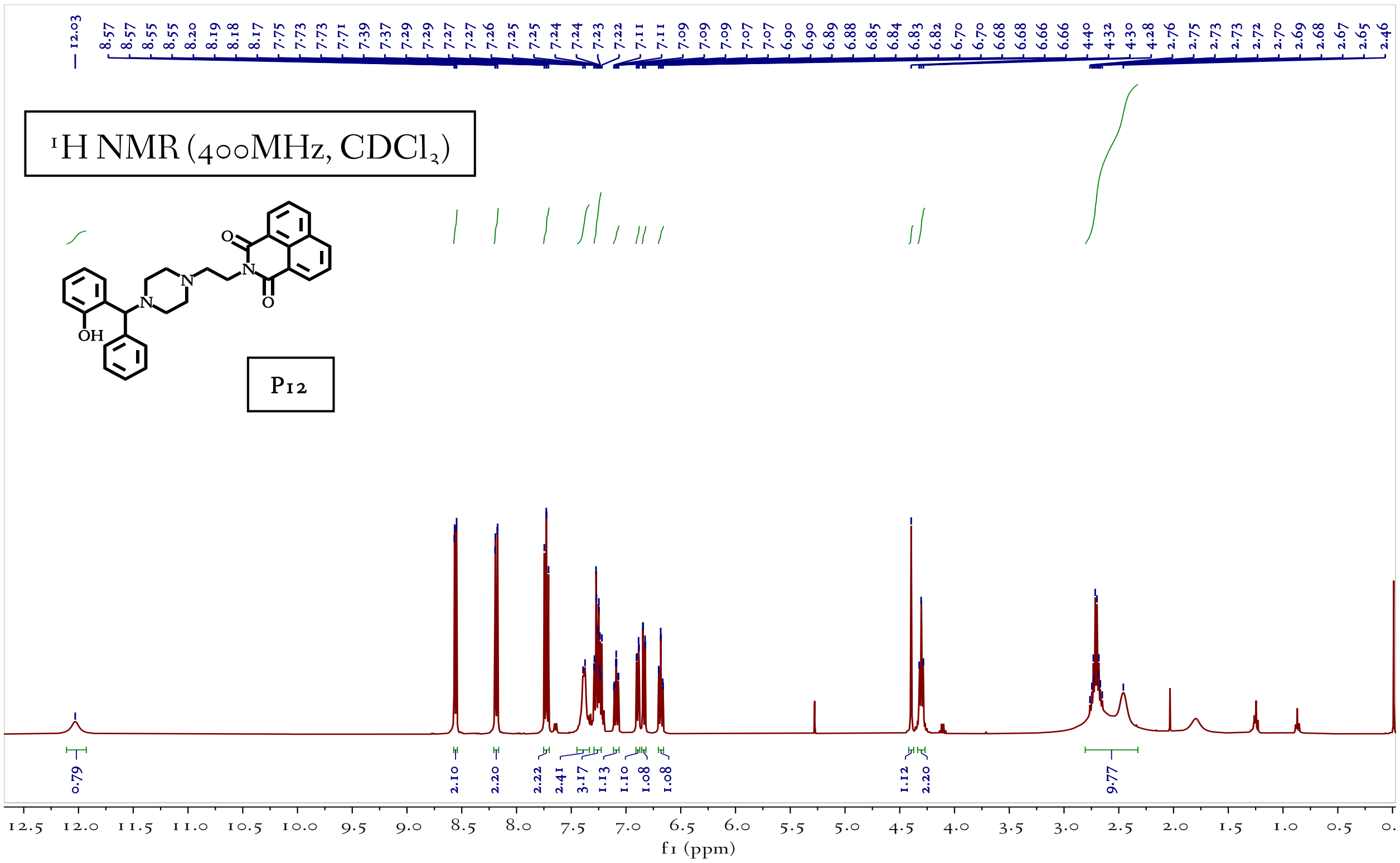
12.03
8.57 8.57 8.55 8.55 8.20 8.19 8.18 8.17 7.75 7.73 7.73 7.71 7.39 7.37 7.29 7.29 7.27 7.27 7.26 7.25 7.25 7.24 7.24 7.23 7.22 7.11 7.11 7.09 7.09 7.09 7.07 7.07 6.90 6.90 6.89 6.88 6.85 6.84 6.83 6.82 6.70 6.70 6.68 6.68 6.66 6.66 4.40 4.32 4.30 4.28 2.76 2.75 2.73 2.73 2.72 2.70 2.69 2.68 2.67 2.65 2.46
1H NMR (400MHz, CDCl3)
O
N
N
N
O
OH
P12
0.79
2.10
2.20
2.22
2.41
3.17
1.13
1.10
1.08
1.08
1.12
2.20
9.77
12.5 12.0 11.5 11.0 10.5 10.0 9.5 9.0 8.5 8.0 7.5 7.0 6.5 6.0 5.5 5.0 4.5 4.0 3.5 3.0 2.5 2.0 1.5 1.0 0.5 0.
f1 (ppm)

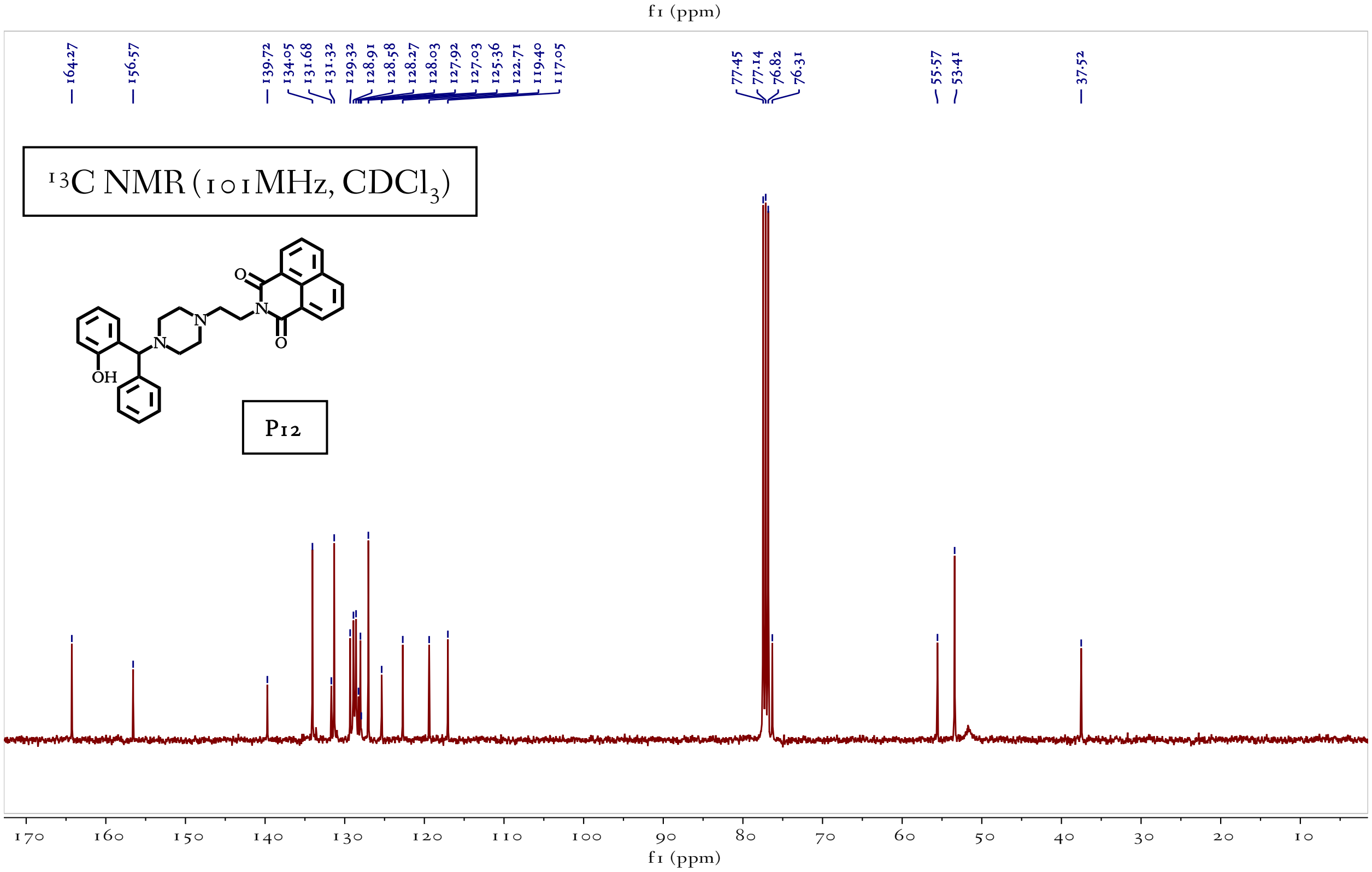
164.27
156.57
139.72 134.05 131.68 131.32 129.32 128.91 128.58 128.27 128.03 127.92 127.03 125.36 122.71 119.40 117.05
77.45 77.14 76.82 76.31
55.57
53.41
37.52
13C NMR (101MHz, CDCl3)
O
N
N
N
O
OH
P12
170 160 150 140 130 120 110 100 90 80 70 60 50 40 30 20 10
f1 (ppm)

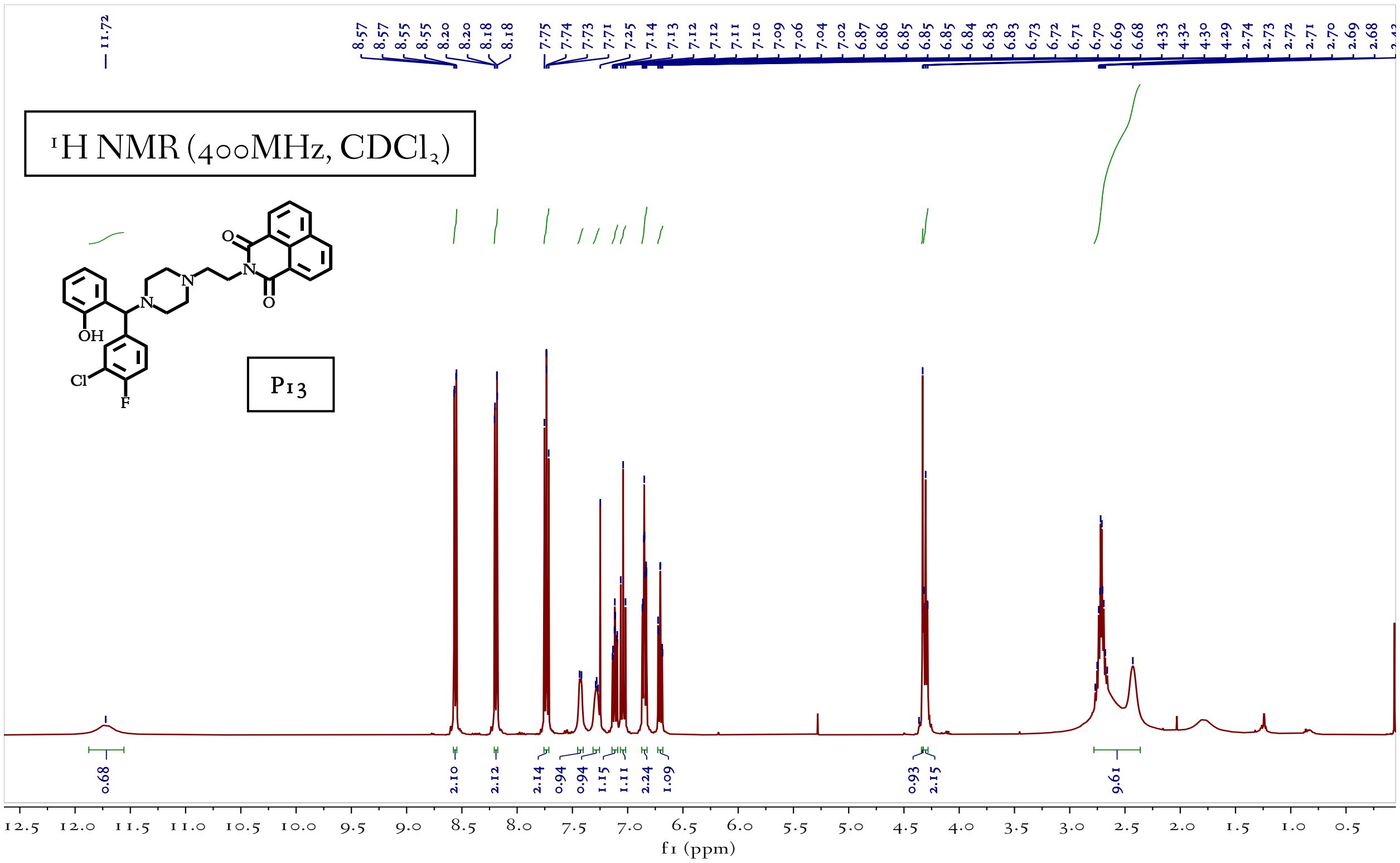
1H NMR (400MHz, CDCl3)
P13
f1 (ppm)

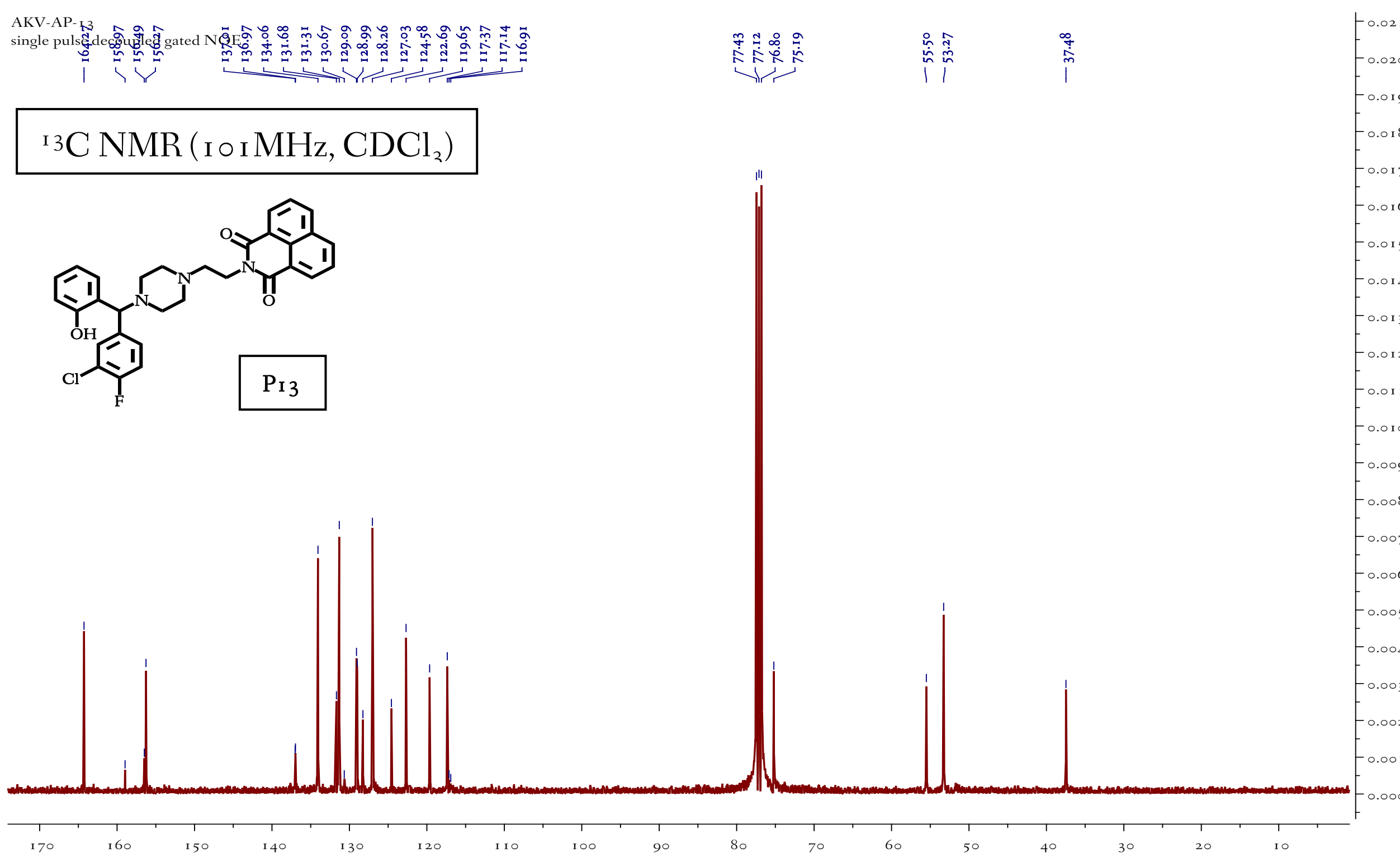
AKV-AP-13
single pulse decoupled gated NOE
13C NMR (101MHz, CDCl3)
P13

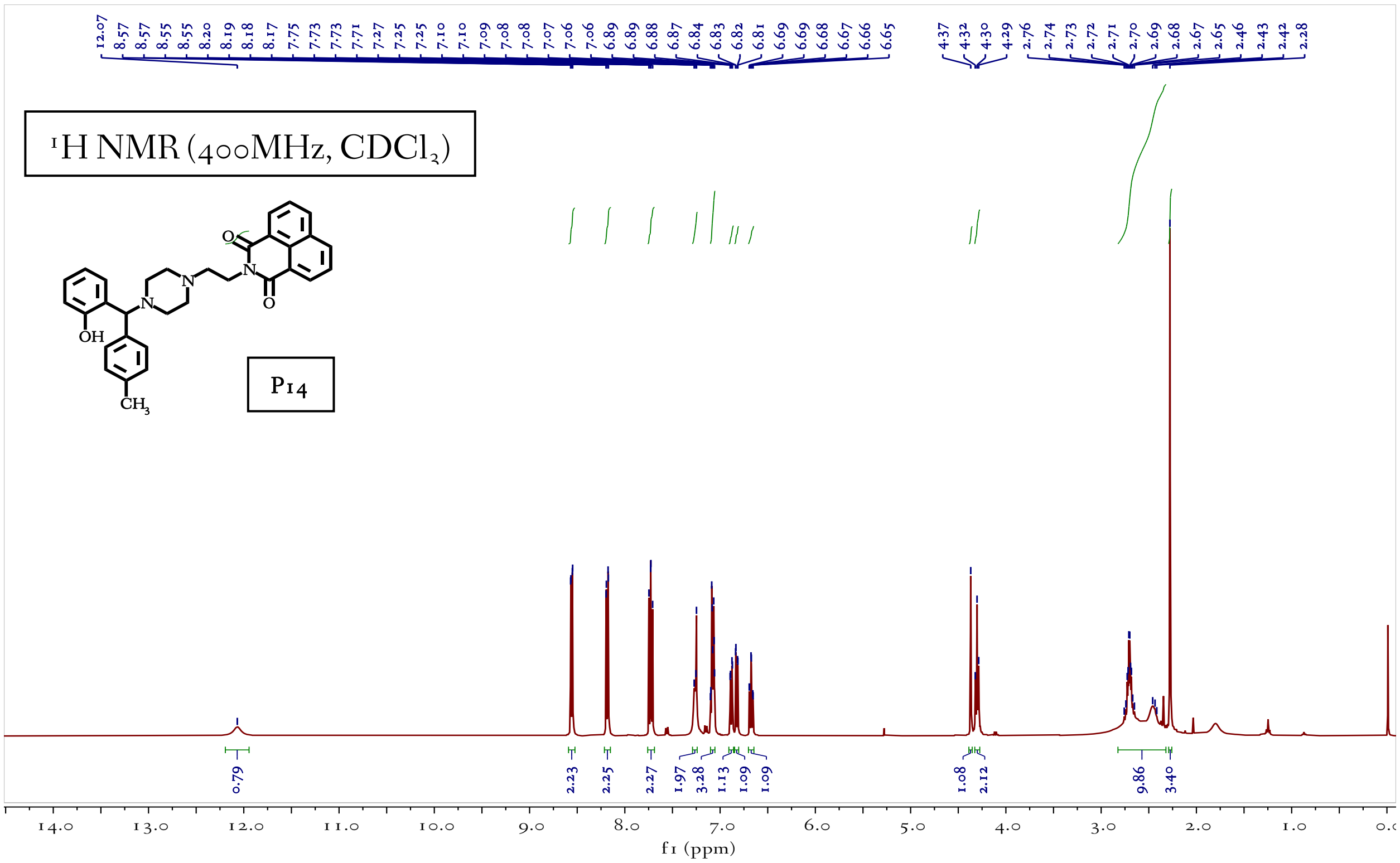
12.07 8.57 8.57 8.55 8.55 8.20 8.19 8.18 8.17 7.75 7.73 7.73 7.71 7.27 7.25 7.25 7.10 7.09 7.08 7.08 7.07 7.06 7.06 6.89 6.88 6.87 6.84 6.83 6.82 6.81 6.69 6.69 6.68 6.67 6.66 6.65 4.37 4.32 4.30 4.29 2.76 2.74 2.73 2.72 2.71 2.70 2.69 2.68 2.67 2.65 2.46 2.43 2.42 2.28
1H NMR (400MHz, CDCl3)
O
N
N
N
O
OH
CH3
P14
0.79 2.23 2.25 2.27 1.97 3.28 1.13 1.09 1.09 1.08 2.12 9.86 3.40
14.0 13.0 12.0 11.0 10.0 9.0 8.0 7.0 6.0 5.0 4.0 3.0 2.0 1.0 0.0
f1 (ppm)

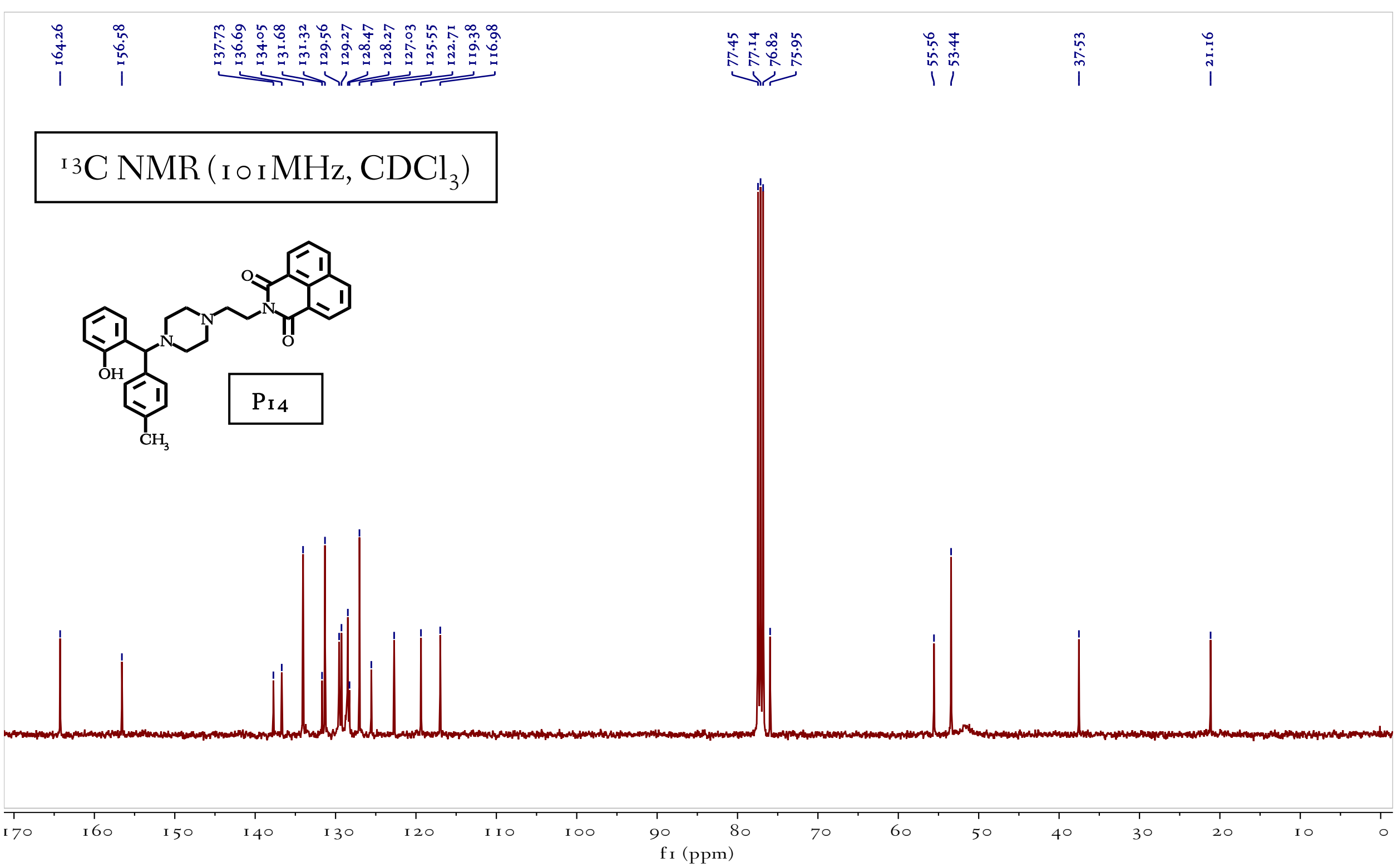
164.26 156.58 137.73 136.69 134.05 131.68 131.32 129.56 129.27 128.47 128.27 127.03 125.55 122.71 119.38 116.98 77.45 77.14 76.82 75.95 55.56 53.44 37.53 21.16
13C NMR (101MHz, CDCl3)
O
N
N
N
O
OH
CH3
P14
170 160 150 140 130 120 110 100 90 80 70 60 50 40 30 20 10 0
f1 (ppm)

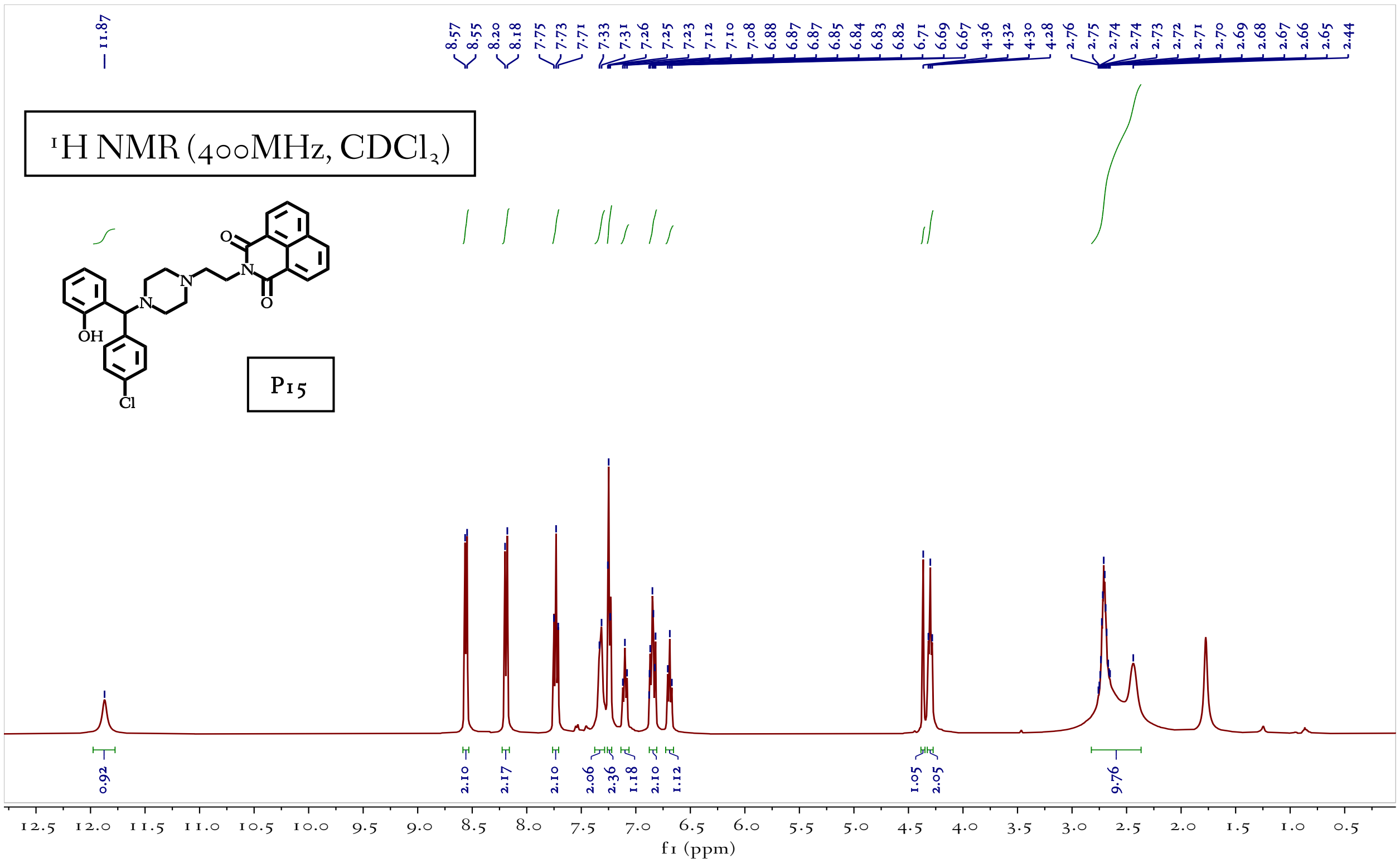

11.87
8.57
8.55
8.20
8.18
7.75
7.73
7.71
7.33
7.31
7.26
7.25
7.23
7.12
7.10
7.08
6.88
6.87
6.87
6.85
6.84
6.83
6.82
6.71
6.69
6.67
4.36
4.32
4.30
4.28
2.76
2.75
2.74
2.74
2.73
2.72
2.71
2.70
2.69
2.68
2.67
2.66
2.65
2.44
¹H NMR (400MHz, CDCl$_3$)
O
N
N
N
O
OH
Cl
P15
0.92
2.10
2.17
2.10
2.06
2.36
1.18
2.10
1.12
1.05
2.05
9.76
f1 (ppm)

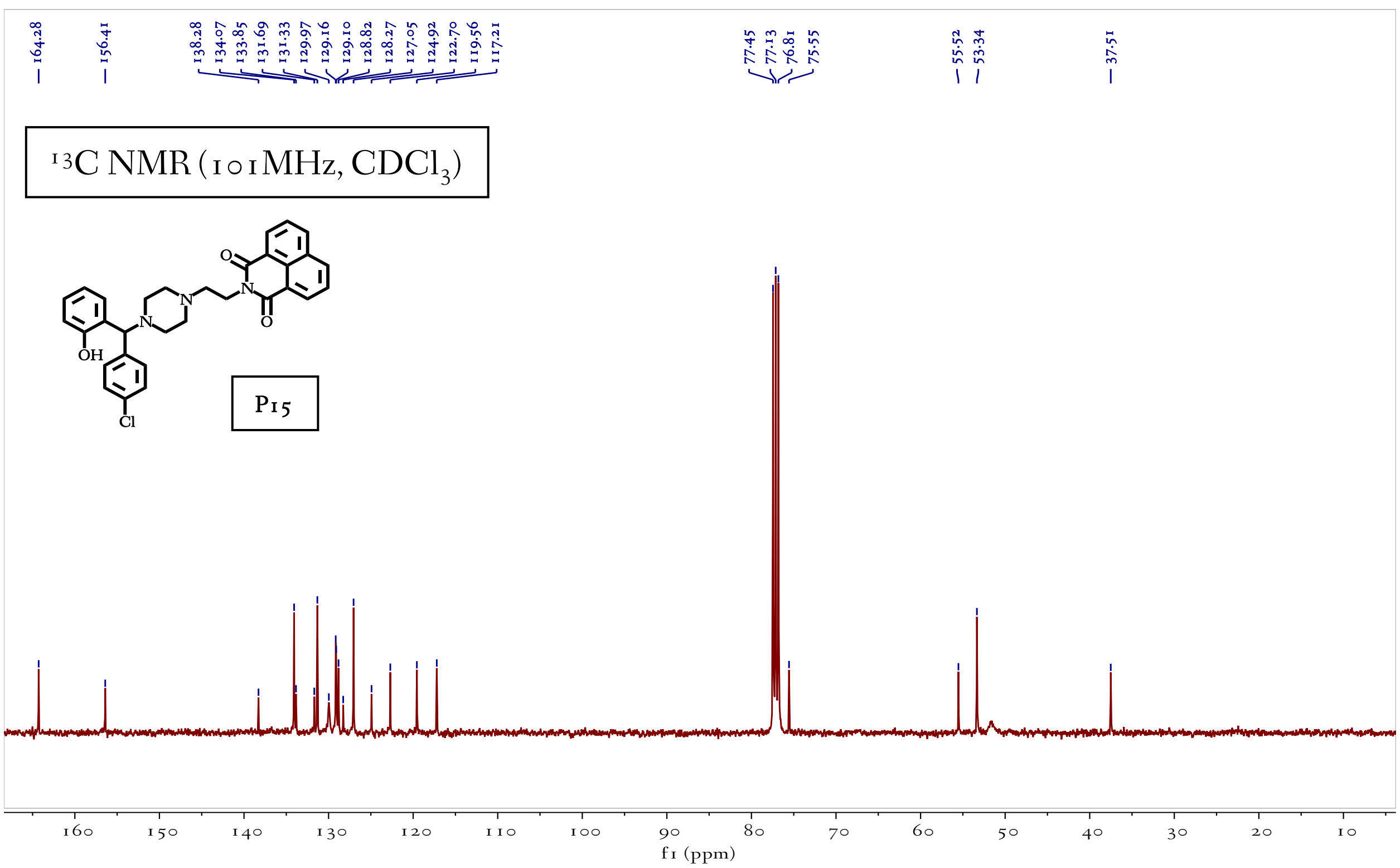

164.28
156.41
138.28
134.07
133.85
131.69
131.33
129.97
129.16
129.10
128.82
128.27
127.05
124.92
122.70
119.56
117.21
77.45
77.13
76.81
75.55
55.52
53.34
37.51
¹³C NMR (101MHz, CDCl$_3$)
O
N
N
N
O
OH
Cl
P15
f1 (ppm)

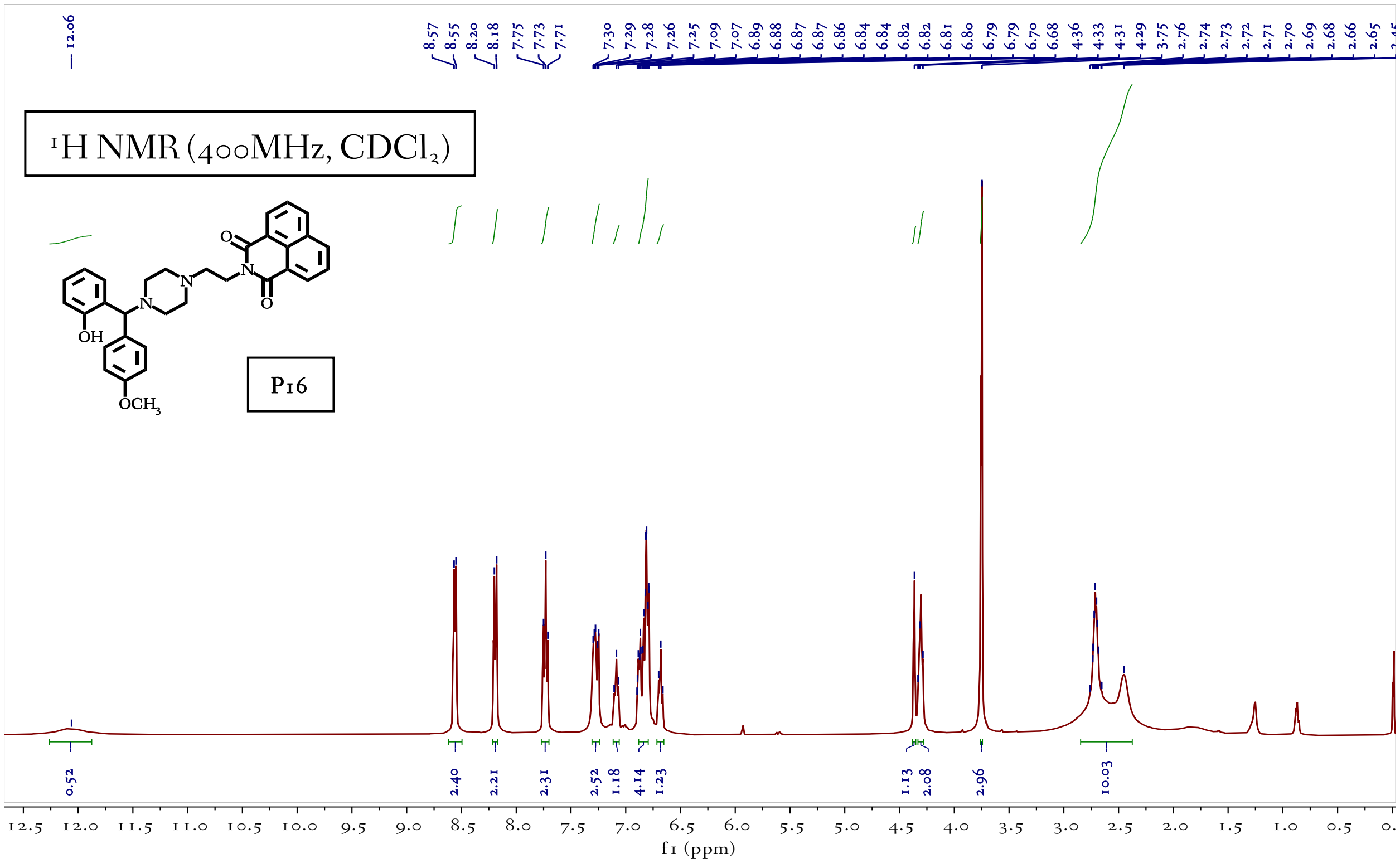

^{1}H NMR (400MHz, $CDCl_3$)
P16
OH
OCH_3
f1 (ppm)

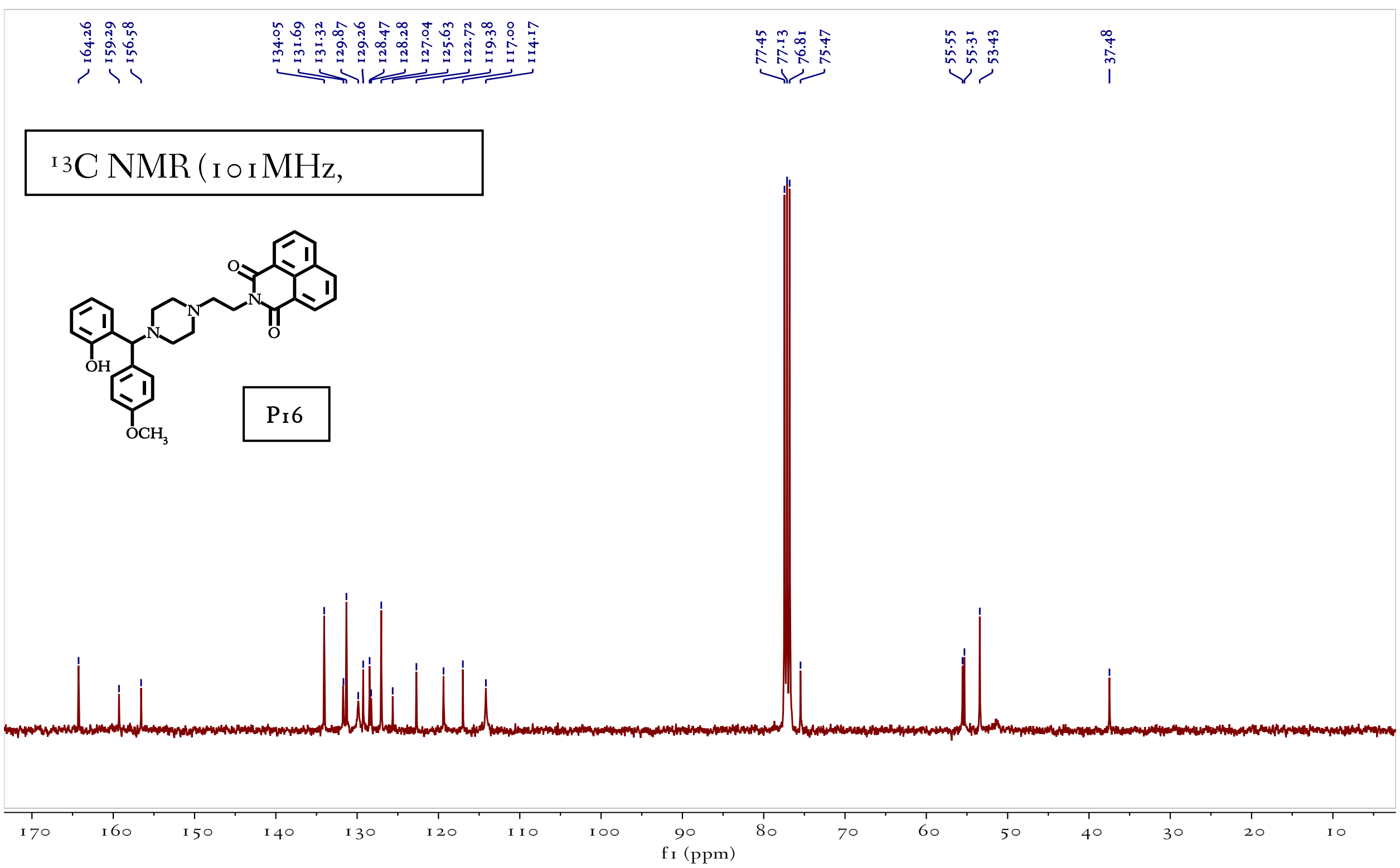

^{13}C NMR (101MHz,
P16
OH
OCH_3
f1 (ppm)

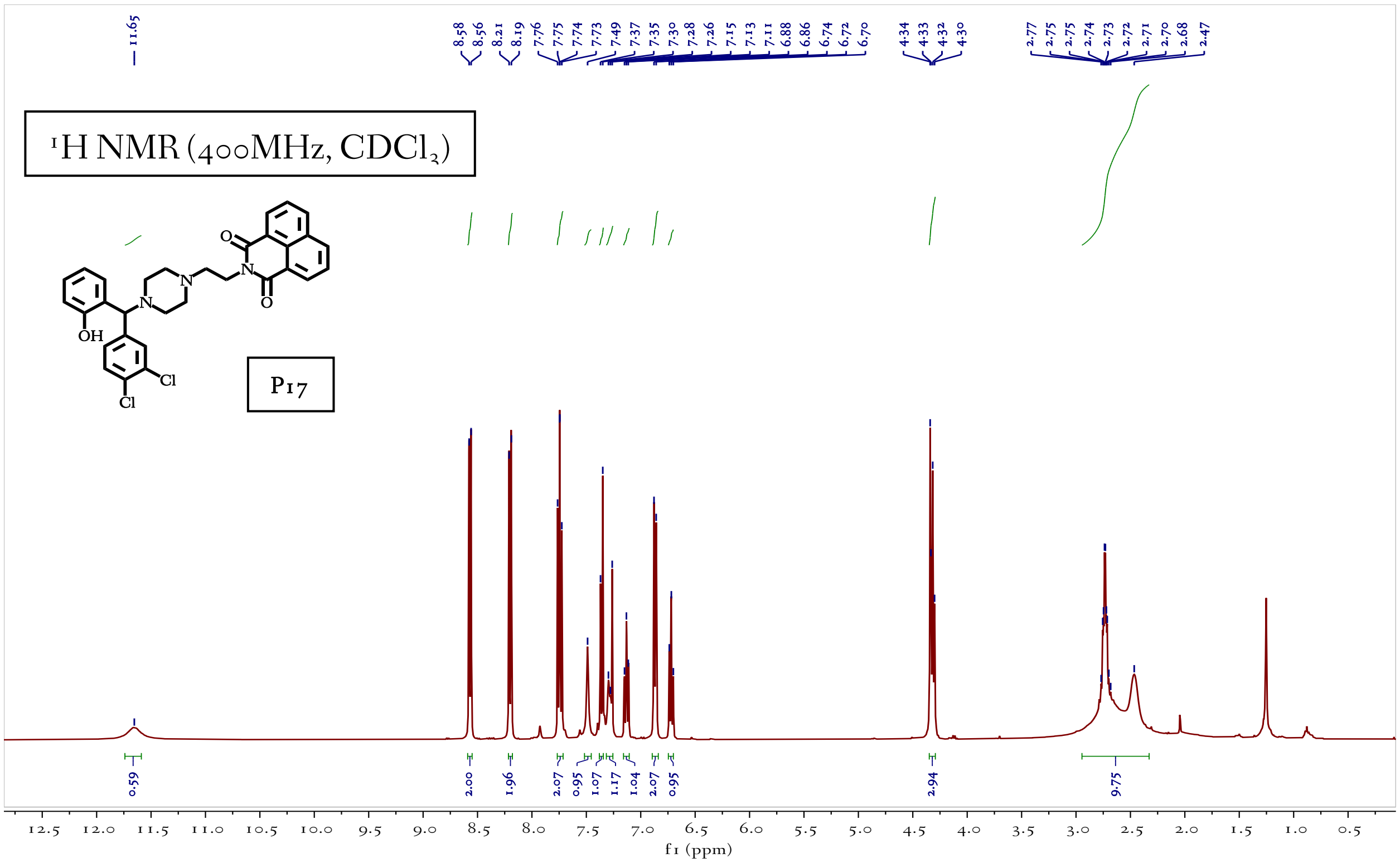

1H NMR (400MHz, CDCl3)
P17
OH
Cl
Cl
N
N
N
O
O
11.65
8.58
8.56
8.21
8.19
7.76
7.75
7.74
7.73
7.49
7.37
7.35
7.30
7.28
7.26
7.15
7.13
7.11
6.88
6.86
6.74
6.72
6.70
4.34
4.33
4.32
4.30
2.77
2.75
2.75
2.74
2.73
2.72
2.71
2.70
2.68
2.47
0.59
2.00
1.96
2.07
0.95
1.07
1.17
1.04
2.07
0.95
2.94
9.75
f1 (ppm)

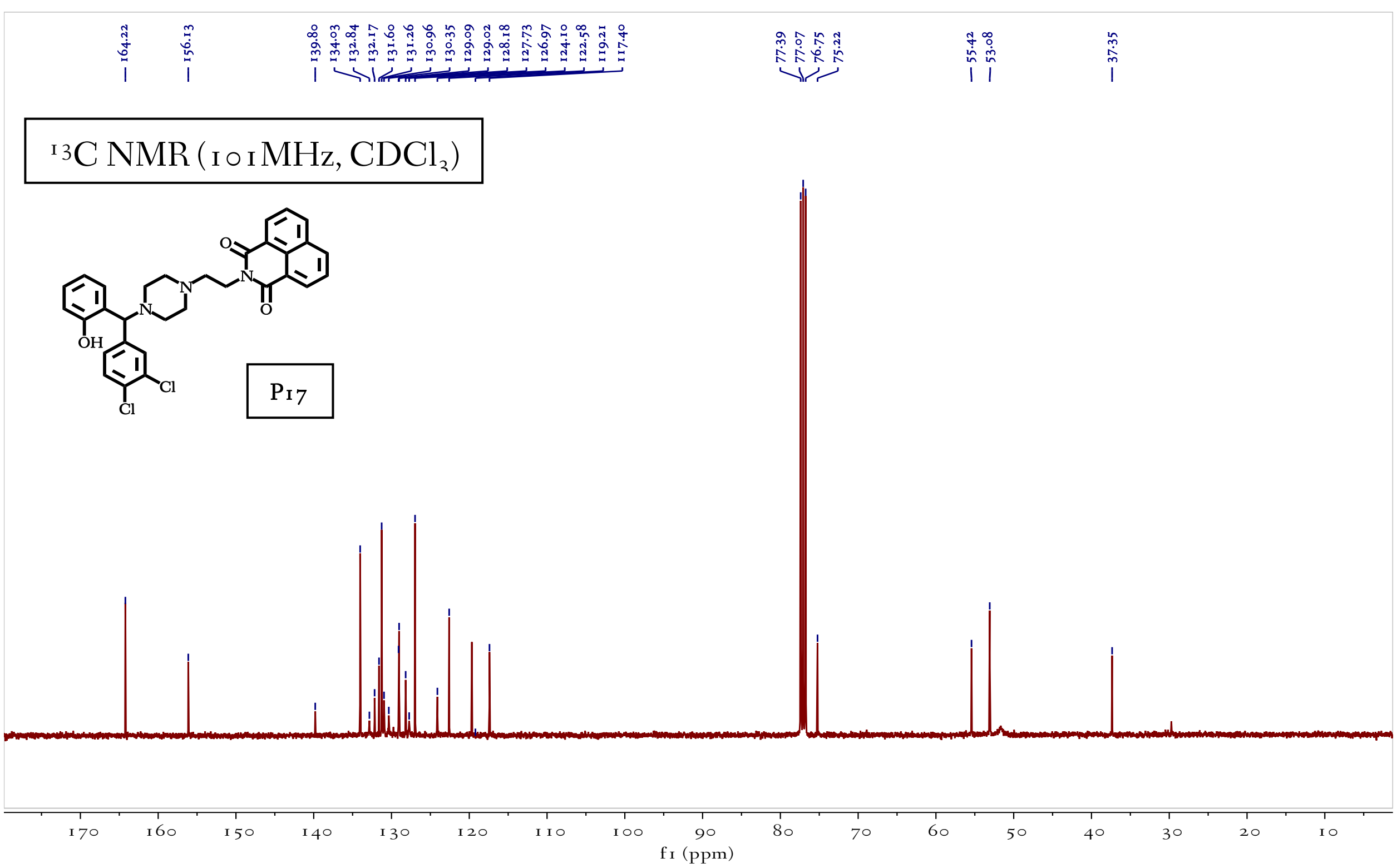

13C NMR (101MHz, CDCl3)
P17
OH
Cl
Cl
N
N
N
O
O
164.22
156.13
139.80
134.03
132.84
132.17
131.60
131.26
130.96
130.35
129.09
129.02
128.18
127.73
126.97
124.10
122.58
119.21
117.40
77.39
77.07
76.75
75.22
55.42
53.08
37.35
f1 (ppm)

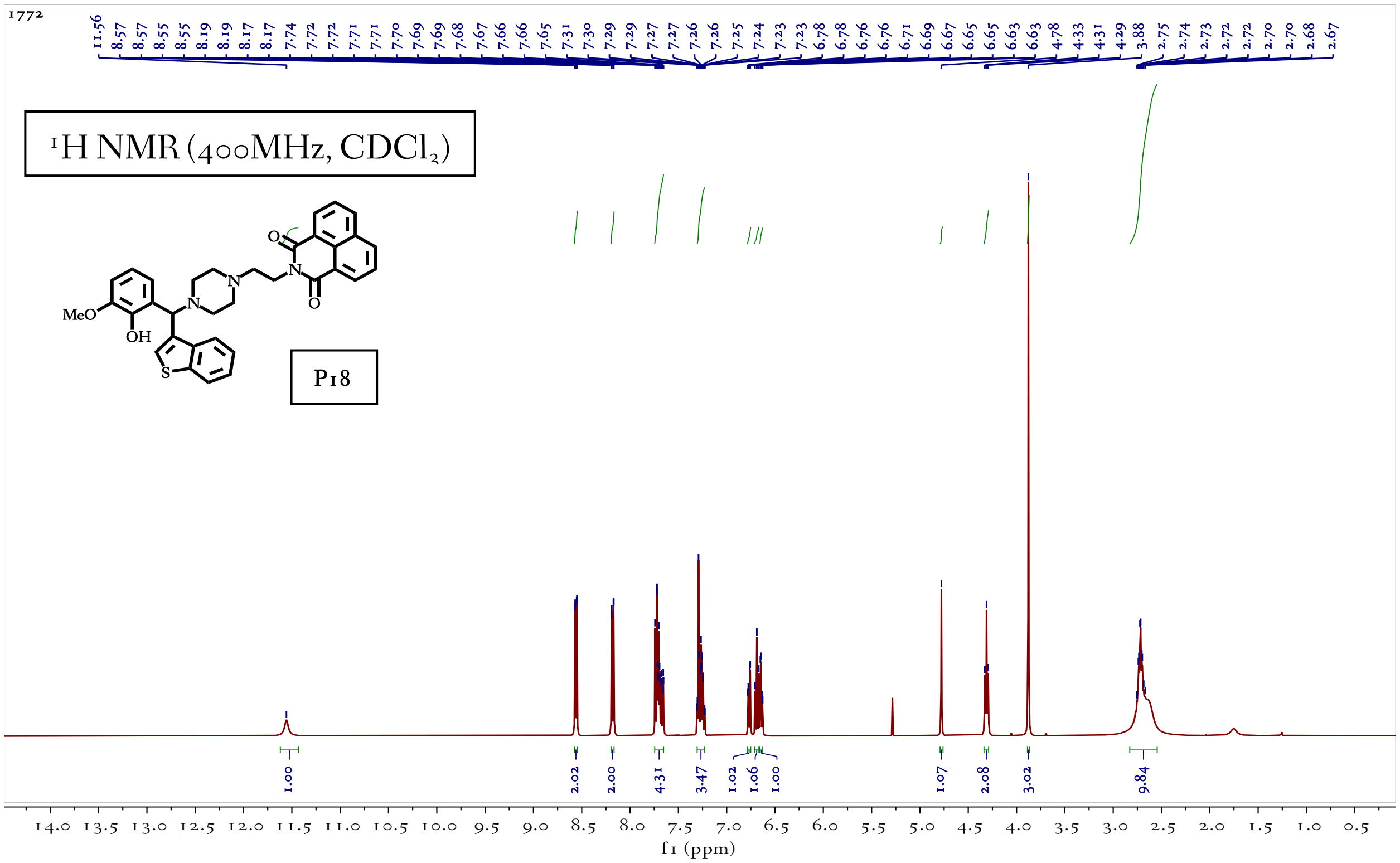
1772
11.56 8.57 8.57 8.55 8.55 8.19 8.19 8.17 8.17 7.74 7.72 7.72 7.71 7.71 7.70 7.69 7.69 7.68 7.67 7.67 7.66 7.66 7.65 7.31 7.30 7.29 7.29 7.27 7.27 7.26 7.26 7.25 7.24 7.23 7.23 6.78 6.78 6.76 6.76 6.71 6.69 6.67 6.65 6.65 6.63 6.63 6.63 4.78 4.33 4.31 4.29 3.88 2.75 2.74 2.73 2.72 2.72 2.70 2.68 2.67
¹H NMR (400MHz, CDCl₃)
MeO
OH
S
N
N
N
O
O
P18
1.00 2.02 2.00 4.31 3.47 1.02 1.06 1.00 1.07 2.08 3.02 9.84
14.0 13.5 13.0 12.5 12.0 11.5 11.0 10.5 10.0 9.5 9.0 8.5 8.0 7.5 7.0 6.5 6.0 5.5 5.0 4.5 4.0 3.5 3.0 2.5 2.0 1.5 1.0 0.5
f1 (ppm)

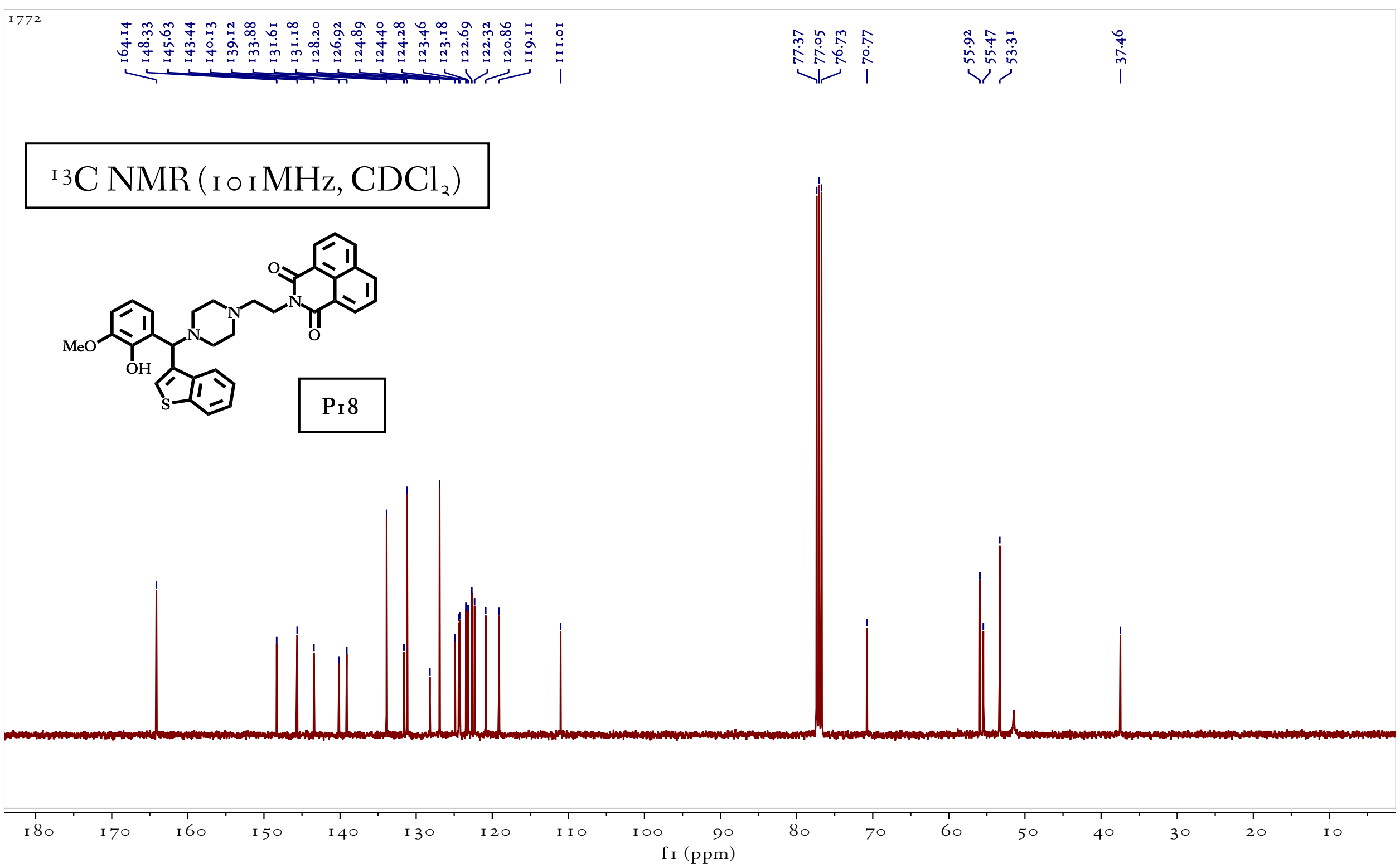
1772
164.14 148.33 145.63 143.44 140.13 139.12 133.88 131.61 131.18 128.20 126.92 124.89 124.40 124.28 123.46 123.18 122.69 122.32 120.86 119.11 111.01 77.37 77.05 76.73 70.77 55.92 55.47 53.31 37.46
¹³C NMR (101MHz, CDCl₃)
MeO
OH
S
N
N
N
O
O
P18
180 170 160 150 140 130 120 110 100 90 80 70 60 50 40 30 20 10
f1 (ppm)

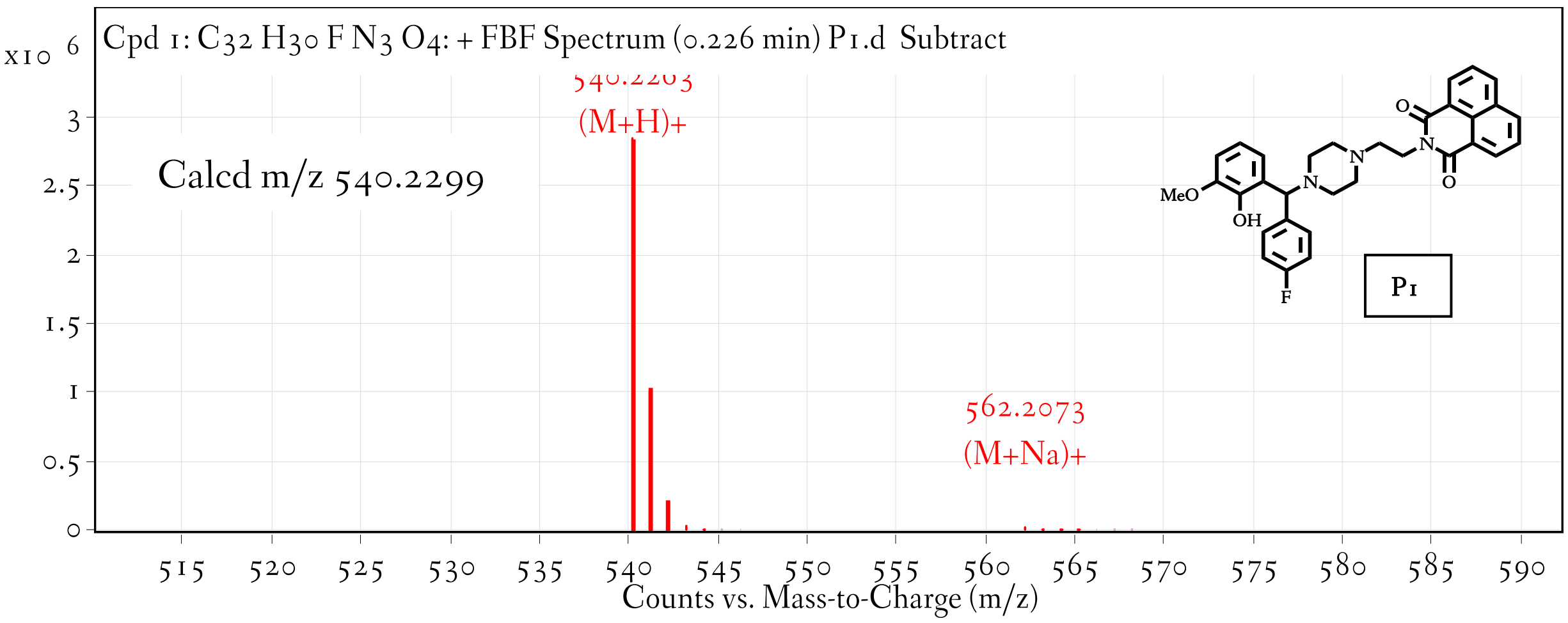
Cpd 1: C32 H30 F N3 O4: + FBF Spectrum (0.226 min) P1.d Subtract
x10 6
Calcd m/z 540.2299
(M+H)+
562.2073
(M+Na)+
MeO
OH
F
N
N
N
O
O
P1
Counts vs. Mass-to-Charge (m/z)

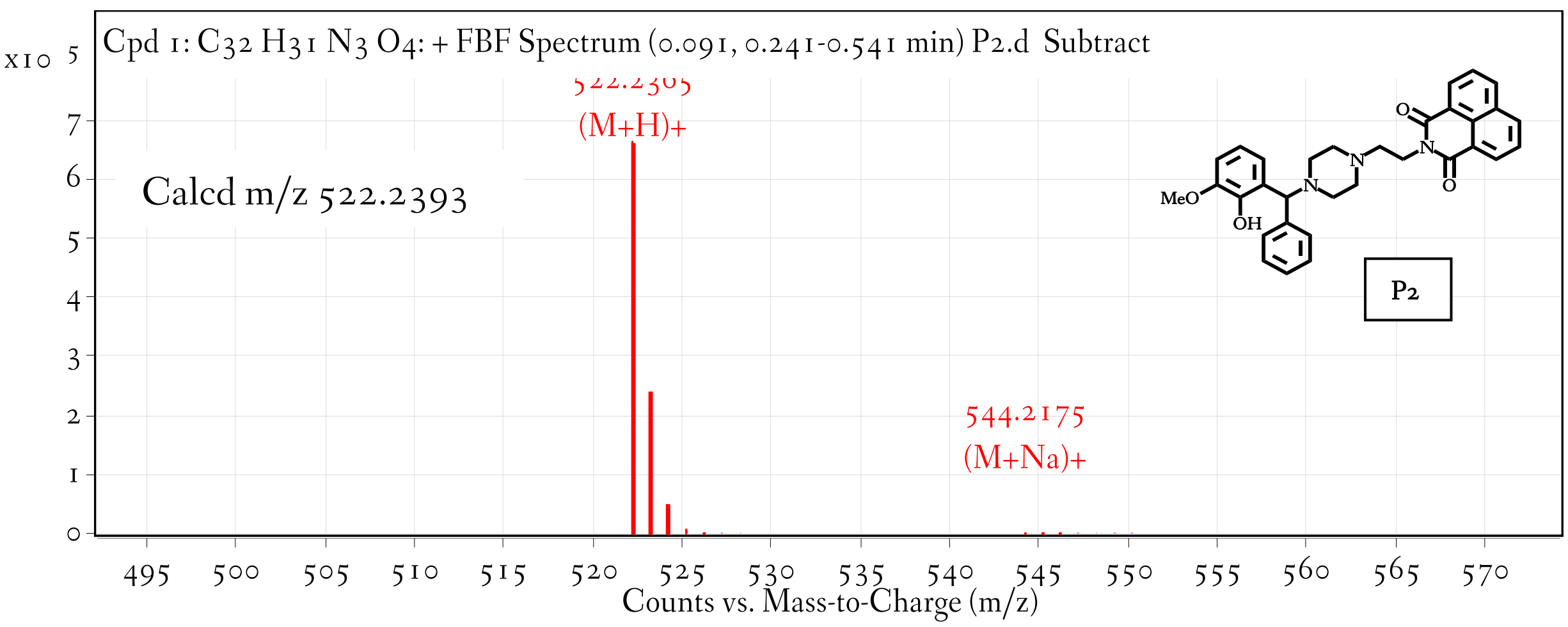
Cpd 1: C32 H31 N3 O4: + FBF Spectrum (0.091, 0.241-0.541 min) P2.d Subtract
x10 5
Calcd m/z 522.2393
(M+H)+
544.2175
(M+Na)+
MeO
OH
N
N
N
O
O
P2
Counts vs. Mass-to-Charge (m/z)

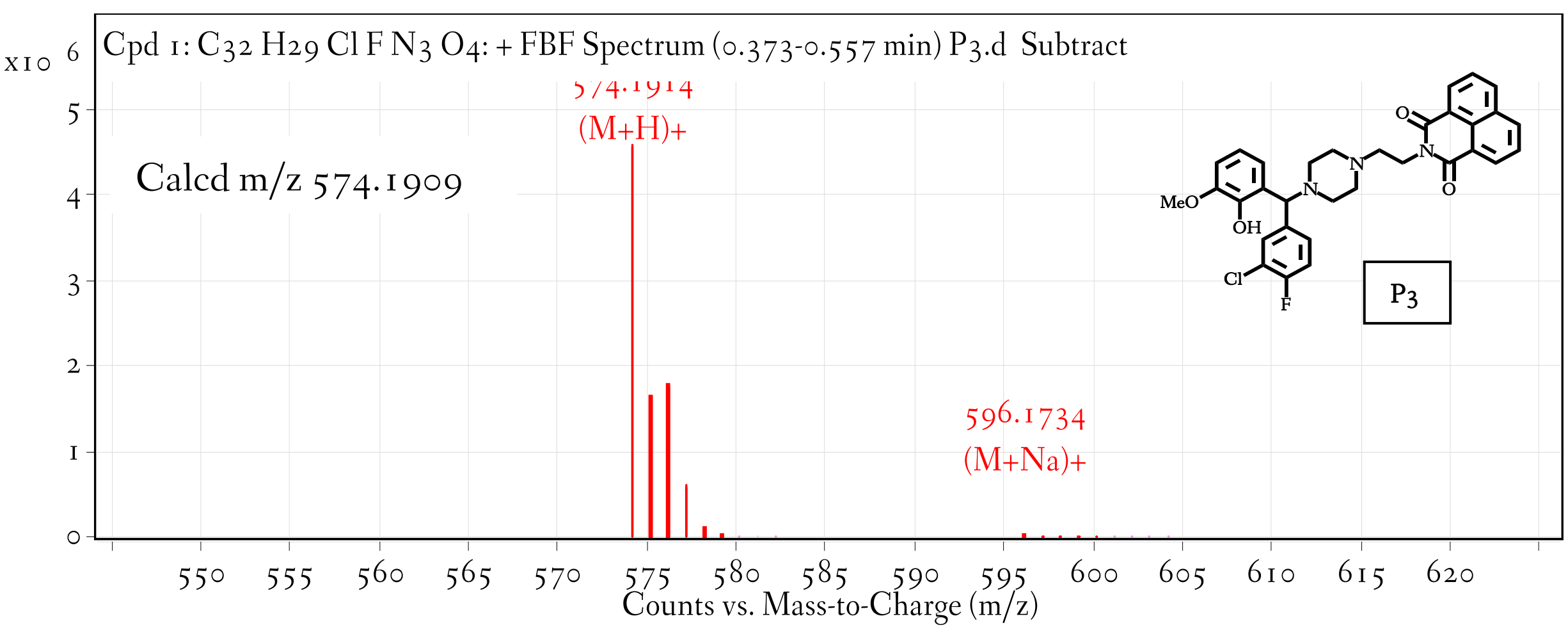
Cpd 1: C32 H29 Cl F N3 O4: + FBF Spectrum (0.373-0.557 min) P3.d Subtract
x10 6
Calcd m/z 574.1909
(M+H)+
596.1734
(M+Na)+
MeO
OH
Cl
F
N
N
N
O
O
P3
Counts vs. Mass-to-Charge (m/z)

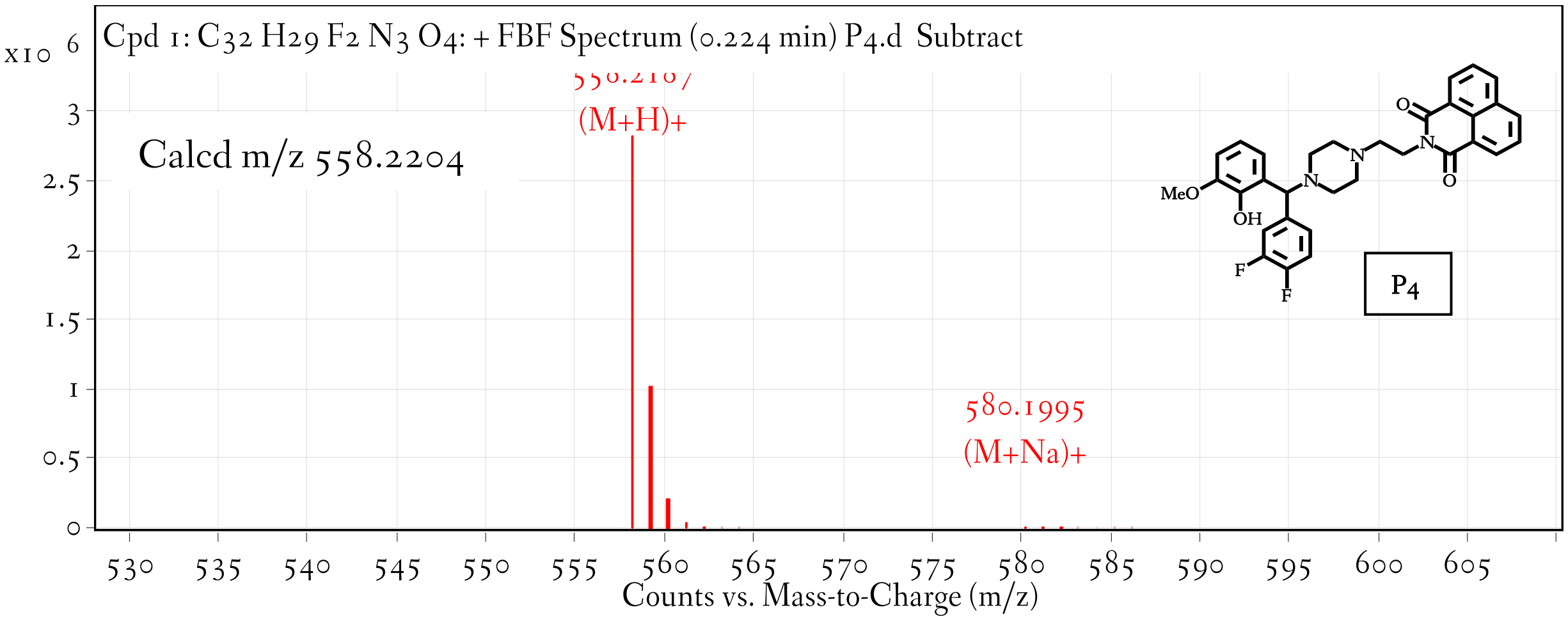
Cpd 1: C32 H29 F2 N3 O4: + FBF Spectrum (0.224 min) P4.d Subtract
x10 6
Calcd m/z 558.2204
(M+H)+
580.1995
(M+Na)+
MeO
OH
F
F
N
N
N
O
O
P4
Counts vs. Mass-to-Charge (m/z)

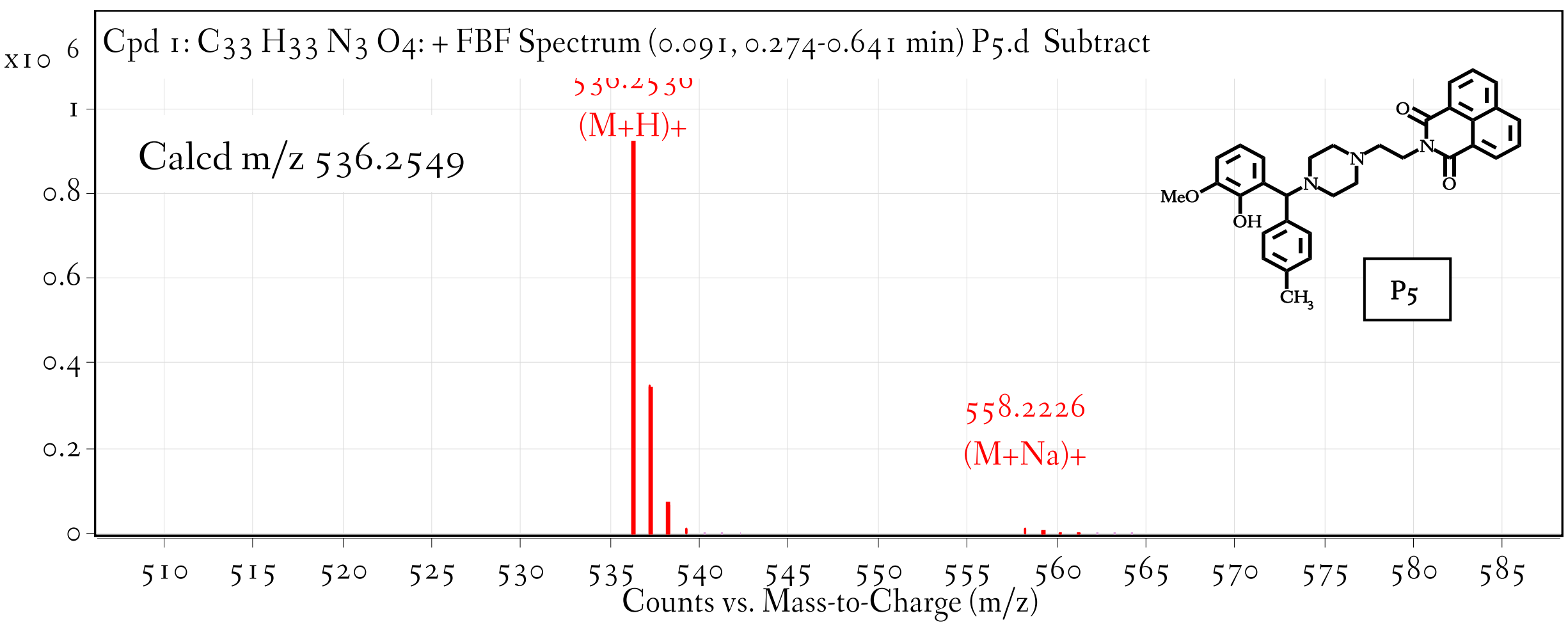
Cpd 1: C33 H33 N3 O4: + FBF Spectrum (0.091, 0.274-0.641 min) P5.d Subtract
x10 6
Calcd m/z 536.2549
(M+H)+
558.2226
(M+Na)+
MeO
OH
CH3
N
N
N
O
O
P5
Counts vs. Mass-to-Charge (m/z)

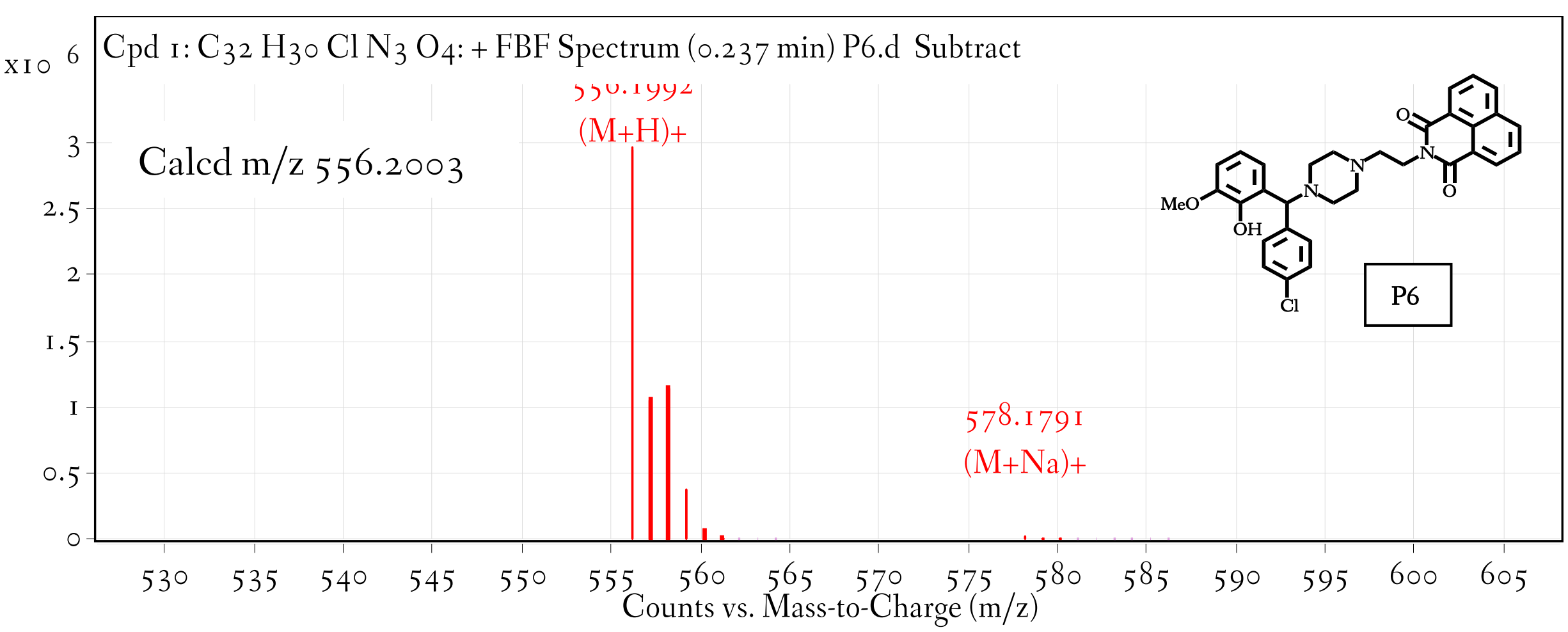
Cpd 1: C32 H30 Cl N3 O4: + FBF Spectrum (0.237 min) P6.d Subtract
x10 6
Calcd m/z 556.2003
(M+H)+
578.1791
(M+Na)+
MeO
OH
Cl
N
N
N
O
O
P6
Counts vs. Mass-to-Charge (m/z)

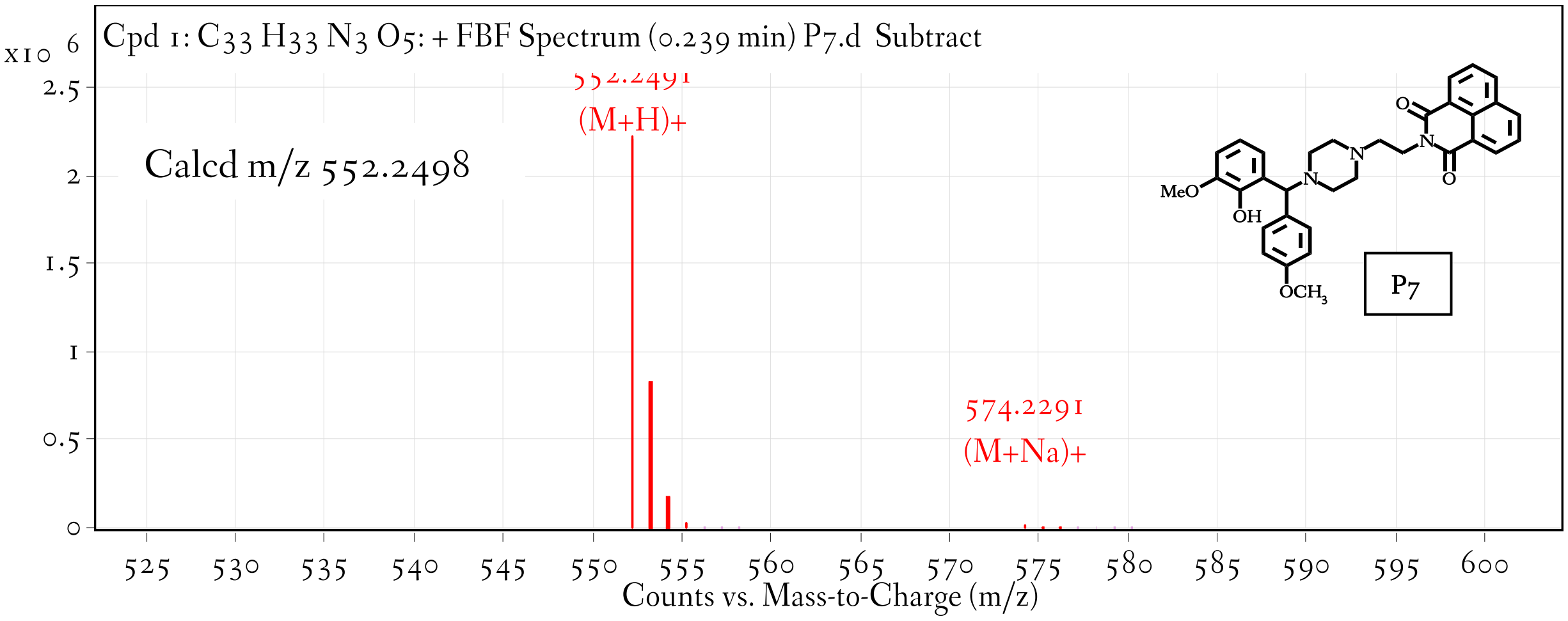

Cpd 1: C33 H33 N3 O5: + FBF Spectrum (0.239 min) P7.d Subtract
x10 6
Calcd m/z 552.2498
(M+H)+
574.2291
(M+Na)+
MeO
OH
OCH3
P7
Counts vs. Mass-to-Charge (m/z)

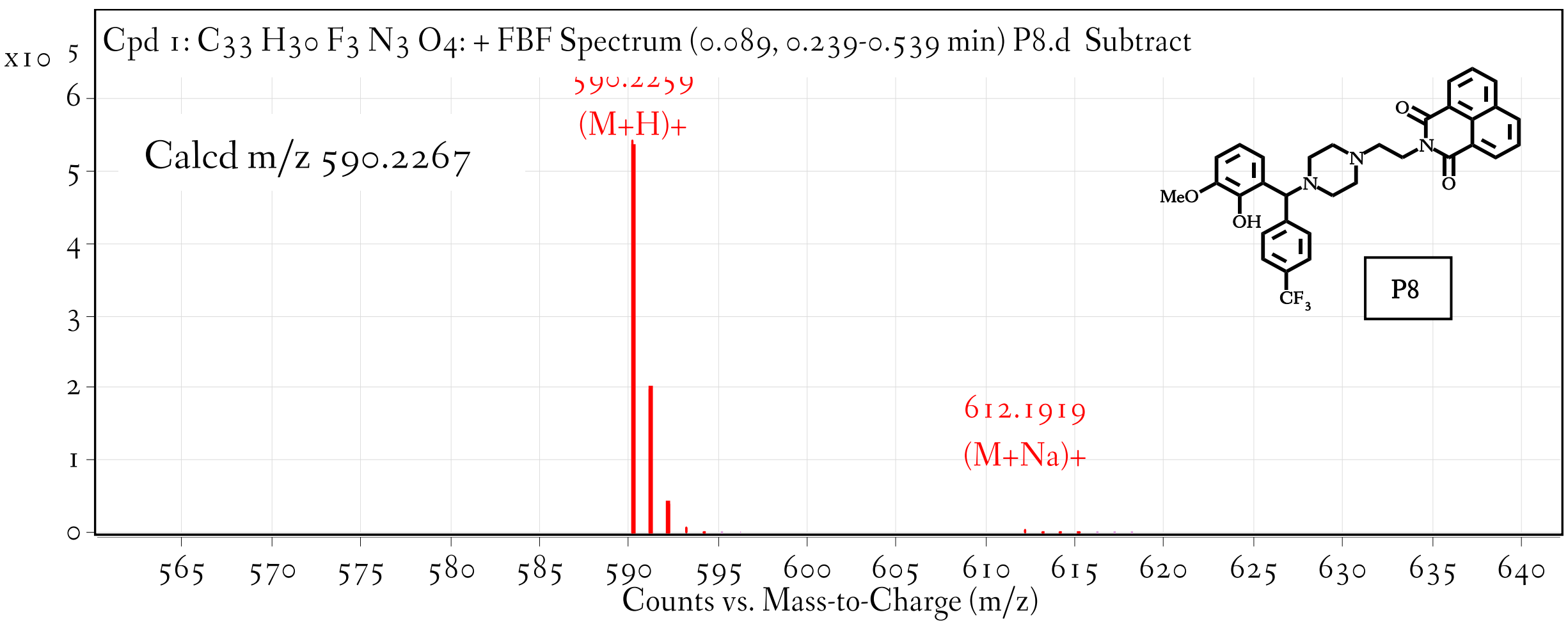

Cpd 1: C33 H30 F3 N3 O4: + FBF Spectrum (0.089, 0.239-0.539 min) P8.d Subtract
x10 5
Calcd m/z 590.2267
(M+H)+
612.1919
(M+Na)+
MeO
OH
CF3
P8
Counts vs. Mass-to-Charge (m/z)

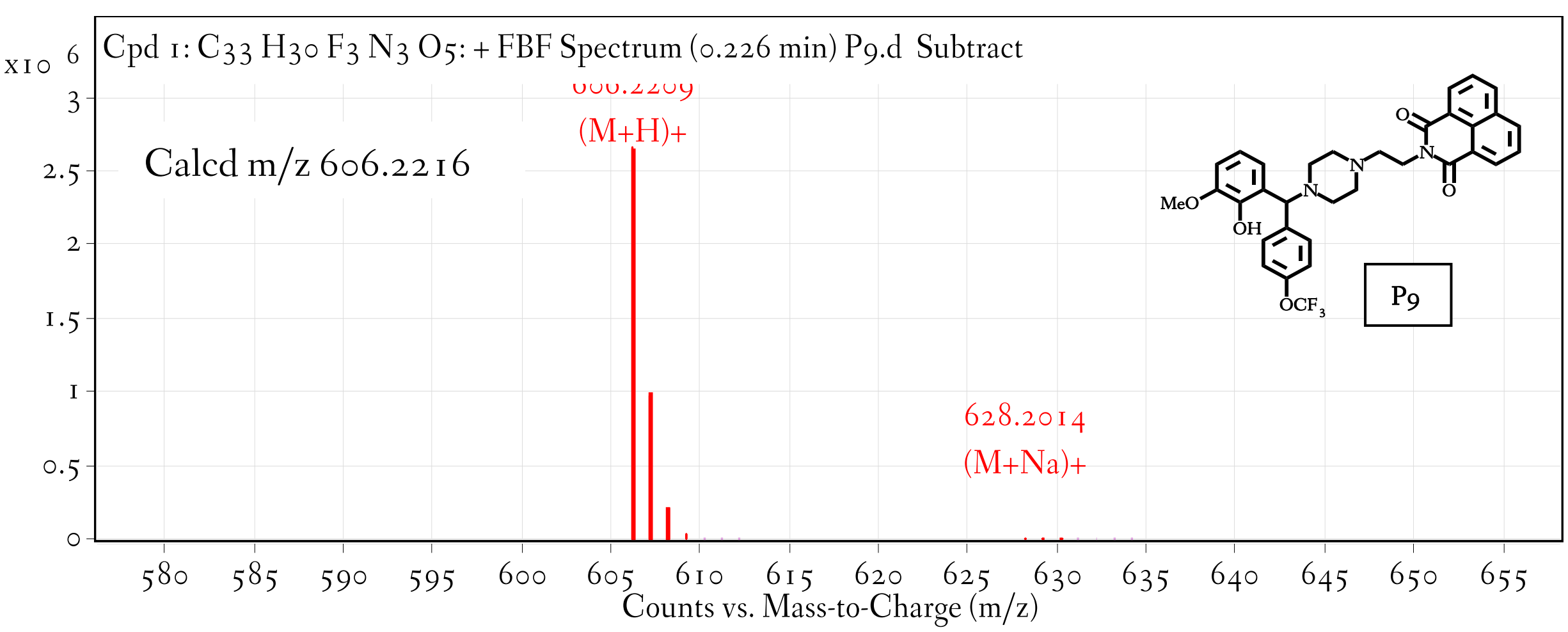

Cpd 1: C33 H30 F3 N3 O5: + FBF Spectrum (0.226 min) P9.d Subtract
x10 6
Calcd m/z 606.2216
(M+H)+
628.2014
(M+Na)+
MeO
OH
OCF3
P9
Counts vs. Mass-to-Charge (m/z)

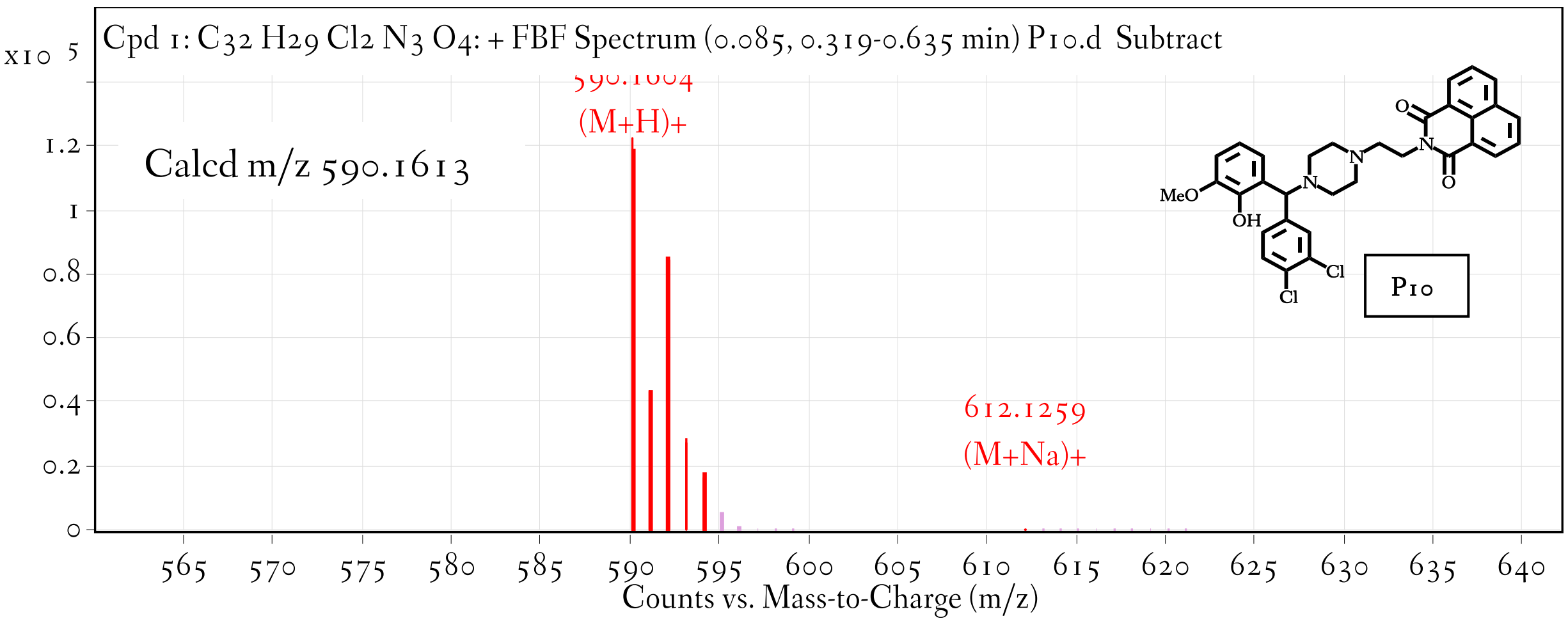
Cpd 1: C32 H29 Cl2 N3 O4: + FBF Spectrum (0.085, 0.319-0.635 min) P10.d Subtract
x10 5
Calcd m/z 590.1613
(M+H)+
612.1259
(M+Na)+
MeO
OH
Cl
Cl
N
N
N
O
O
P10
Counts vs. Mass-to-Charge (m/z)

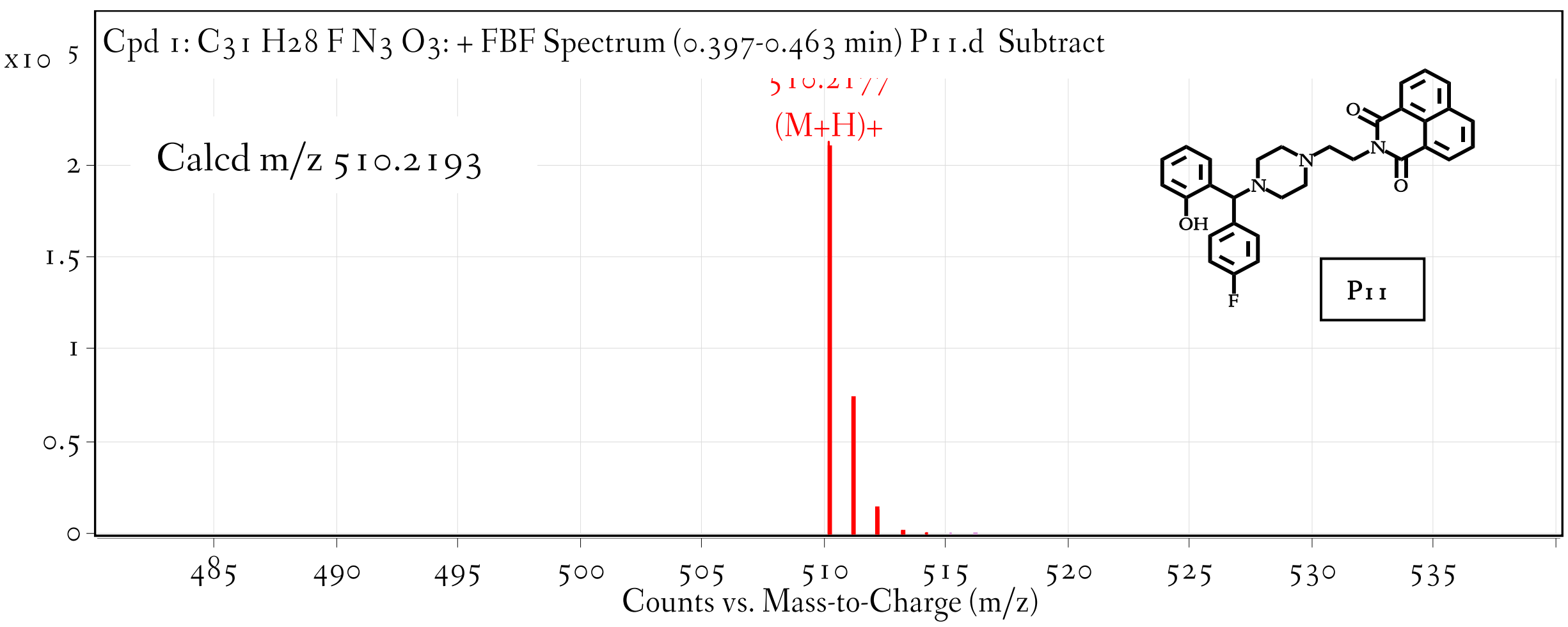
Cpd 1: C31 H28 F N3 O3: + FBF Spectrum (0.397-0.463 min) P11.d Subtract
x10 5
Calcd m/z 510.2193
(M+H)+
OH
F
N
N
N
O
O
P11
Counts vs. Mass-to-Charge (m/z)

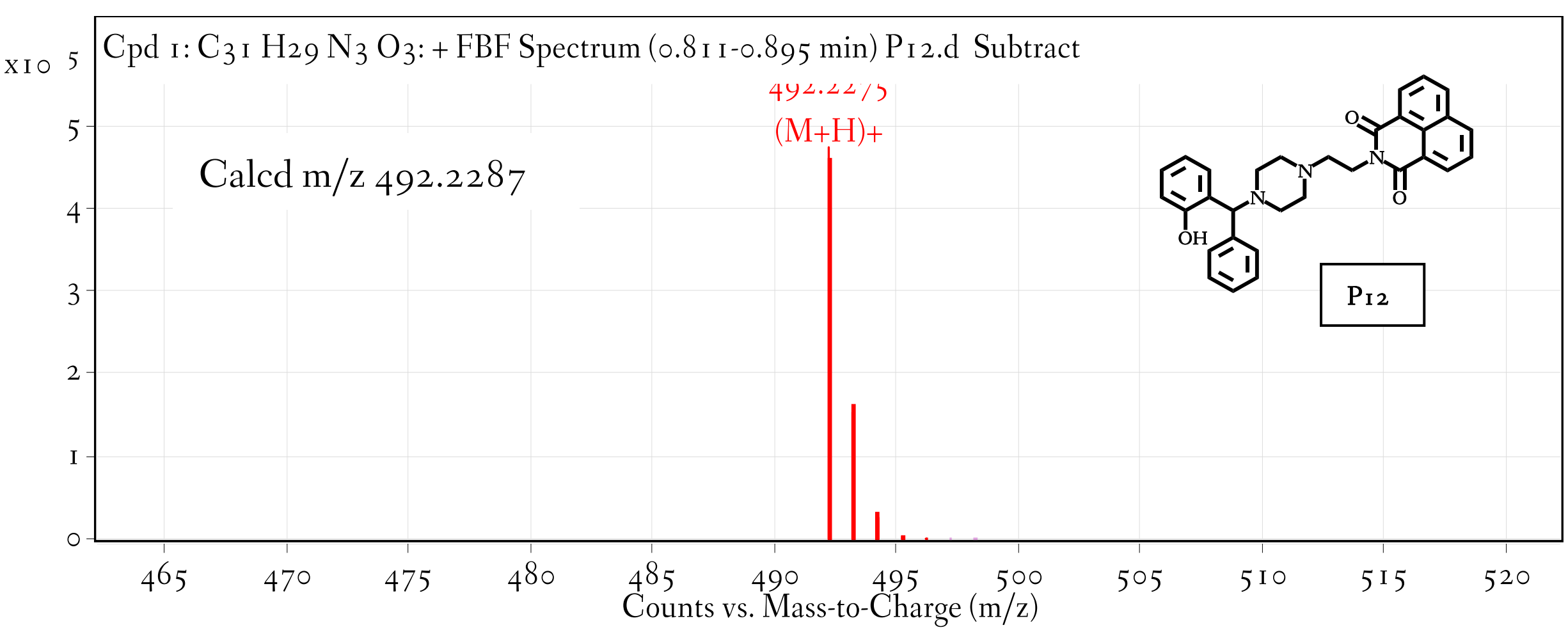
Cpd 1: C31 H29 N3 O3: + FBF Spectrum (0.811-0.895 min) P12.d Subtract
x10 5
Calcd m/z 492.2287
(M+H)+
OH
N
N
N
O
O
P12
Counts vs. Mass-to-Charge (m/z)

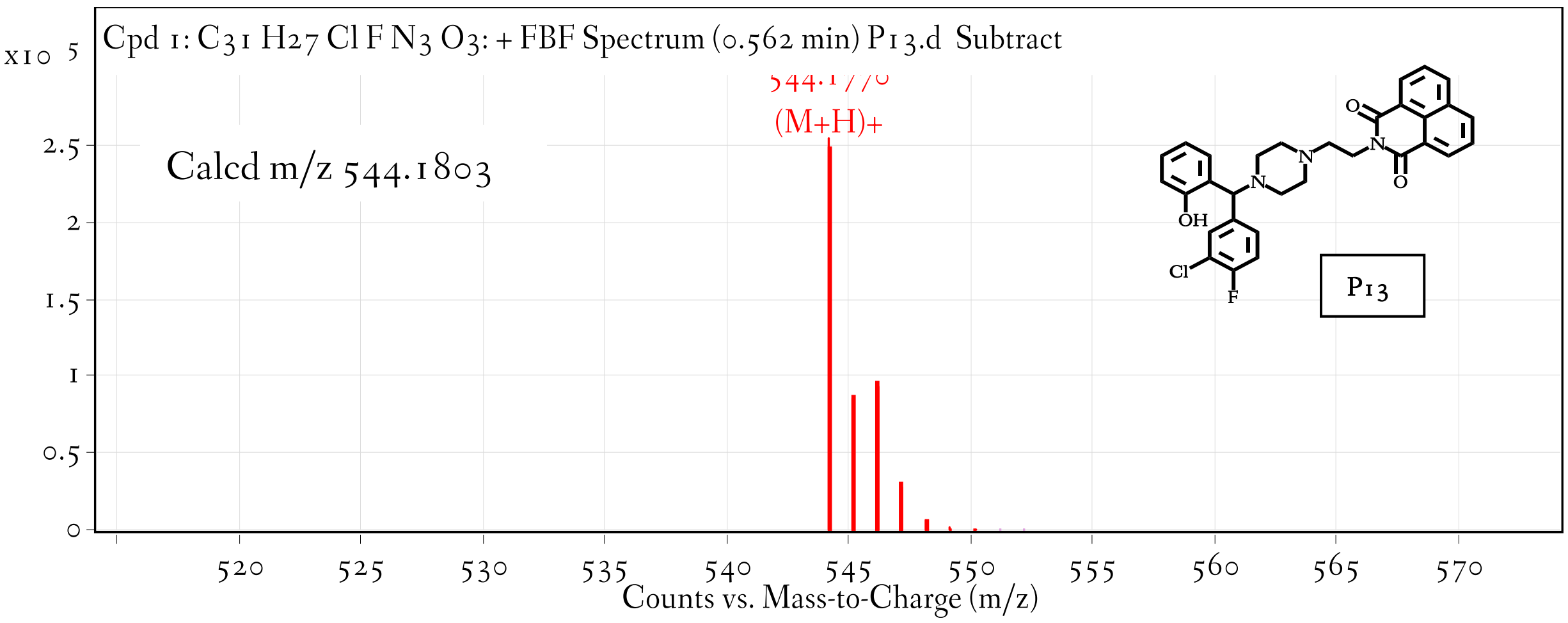
Cpd 1: C31 H27 Cl F N3 O3: + FBF Spectrum (0.562 min) P13.d Subtract
x10 5
Calcd m/z 544.1803
(M+H)+
OH
Cl
F
P13
Counts vs. Mass-to-Charge (m/z)

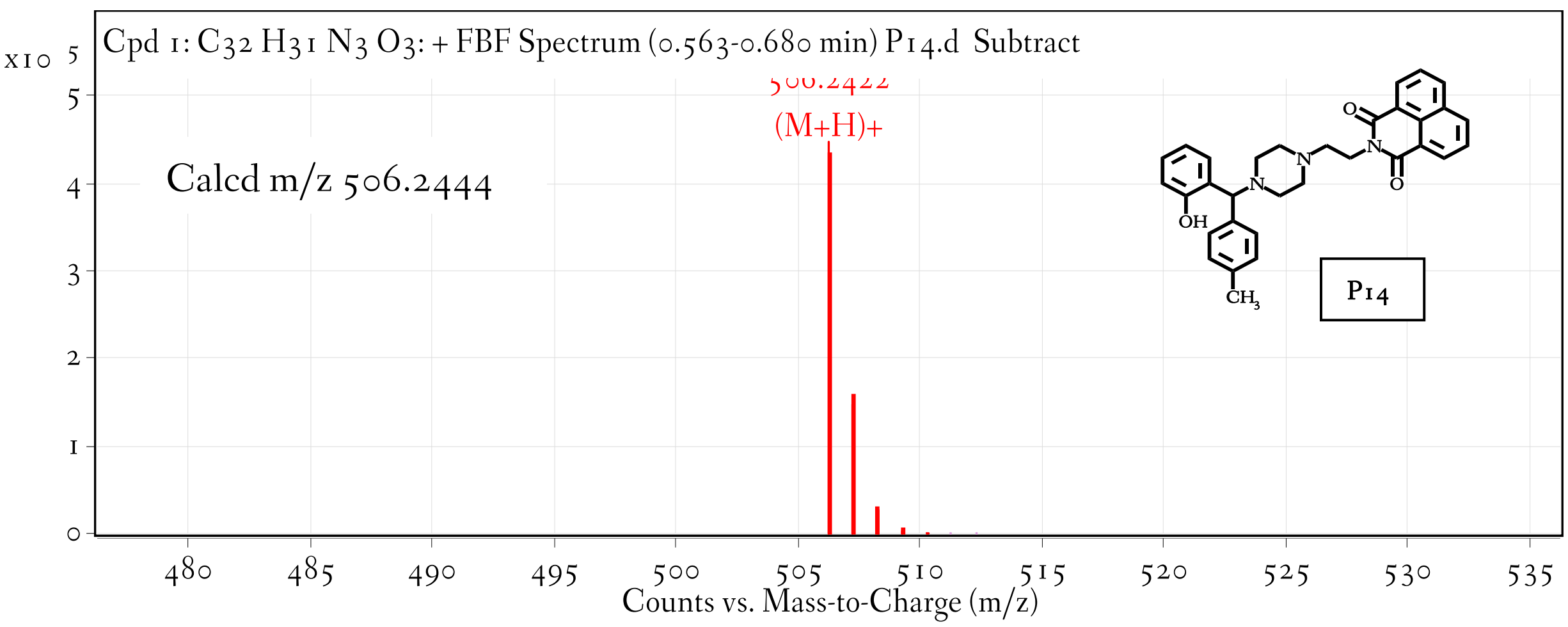
Cpd 1: C32 H31 N3 O3: + FBF Spectrum (0.563-0.680 min) P14.d Subtract
x10 5
Calcd m/z 506.2444
(M+H)+
OH
CH3
P14
Counts vs. Mass-to-Charge (m/z)

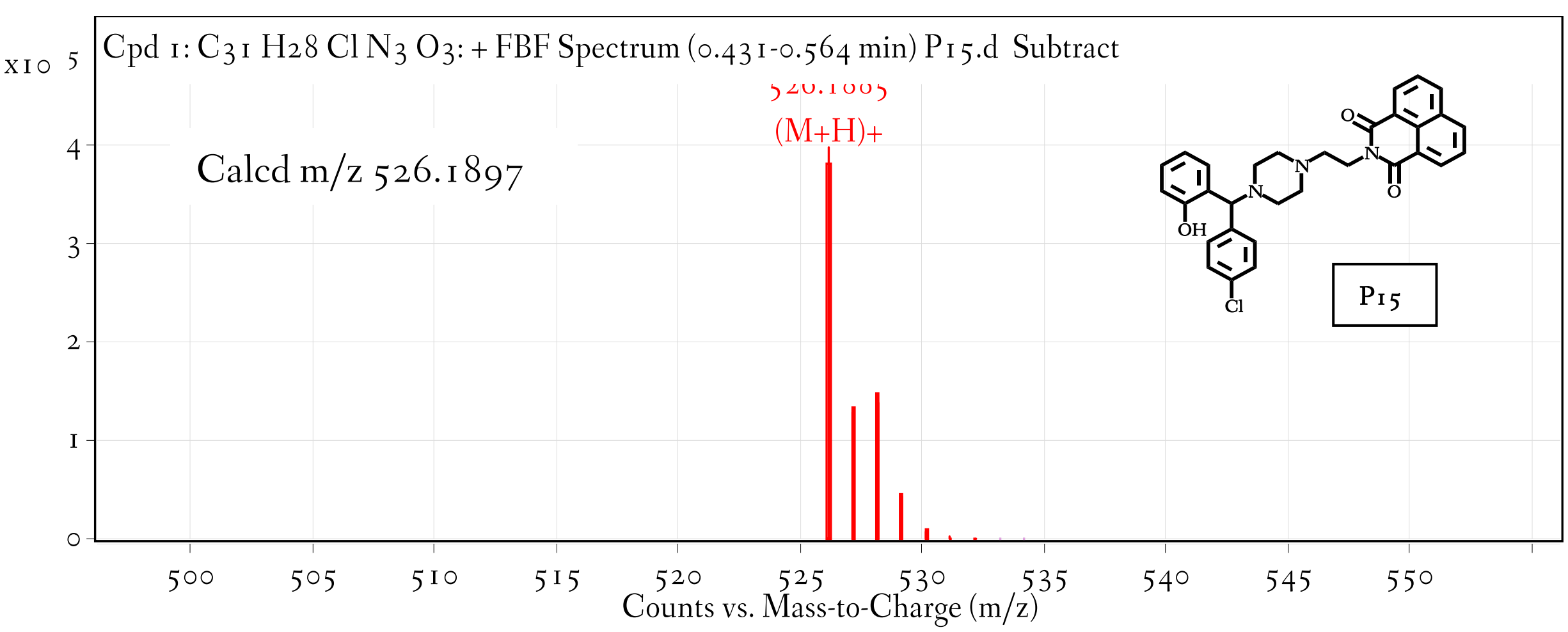
Cpd 1: C31 H28 Cl N3 O3: + FBF Spectrum (0.431-0.564 min) P15.d Subtract
x10 5
Calcd m/z 526.1897
(M+H)+
OH
Cl
P15
Counts vs. Mass-to-Charge (m/z)

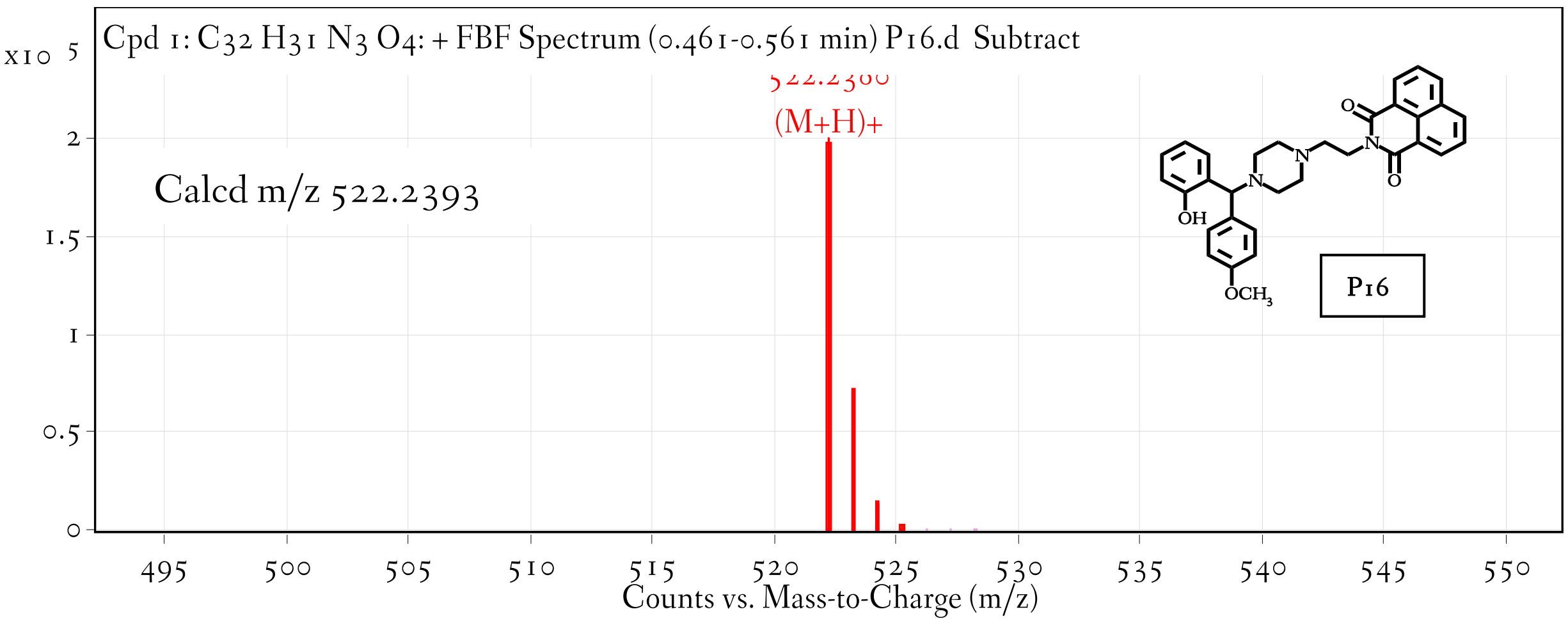
Cpd 1: C32 H31 N3 O4: + FBF Spectrum (0.461-0.561 min) P16.d Subtract
x10 5
(M+H)+
Calcd m/z 522.2393
OH
OCH3
N
O
P16
Counts vs. Mass-to-Charge (m/z)

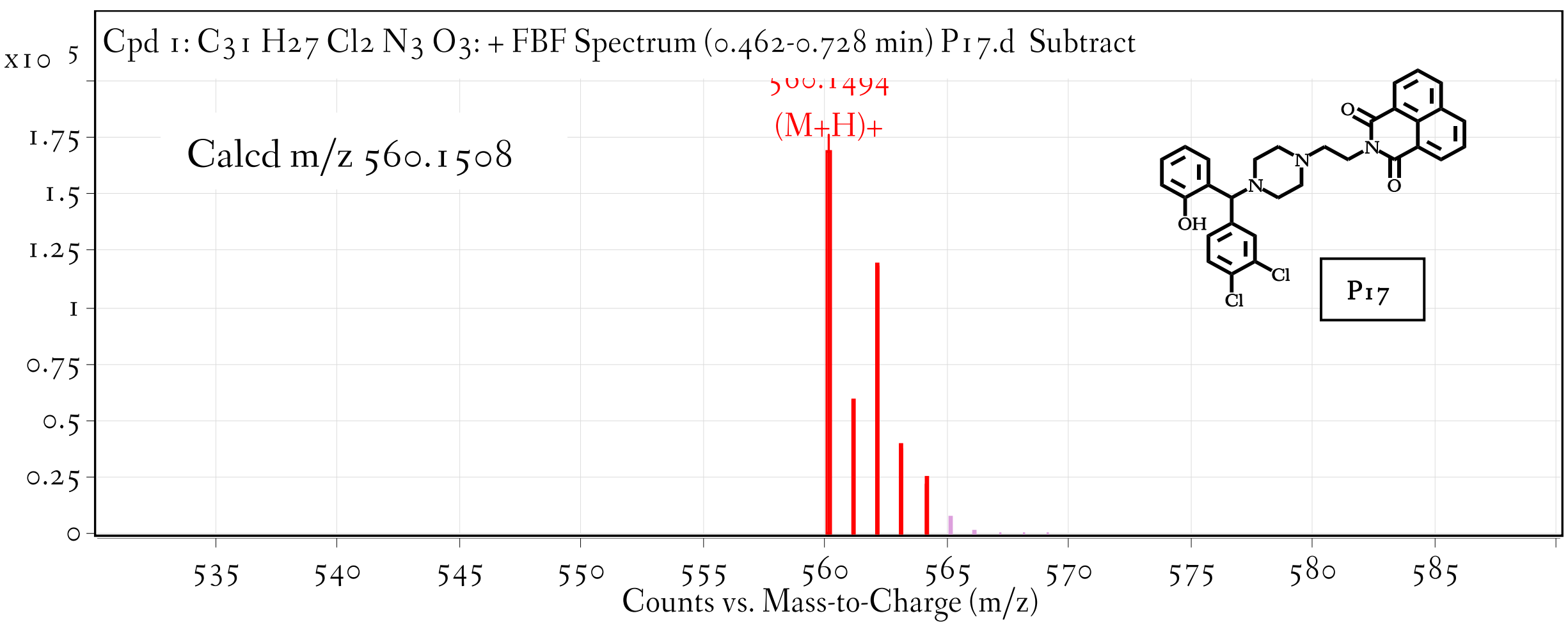
Cpd 1: C31 H27 Cl2 N3 O3: + FBF Spectrum (0.462-0.728 min) P17.d Subtract
x10 5
(M+H)+
Calcd m/z 560.1508
OH
Cl
N
O
P17
Counts vs. Mass-to-Charge (m/z)

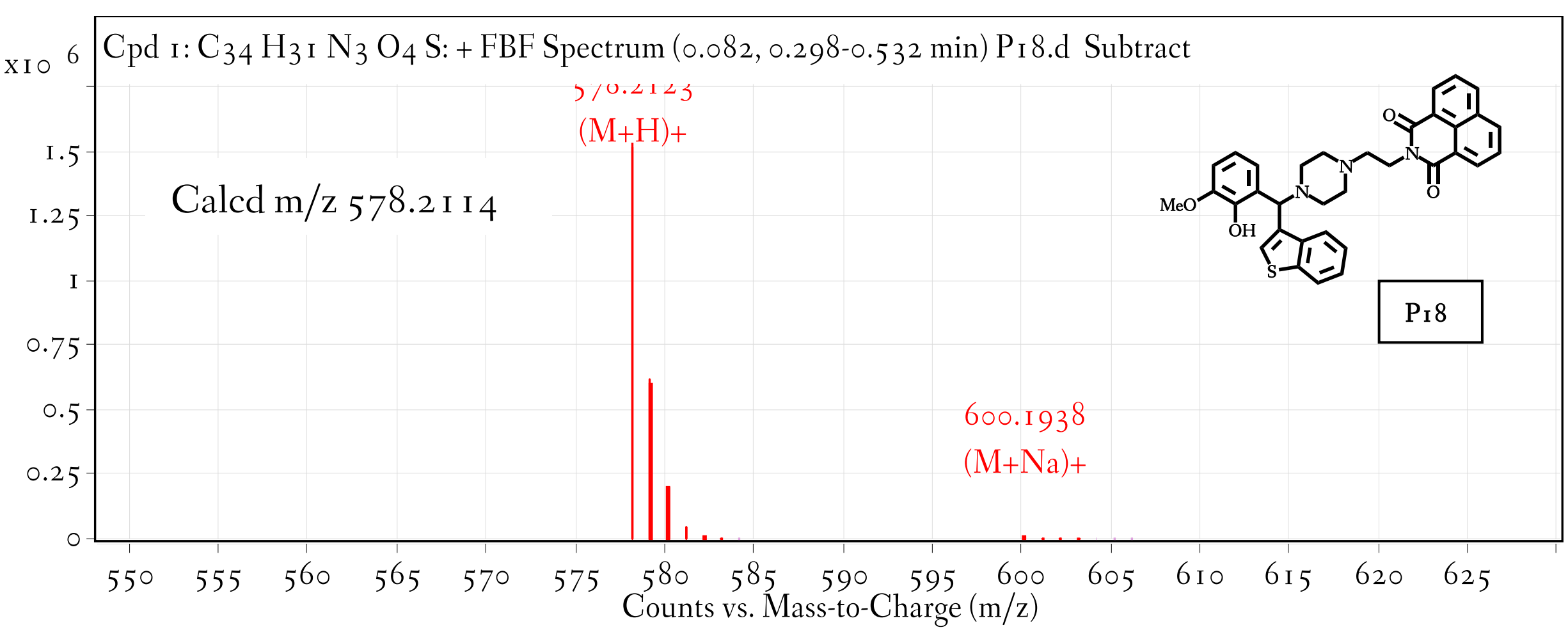
Cpd 1: C34 H31 N3 O4 S: + FBF Spectrum (0.082, 0.298-0.532 min) P18.d Subtract
x10 6
(M+H)+
Calcd m/z 578.2114
600.1938
(M+Na)+
MeO
OH
S
N
O
P18
Counts vs. Mass-to-Charge (m/z)

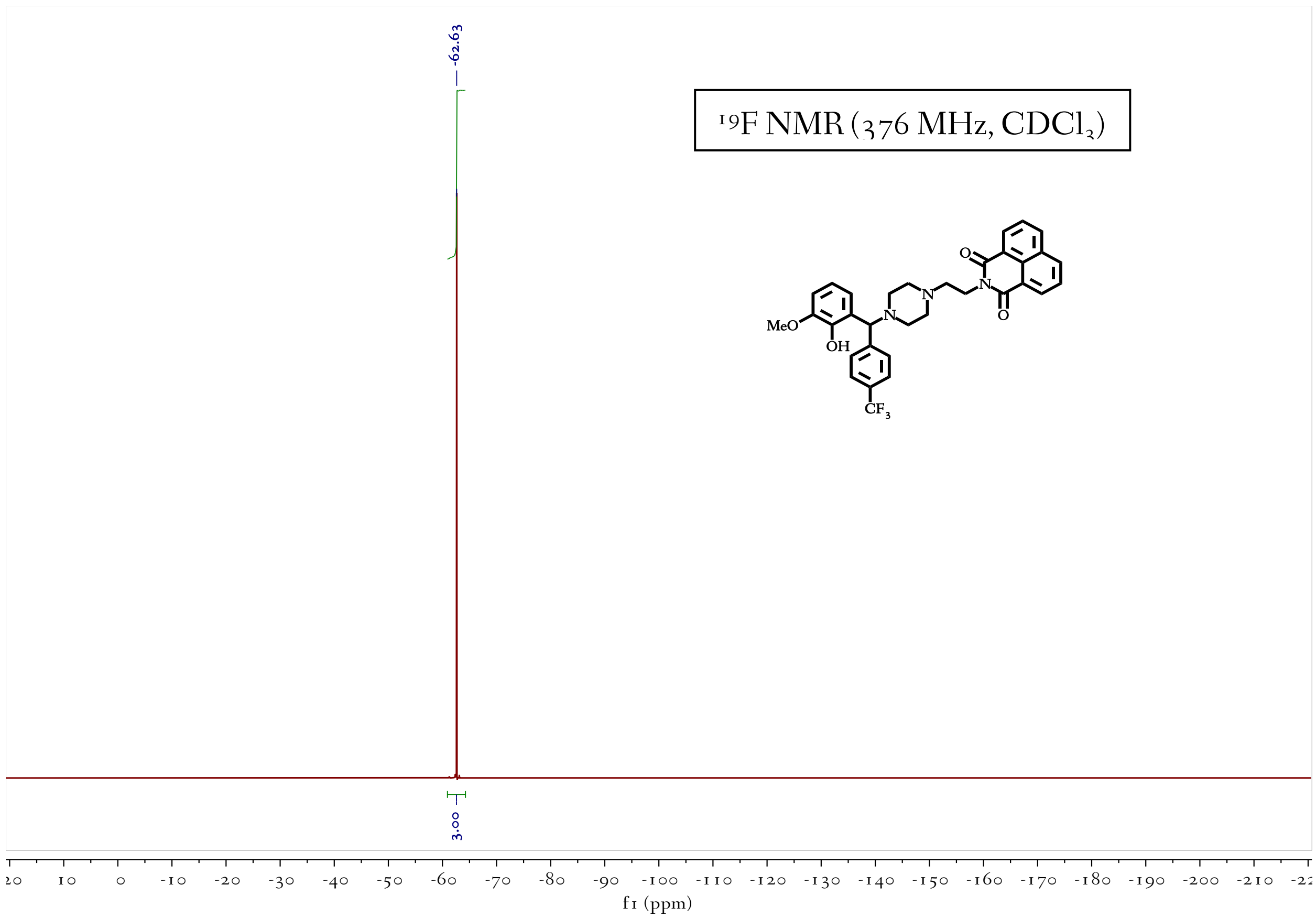
-62.63
¹⁹F NMR (376 MHz, CDCl₃)
MeO
OH
N
N
N
O
O
CF₃
3.00
20 10 0 -10 -20 -30 -40 -50 -60 -70 -80 -90 -100 -110 -120 -130 -140 -150 -160 -170 -180 -190 -200 -210
f1 (ppm)
